# Development of an Automated Workflow for Screening the Assembly and Host–Guest Behavior of Metal‐Organic Cages Towards Accelerated Discovery

**DOI:** 10.1002/anie.202424270

**Published:** 2025-04-17

**Authors:** Annabel R. Basford, Aaron H. Bernardino, Paula C. P. Teeuwen, Benjamin D. Egleston, Joshua Humphreys, Kim. E. Jelfs, Jonathan R. Nitschke, Imogen A. Riddell, Rebecca L. Greenaway

**Affiliations:** ^1^ Department of Chemistry, Imperial College London, Molecular Sciences Research Hub White City Campus Wood Lane London W12 0BZ UK; ^2^ Yusuf Hamied Department of Chemistry University of Cambridge Lensfield Road Cambridge CB2 1EW UK; ^3^ Department of Chemistry University of Manchester Oxford Road Manchester M13 9PL UK

**Keywords:** Automation, Host–Guest behavior, Metal‐Organic cages, Self‐assembly

## Abstract

Metal‐organic cages (MOCs) are a class of self‐assembled materials with promising applications in chemical purifications, sensing, and catalysis. Their potential is, however, hampered by challenges in the targeted design of MOCs with desirable properties. MOC discovery is thus often reliant on trial‐and‐error approaches and brute‐force manual screening, which are time‐consuming, costly, and material‐intensive. Translating the synthesis and property screening of MOCs to an automated workflow is therefore attractive, to both accelerate discovery and provide the datasets crucial for data‐led approaches to accelerate MOC discovery and to realize their targeted properties for specific applications. Here, an automated workflow for the streamlined assembly and property screening of MOCs was developed, incorporating automated high‐throughput screening of variables pertinent to MOC synthesis, data curation and automated analysis, and development of a host–guest assay to rapidly assess binding behavior. Computational modelling supplemented this automated experimental workflow for *post priori* rationalization of experimental outcomes. This study lays the groundwork for future large‐scale MOC screening: from a relatively modest screen of 24 precursor combinations under one set of reaction conditions, 3 clean MOC species were identified, and subsequent screening of their host–guest behavior highlighted trends in binding and the identification of potential applications in molecular separations.

## Graphical abstract

An automated experimental workflow to accelerate the discovery of metal‐organic cages (MOCs), which is often hindered by slow, manual approaches, has been developed. Automation of the synthesis, characterization, and analysis, is supported by computational modelling to rationalize the experimental outcomes, and development of a host–guest assay elucidated trends in MOC binding behavior.

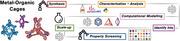

## Introduction

Metal‐organic cages (MOCs) are modular supramolecular structures that are prepared from both organic and inorganic building blocks through self‐assembly. Metal complexes typically form the vertices of these polyhedral architectures, while organic linkers either span the faces or the edges. Their internal cavities are capable of binding guests with high affinity and selectivity, enabling discrimination between chemically similar molecules and allowing potential applications in chemical purification,^[^ ^1^, ^2^, ^3^ ^]^ sensing,^[^ ^4^, ^5^, ^6^, ^7^ ^]^ and catalysis.^[^ ^8^, ^9^, ^10^, ^11^, ^12^ ^]^ Generally, new MOCs are designed based on concepts uncovered in previous work, where new structures are created by carrying out small alterations to the building blocks and tuning the assembly conditions. The structural diversity of reported MOCs has been achieved through changing the topicity, shape, or flexibility of the ligand, the metal ion or counterion identity, or the solvent, temperature, or concentration.^[^ ^13^, ^14^ ^]^ For example, a small change in building block geometry can lead to a major impact on the resulting MOC architecture and characteristics.^[^ ^13^, ^15^, ^16^ ^]^ The exploratory and serendipitous nature of MOC discovery therefore often leads to wasted resources when no stable structures are observed under a range of reaction conditions. The application of both automated experimental and computational screening, however, has the potential to help accelerate this design and discovery process.

Previously, our work has focused on the automated discovery of organic supramolecular assemblies, including porous organic cages and catenanes, which was further streamlined into a low‐cost, open‐source workflow which combined automated synthesis, characterization, and analysis.^[^ ^17^, ^18^ ^]^ This workflow was designed to be translatable to other supramolecular architectures. Computational investigations of MOCs to derive design rules and fundamental understanding are still relatively scarce, but notable contributions from Piskorz et al. and Tarzia et al. demonstrate the value of using computational screening to better understand the MOC self‐assembly process, enabling desired architectures to be targeted and experimental outcomes to be rationalized.^[^ ^19^, ^20^ ^]^

To the best of our knowledge, there is only one recent example demonstrating translation of the assembly and host–guest screening of MOCs into an automated mobile workflow,^[^ ^21^ ^]^ and the application of automated high‐throughput screening and analysis methods to the MOC discovery process has not yet been thoroughly demonstrated. Additionally, most reported MOC studies only show a subset of successful reaction parameters, typically not including those that led to “failures”. Often, the self‐assembly results of reactions that either yielded the same structures, or indeed no stable structures, are not reported, despite providing valuable information for future work. The methods explored in this work will allow for the creation of a more complete picture of the self‐assembly landscape. Not only will an automated workflow accelerate the experimental screening process, the creation of MOC self‐assembly data in this way will be important for subsequent use in machine‐learning (ML) methods toward MOC structure and property prediction. Uptake of ML methods using this type of data will underpin the future discovery of MOCs with valuable properties, to provide solutions for key industrial processes governed by host–guest binding.

**Figure 1:**
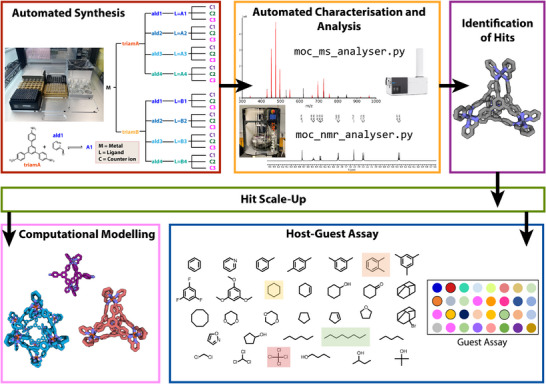
Summary of the automated workflow for the assembly and property screening of metal‐organic cages (MOCs): (Top left, red) High‐throughput automated experimental synthesis, including the setup of an Opentrons OT‐2 liquid handing platform for screening the assembly of a metal ion (M) with two triamines (A and B) and four aldehydes (1–4), yielding eight ligands (L = A1, A2, A3, A4, B1, B2, B3, and B4), screened with three counterions (C); (Top middle, orange) automated characterization and analysis of ^1^H NMR spectra and high‐resolution mass spectrometry to assess the assembly outcomes from the synthetic screen; (Top right, purple) outputs of the automated data analysis were used to identify interesting “hits” to take forward for property screening; (Middle, green) conventional batch scale‐up of any identified hits; (Bottom left, pink) computational modelling for visualization and to help rationalize experimental outcomes; and (Bottom right, blue) automated host‐guest assay of 32 guests to probe binding properties of the scaled up hits from the automated screen.

Here we utilize and expand on our previously developed low‐cost automated workflow for organic cages and establish its applicability to discrete metal‐organic assemblies (Figure 1). Automated screening of the self‐assembly conditions for a representative family of MOCs is demonstrated, followed by automated characterization of the reaction mixtures by using high‐resolution electrospray ionization mass spectrometry and ^1^H NMR spectroscopy. Analysis of the reaction mixtures is further aided by automated data analysis. Computational modelling was also performed to support experimental outcome rationalization and identify stability trends. Following subsequent scale‐up of a handful of MOCs, the automated workflow was then expanded to include an automated host–guest assay for the rapid screening and identification of promising guest binders.

## Results and Discussion

### Automated Synthetic Screen

First, a face‐capped **M_4_L_4_** tetrahedral (*T*‐symmetric) MOC was chosen as a representative MOC assembly to study in the workflow. The **Zn_4_L_4_** MOC selected is known to form via the simultaneous dynamic covalent imine condensation between a triamine and monoaldehyde to generate a ligand (**L**) which coordinates through the pyridyl and imine nitrogens with the Zn(II) metal ions (**M**).^[^ ^22^ ^]^ This **Zn_4_L_4_** MOC architecture was selected due to its potential for strong guest binding,^[^ ^23^ ^]^ and because derivatives have been shown to encapsulate a range of hydrocarbons and anions.^[^ ^24^, ^25^, ^26^ ^]^ In addition, by simply changing the metal from Zn(II) to Fe(II), the self‐assembly of a **M_12_L_12_** *pseudo‐*icosahedral topology was favored, and when a combination of 1,3,5‐tris(4‐aminophenyl)benzene (**A**) and 2‐formyl pyridine (**1**) was used as the linker components, both **M_4_L_4_** and **M_12_L_12_** MOCs were formed depending on the reaction conditions, alongside helicates (**M_2_L_3_**) at lower concentrations.^[^ ^27^ ^]^ By varying the self‐assembly precursor components, our aim was to better understand their influence on the reaction outcome and demonstrate the potential of applying an automated workflow to the labor‐intensive, time‐consuming task of screening MOC reactions for the determination of self‐assembly reaction outcomes.

**Figure 2:**
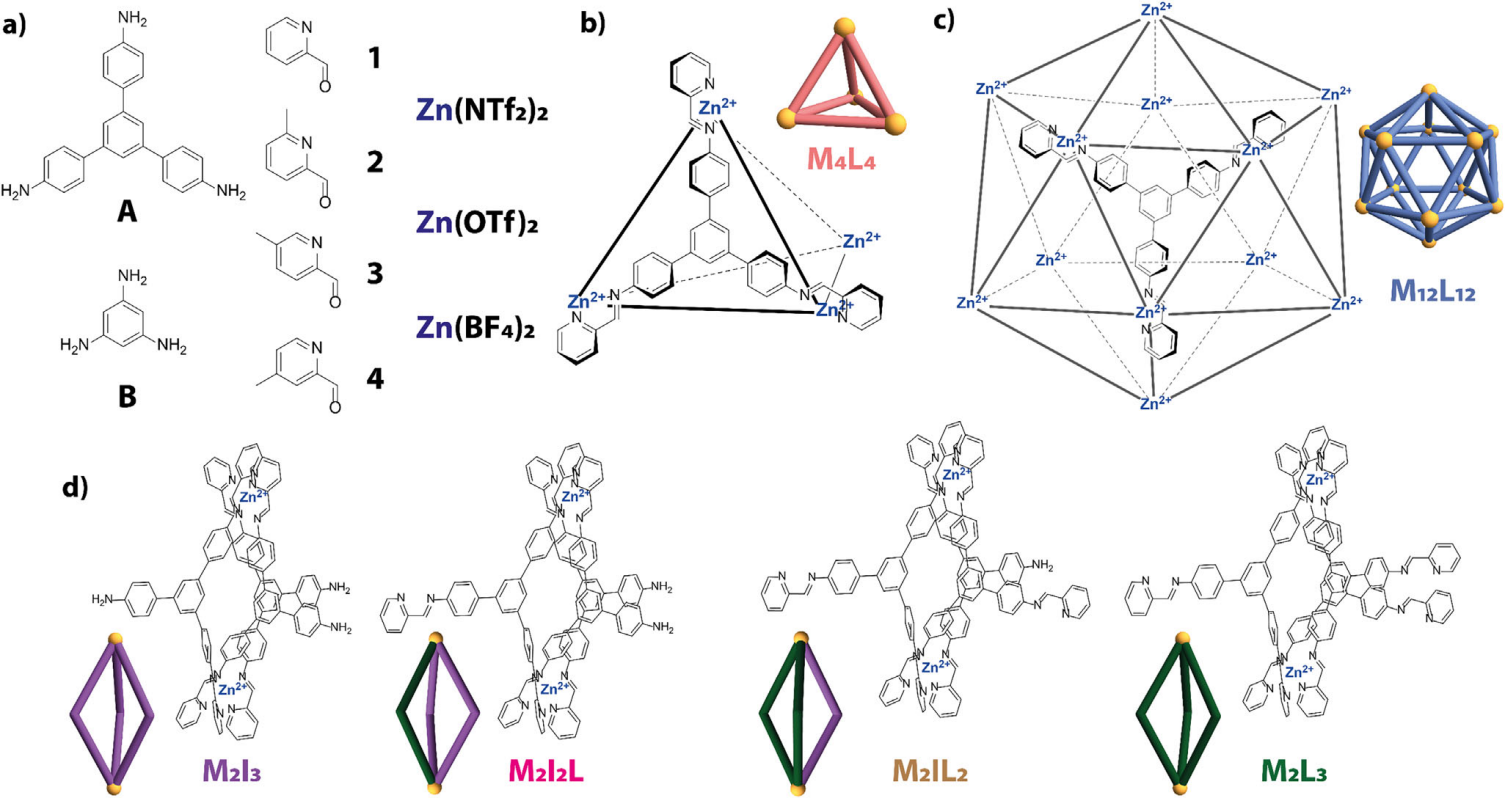
a) Precursors used within the automated synthetic screen for the assembly of metal‐organic cages; b) possible tetrahedral (M_4_L_4_), c) pseudo‐icosahedral (M_12_L_12_), and d) helicate (M_2_I_3_, M_2_I_2_L, M_2_IL_2_, and M_2_L_3_) architectures, which may form when using this combination of precursors in a synthetic screen.

Within the automated synthetic screen, the self‐assembly process was conducted at a 4:12:4 equivalence between the triamine, aldehyde and metal salt building blocks. The precursor library contained two tritopic triamines (**A** and **B**) and four aldehydes with varying methylation on the pyridyl ring (**1**–**4**), leading to 8 potential tritopic imine ligands, named based on their precursor components (**L** = **A1**, **A2**, **A3**, **A4**, **B1**, **B2**, **B3**, and **B4**), and three metal salts with varying counterions: Zn(NTf_2_)_2_, Zn(OTf)_2_, and Zn(BF_4_)_2_ (Figure 2a). The reaction stoichiometry was selected to specifically target a **M_4_L_4_** tetrahedron, however, other self‐assembled products may form including **M_12_L_12_** icosahedra and helicate structures formed from a ligand (**L**) linker and/or an intermediate (**I**) ditopic linker (formed by a two‐fold partial imine condensation between the triamine and aldehyde precursors): **M_2_I_3_**, **M_2_I_2_L**, **M_2_IL_2_**, and **M_2_L_3_** (Figure 2b–d).

Automated reaction preparation was carried out on an Opentrons liquid‐handling platform (OT‐2),^[^ ^28^ ^]^ with the deck containing a 24‐well plate of each precursor stock solution, a 6‐well solvent plate, and a 48‐well reaction plate. A general OT‐2 Python input protocol script was adapted to include the solvent calibrated gantry, aspirate and dispense speeds, along with the stock/solvent locations and move commands dictionaries (full details given in the Supporting Information S2). The reaction plate was subsequently removed from the deck, sealed and stirred at 70 °C for 17 h. A 20 µL sample was then taken, dissolved in MeCN, and taken for HRMS analysis. The bulk reaction mixture solvent was removed with a 48‐well EquaVAP^[^ ^29^ ^]^ and redissolved in acetonitrile‐*d_3_* for ^1^H NMR analysis. Overall, 24 combinations were screened under the same reaction conditions (overall concentration: 0.0092 M, total volume: 1 mL, MeCN, 70 °C, and 17 h) in 2 mL capped vials with stirrer bars, and repeated to check reproducibility.

### Automated Characterization and Analysis

High‐resolution mass spectrometry (HRMS) and high‐throughput ^1^H NMR spectroscopy were selected as data characterization techniques for the automated synthetic screen and used in conjunction with one another. Overall, HRMS analysis indicated reactivity of the precursors and identified whether a **M_N_L_N_** (where *N* = number of incorporated units) or **M_2_X_3_** (where **X** = **L** or **I**) species of interest had formed, while the conversion was analyzed by ^1^H NMR spectroscopy. Where unreacted aldehyde precursor was observed, this indicated that the reaction had not gone to completion. This two‐step approach filters the results from the screen, with only full characterization conducted on any “hits”, although assignment of the ^1^H NMR spectra was attempted for all spectra collected to deduce the reaction outcome and identify the major assembly. To streamline the analysis of the characterization data, the open‐source code from our cage database tool *cagey* was adapted to create both moc_ms_analyser.py and moc_nmr_analyser.py (https://github.com/GreenawayLab/development‐automated‐workflow‐mocs), enabling automated analysis.

First, a Python script was developed to calculate likely possible structures that may form between the six‐coordinate metal ion, tri‐topic ligand and the di‐topic intermediate (where the triamine has undergone two imine condensation reactions with a given aldehyde but a free amine remains), and with a varying number of counterions. For each reaction, the metal, triamine, aldehyde, and counterion SMILES were loaded as a Python dictionary and the empirical formulae were calculated for the metal ion, tritopic ligand, ditopic intermediate, and counterion. The combinations of these components into possible assemblies, with their corresponding empirical formulae, charges, and isotope splitting patterns, were subsequently calculated. The experimental HRMS data was then searched for the *m/z* isotope splitting of each of the calculated possible solutions, and if found, written to a data frame containing: the formula of the structure, found *m/z*, found intensity, predicted *m/z*, predicted intensity, and predicted charge. The data frame was then ordered by formula and the *m/z* splitting between the peaks was calculated to assign the charge. The results were filtered down to formulae found where the observed charge matched the predicted charge, yielding a machine‐readable data frame of the HRMS results.

**Figure 3:**
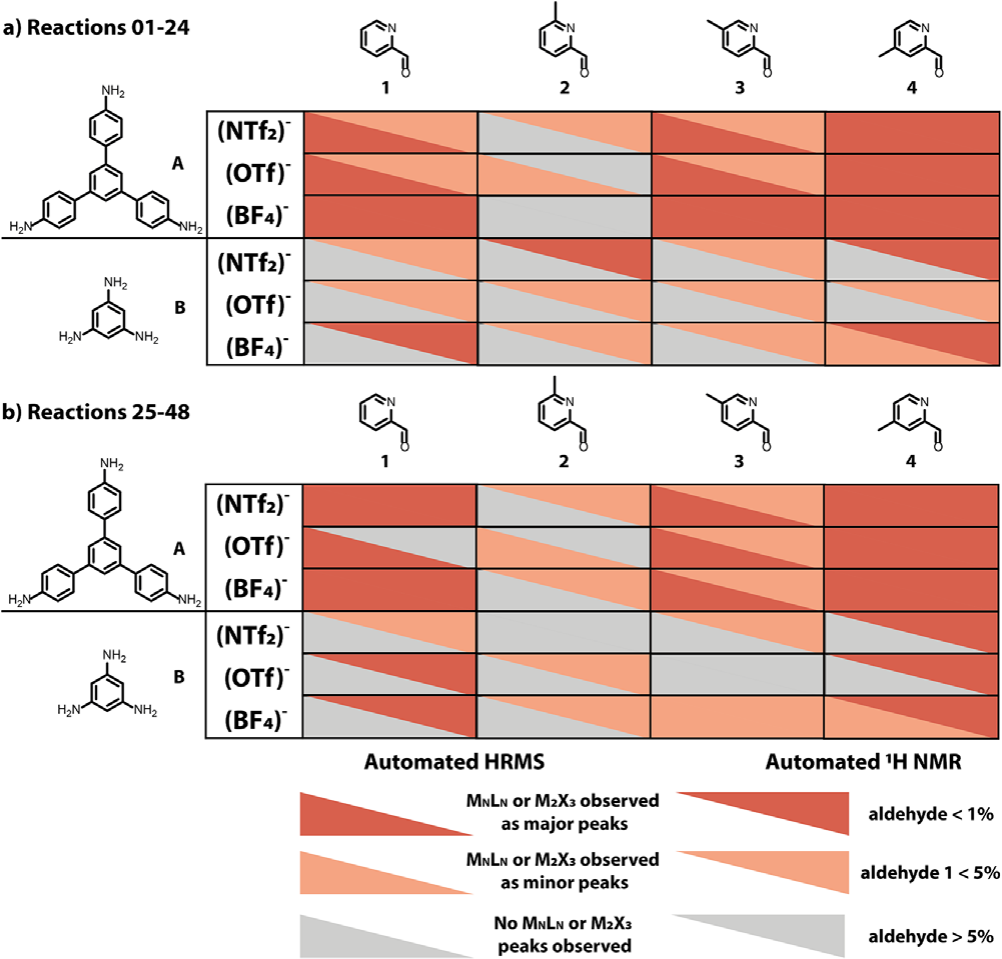
Overall results from the automated synthesis screen based on the automated analysis of HRMS data to identify target structures or fragments (M_N_L_N_ and M_2_X_3_) as either the major (red) or minor (orange) assembly, or not identified (grey), and of ^1^H NMR spectra for conversion based on residual aldehyde, where aldehyde <1% observed (red), 1%–5% (orange) and >5% (grey). a) reactions 01–24 results, and b) repeat reactions 25–48. A fully red box is indicative of the ideal outcome from the automated analysis, where a reaction has <1% aldehyde remaining and a target structure has been identified as a major peak in the HRMS.

Next, the ^1^H NMR data was analyzed for conversion, where any residual aldehyde signals indicated that the dynamic covalent reaction had not gone to full conversion. Both cage peaks and any residual aldehyde were automatically identified, using the *moc_nmr_analyser*.*py* Python script. The script was based on that from *cagey*, but further adapted to include the NMR solvent acetonitrile‐*d_3_*, as it was previously only suitable for chloroform‐*d*. The user benefits from providing one path to the folder of the NMR raw data as the input, and then a CSV output labelled by the raw files title name is written containing the peak type, peak shift in ppm, and amplitude. If any residual aldehyde was observed, a further qualitative analysis was performed to determine the percentage of remaining aldehyde which was categorized and outputted as a JSON database file. If the relative percentage of the aldehyde to the largest cage or imine peak was below 1% it was categorized as “minor”, if between 1% and 5% it was categorized as “still considered for scale‐up”, and if above 5% categorized as ‘conversion not satisfactory’. This additional step is qualitative as no internal standard was used but provides additional insight when identifying “hits” to scale up. Although this level of automated analysis is not suitable for full characterization and identification of the exact MOC topological outcome, it does streamline the screening process by giving an indication of whether a targeted species has formed and the conversion of the reaction (Figure 3), saving researcher's effort and time.

Overall, the reproducibility between the two replicate screens was in broad agreement – 23/24 (96%) resulted in the same automated reactivity analysis by HRMS. Taking into account minor changes in conversion ranging from <1% to <5%, 20/24 reactions gave reproducible ^1^H NMR spectra. Of all the reactions, 75% of precursor combinations containing triamine **A** resulted in the formation of the **M_N_L_N_** or **M_2_X_3_** targeted topologies as the major mass ion peak, whereas none of the combinations with triamine **B** resulted in the targeted species being identified in the HRMS spectra, suggesting a larger linker core is required to form a stable self‐assembled species. Of the triamine **B** combinations, a higher proportion of ligand and intermediates were identified in the HRMS spectra (Supporting Information S2.3), indicating that while self‐assembly with the metal ion was not successful, the imine condensations between **B** and the aldehydes had occurred. This conclusion was further confirmed by the observation that 92% of the reactions with triamine **B** showed high conversion based on the automated ^1^H NMR analysis. This observation highlights the need for multiple characterization methods to be used in parallel for interpretation and determination of the outcome from automated high‐throughput screens. Finally, of the precursor combinations containing triamine **A**, a clear effect on the assembly outcome can be observed on incorporating a methyl group and on varying its positional isomerism (*ortho*, *meta*, or *para* to the pyridyl nitrogen): when the methyl substituent is introduced in the *para*‐position (**4**), far away from where metal coordination occurs, the assembly outcome is similar to the base pyridyl‐aldehyde (**1**), but the likelihood of successfully forming the targeted **M_N_L_N_** or **M_2_X_3_** species reduces as the methyl group is moved around the ring from the *para‐* to *meta‐* (**3**) to *ortho‐*positions (**2**). We attributed this observation to increased steric hinderance around the metal center on co‐ordination, which reduces the likelihood of MOC formation.

### Identification and Scale‐up of “Hits”

**Figure 4:**
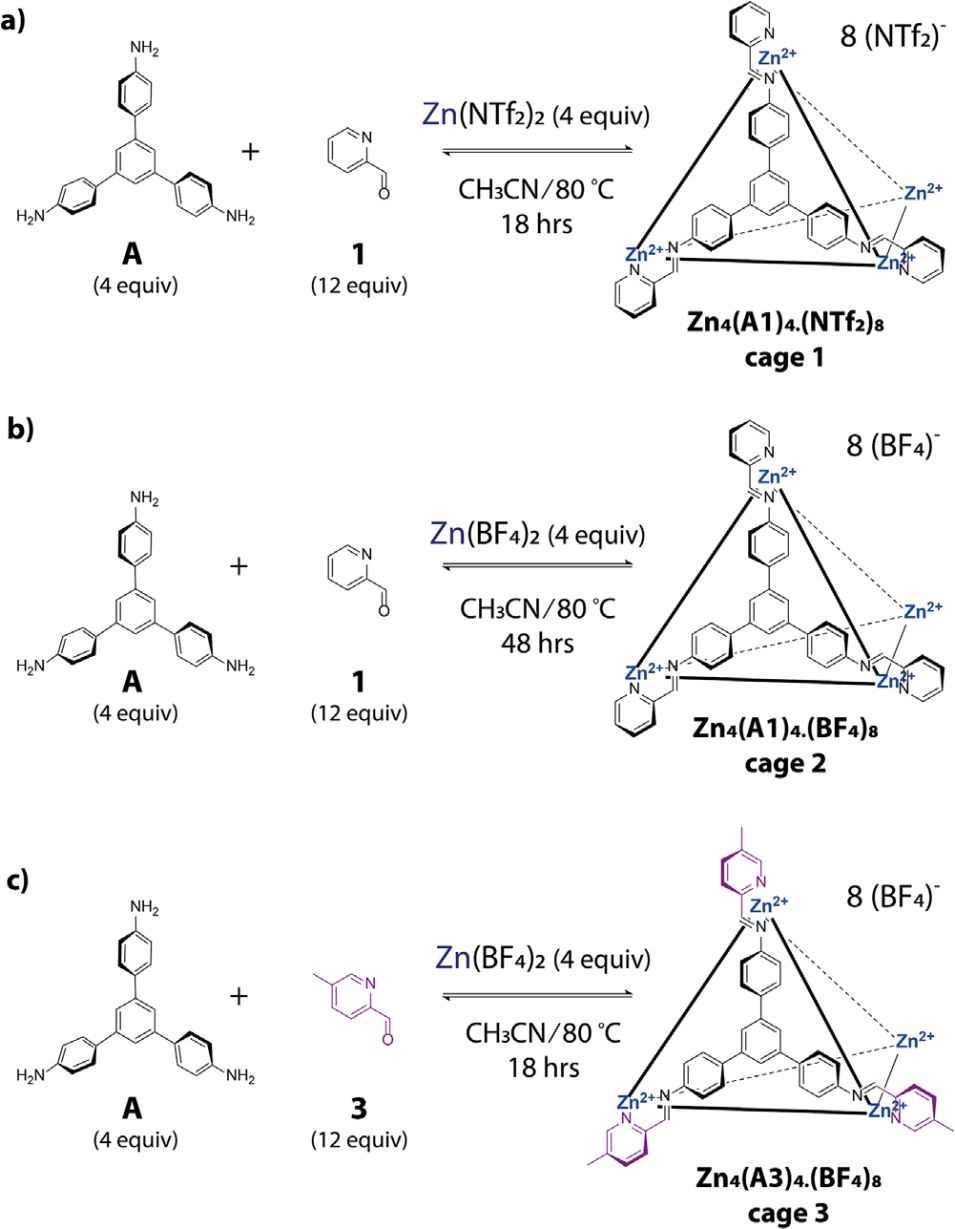
Scale‐up syntheses of the hit M_4_L_4_ tetrahedron cages: a) Zn_4_(A1)_4_.(NTf_2_)_8_ (**cage 1**); b) Zn_4_(A1)_4_.(BF_4_)_8_ (**cage 2**); and c) Zn_4_(A3)_4_.(BF_4_)_8_ (cage 3).

On filtering of the results, six precursor combinations were identified as “hits” where the automated HRMS analysis identified the targeted topological mass ions as major species and automated ^1^H NMR analysis identified high conversion with <1% residual aldehyde remaining. However, while the automated HRMS analysis identifies any targeted **M_N_L_N_** or **M_2_X_3_** assemblies, the latter **M_2_X_3_** species may also form from fragmentation of larger topologies during the analysis.^[^ ^30^ ^]^ For example, a **M_2_L_3_** mass ion may be from a helicate or mesocate structure, but may also be a fragment of an **M_4_L_4_** tetrahedron or higher‐order architecture. In addition, as alluded to above, full consumption of the aldehyde does not necessarily indicate the formation of a MOC topology and could instead simply indicate complete conversion to the tri‐imine ligand. Therefore, the ^1^H NMR spectra of the hits were manually interpreted further to determine the reaction outcome in relation to the number of species formed and subsequently correlated with the identified topologies from the mass spectra to identify the major assemblies that had formed (Table 1). Overall, this yielded three MOCs with a *T‐*symmetric **M_4_L_4_** structure, but with different ligand and counterion components, (**Zn_4_(A1)_4_.(NTf_2_)_8_**, **Zn_4_(A1)_4_.(BF_4_)_8_** and **Zn_4_(A3)_4_.(BF_4_)_8_**, referred to as **cage 1**, **cage 2**, and **cage 3**, respectively (Figure 4). These three cages were subsequently taken forward and manually scaled‐up using the same reaction conditions for full characterization (Supporting Information S3).

**Table 1:** Hits identified from the automated analysis that were taken forward for further ^1^H NMR interpretation to identify whether a single assembly or a mixture of species had been formed as the major product(s), and if single, the structural outcome.

Precursor combination	Single or Mixed Species?	Structural Outcome
**A1/Zn(NTf_2_)_2_**	Single	**M_4_L_4_**
**A1/Zn(BF_4_)_2_**	Single	**M_4_L_4_**
**A3/Zn(BF_4_)_2_**	Single	**M_4_L_4_**
**A4/Zn(NTf_2_)_2_**	Mixed	–
**A4/Zn(OTf)_2_**	Mixed	–
**A4/Zn(BF_4_)_2_**	Mixed	–

In the case of all three scale‐ups, the tetrahedra could not be fully isolated from the minor component of the mixtures, which was assumed to be a mixture of **M_2_X_3_** helicate and/or mesocate species as observed by HRMS. By finding the ratio of the imine N = C**H** signal integrations for the tetrahedron and the **M_2_X_3_** species in each mixture, the molar composition of the tetrahedron in the mixtures could be calculated – the signal at 8.5–8.6 ppm corresponds to the 9H resonance of the helicate (assuming full conversion to **M_2_L_3_**, but could also include **M_2_I_3_** (6H), **M_2_I_2_L** (7H), and **M_2_IL_2_** (8H)) and the signal at 8.7–8.8 ppm corresponds to the 12H resonance in the tetrahedron, similar to the related structures found by Bilbeisi et al.^[^ ^23^ ^]^ For these scaled‐up samples, this gives approximate tetrahedron:helicate molar ratios of 1:0.04 in **cage 1,** 1:0.16 for **cage 2,** and 1:0.19 for **cage 3**, but it should be noted that these ratios were calculated following purification.

Diffusion ^1^H NMR measurements were acquired on the isolated samples of **cages 1–3** to verify that the assigned peaks for the **M_4_L_4_** topology correlated with a single assembly in solution (Section S3.4). Additionally, the measured diffusion of the secondary peaks present alongside **cage 2** and **cage 3** also indicated these components have a single, smaller solvodynamic radius than the major signals from the tetrahedral cages. In **cage 2**, the major species has a diffusion coefficient of 7.05 × 10^−10^ m^2^ s^−1^ while the minor species signals gave a coefficient of 8.06 × 10^−10^ m^2^ s^−1^, indicating the solvodynamic radius of the minor species is 1.14 times smaller than the tetrahedral species. Similarly in **cage 3**, the diffusion coefficients indicate that the minor species has a solvodynamic radius 1.16 times smaller (D = 7.60 × 10^−10^ m^2^ s^−1^ for the tetrahedral component and D = 8.85 × 10^−10^ m^2^ s^−1^ for the minor one). In both cases the secondary species is thus reduced in size by the same amount compared to the major cage, corresponding to the helicate impurities often observed in these systems.^[^ ^23^ ^]^ Finally, in **cage 1**, the diffusion coefficient was 1.05 × 10^−9^ m^2^ s^−1^ for the tetrahedral species, a lower value compared to **cages 2** and **3**. The discrepancy between the diffusion coefficients could be due to viscosity differences between the samples, or due to associated anions expanding the solvodynamic radii of the cationic assemblies to differing extents. While the MOC cores are analogous in structure, their solvodynamic radii cannot thus be compared directly using the Stokes–Einstein equation.^[^ ^31^ ^]^

### Effect of Metal Concentration and Precursor Stoichiometry

**Figure 5:**
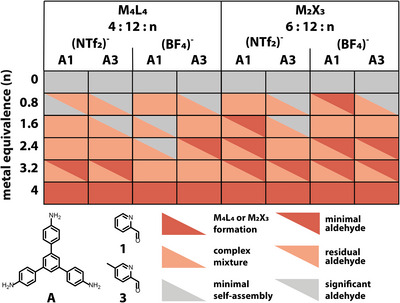
Overall results from the automated screen exploring the effect of reaction stoichiometry, ligand, and metal counterion identity and concentration. Precursor combinations A1/NTf_2_^−^ (cage 1), A1/BF_4_^−^ (cage 2), A3/NTf_2_^−^, and A3/BF_4_^−^ (cage 3) were investigated at two reaction stoichiometries, to target the M_4_L_4_ tetrahedron (4:12:n of triamine:aldehyde:metal salt) and the M_2_X_3_ helicate (6:12:n of triamine:aldehyde:metal counterion), and the metal counterion concentration was varied between 0% and 100% of 0–4 equivalence (*n* = 0, 0.8, 1.6, 2.4, 3.2, or 4). Assessment of the remaining aldehyde following the reaction categorized as either minimal (≤ 5%, red), residual (6%–20%, orange), or significant (≥ 20%, grey). The outcome of the targeted self‐assembly (M_4_L_4_ or M_2_X_3_) as either formed (red), a complex mixture (orange), or minimal (grey). A fully red box is indicative of the ideal outcome, where a reaction has minimal aldehyde remaining and a target structure has been identified.

As previously discussed, structural diversity, including variations of topicity and shape, can be achieved by changing a range of factors including the precursor components, such as the metal or counterion, and their relative stoichiometric ratios. Therefore, based on the observed formation of both tetrahedron and helicate architectures in different relative ratios in the automated screen (Tables S4–S6), and subsequent scale‐up of **cages 1–3** (**Zn_4_(A1)_4_.(NTf_2_)_8_**, **Zn_4_(A1)_4_.(BF_4_)_8_,** and **Zn_4_(A3)_4_.(BF_4_)_8_,** respectively), an additional screen was conducted using the developed automated high‐throughput workflow to explore the effect of varying the concentration of the metal salt, and also the relative precursor ratio, on targeting the **M_4_L_4_** tetrahedron versus **M_2_X_3_** helicate. This was undertaken on precursor combinations of **A1** and **A3** with counterions NTf_2_^−^ and BF_4_^−^ (Figure 5). Two different triamine:aldehyde:metal precursor ratios were investigated: 4:12:n (to favor tetrahedron formation) and 6:12:n (to favor helicate formation), alongside varying metal salt concentrations from 0% to 100% (*n* = 0–4).

For all combinations across the two stoichiometries investigated, when no metal salt was present (0%, *n* = 0), minimal self‐assembly and a high proportion of residual aldehyde was observed, indicating that the metal is required for imine formation. This is not unexpected–in the presence of water, the aldehyde‐imine equilibrium lies on the aldehyde side, with the presence of metal favoring imine formation.^[^ ^32^, ^33^ ^]^ On increasing the metal salt concentration up to 20%−40% (*n* = 0.8, 1.6), a high proportion of unreacted aldehyde was still observed alongside a complex mixture of products. As noted in the initial screen, differences in conversion were observed between **A1** and **A3**, with the latter showing more residual aldehyde due to the *meta*‐methyl group causing steric hindrance around the metal center on coordination, which reduces the likelihood of MOC formation. A counterion effect on the degree of assembly was also observed with **A3**, with significant aldehyde remaining with NTf_2_^−^ and greater conversion observed with BF_4_^−^. At increasing (60%–80%) concentrations of metal salt, while complex mixtures were typically observed in the ^1^H NMR spectra, increased starting material consumption was observed. Although the metal salt was promoting imine formation, we infer that it was not present in sufficient quantity to cleanly form the targeted assemblies. Finally, at the correct stoichiometric ratio of metal salt (100%, *n* = 4) the formation of the targeted architectures is observed, and as expected, the four combinations at 4:12:4 triamine:aldehyde:metal salt were in agreement with the initial screen, where the *T‐*symmetric **M_4_L_4_** tetrahedron was observed as the major product. However, the stoichiometric ratio of triamine:aldehyde precursors was found to play an important role in the outcome of the self‐assembly process – for the **A3/NTf_2_^−^** and **A3/BF_4_^−^** combinations carried out with a precursor ratio of 6:12:4, clean formation of the singular **M_2_I_3_** helicate (**Zn_2_(A3‐I)_3_.(BF_4_)_2_** and **Zn_2_(A3‐I)_3_.(NTf_2_)_2_**) was observed (Figures S91, S92). This contrasts with the predominantly clean formation of a tetrahedron from the same precursors when carried out in a 4:12:4 ratio. In addition, on manual inspection of the ^1^H NMR spectra for the same stoichiometric precursor ratio with **A1/NTf_2_^−^** and **A1/BF_4_^−^**, the formation of **M_2_X_3_** structures can be assumed with minimal formation of the **M_4_L_4_** tetrahedron, but one topology could not be definitively assigned – this complexity likely arises from the identity of **X** leading to a possible mix of **M_2_I_3_**_,_ **M_2_I_2_L**, **M_2_IL_2_**, and **M_2_L_3_** helicates, but also peak complexity may arise from the presence of both the meso and racemic diastereomers,^[^ ^23^, ^34^ ^]^ limiting comprehensive ^1^H NMR assignment and structure confirmation.

### Computational Modelling

**Figure 6:**
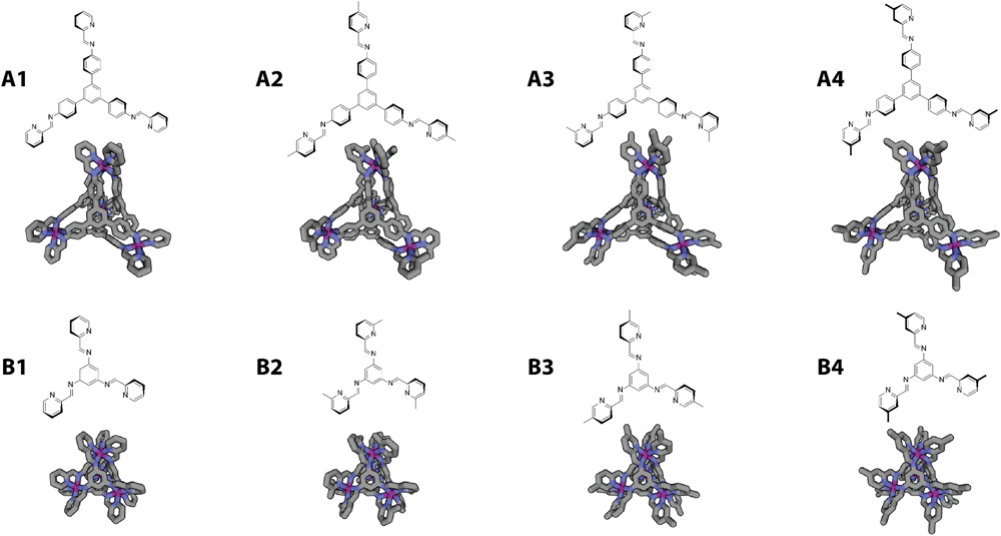
(Top) GFN2‐xTB optimized geometries of eight face‐capped M_4_L_4_ tetrahedrons assembled from Zn(II) and ligands A1‐A4, with ligand structures shown above. (Bottom) GFN2‐xTB optimized geometries of eight face‐capped M_4_L_4_ tetrahedrons assembled from Zn(II) and ligands B1–B4, with ligand structures shown above. Facial‐Λ Zn atoms are shown in purple, nitrogen atoms in blue, and carbon atoms in grey, hydrogen atoms are omitted for clarity.

To better understand the structural outcomes identified in both experimental screens using the automated analysis, computational modelling was performed on the **M_N_L_N_** (where *N* = 4 or 12) and **M_2_X_3_** (where **X** = **L** or **I**) structures, details of which are given in Supporting Information Section S5. First, the **M_4_L_4_** tetrahedra were modelled at a semiempirical level (Figure 6), with a *T‐*symmetric configuration. For both triamines **A** and **B**, the MOCs assembled with the methyl‐substituted pyridyl aldehydes **2**, **3**, and **4** are isomers of each other, and therefore the relative energies for the different tetrahedra can therefore be compared (Table 2).

**Table 2:** GFN2‐xTB semiempirical energies and r^2^SCAN‐3c//GFN2‐xTB single‐point DFT calculations for each of the methylated M_4_L_4_ tetrahedra assembled from Zn(II) with ligands A2–A4 and B2–B4. Energies are given in kJ mol^−1^ relative to tetrahedra with ligands A2 and B2 being set to zero.

Ligand	A2	A3	A4
G_GFN2‐xTB_	0	−93.7	−102.1
$G_{r^{2}\mathrm{SCAN}-3c//\mathrm{GFN}2-\mathrm{xTB}}$	0	−85.5	−95.5

Ligand	B2	B3	B4
G_GFN2‐xTB_	0	−49.3	−58.7
$G_{r^{2}\mathrm{SCAN}-3c//\mathrm{GFN}2-\mathrm{xTB}}$	0	−43.2	−53.0

Across both triamines, the same energetic trend is observed for the **M_4_L_4_** structure (**A2 **> **A3** > **A4** and **B2 **> **B3** > **B4**). The differences between **3** and **4** are relatively small but clearly suggest these are more energetically favorable than structures incorporating aldehyde **2**. This trend broadly agrees with the experimental outcomes from the automated screen discussed above, in relation to their reactivity and successful formation of a self‐assembled species. We infer that increased steric hinderance around the metal center leads to less favorable assembly outcomes due to their higher relative energies. However, while aldehyde **4** in the tetrahedral topology was the most energetically favored across the family, it typically led to the formation of a mixture of species including a **M_4_L_4_** species (Table 1), and therefore formation is not solely dependent on the energetics of the **M_4_L_4_** topology.

Because of the complexities of the **M_2_X_3_** species (where X = L or I) and the fact that helicate structures may form based on different chirality at each metal center resulting in three configurations (homochiral helicates (ΔΔ and ΛΛ), or achiral mesocates (ΔΛ)),^[^ ^35^ ^]^ further modelling of these systems was not carried out. In addition, as no *pseudo‐*icosahedra were experimentally identified, only preliminary modelling was carried out, which found that ligands formed with the smaller triamine **B**, which contains a singular aromatic ring as the core, were too small to assemble into a *pseudo‐*icosahedron.

### Automated Host–Guest Assay

Prediction of host–guest binding in MOCs is nontrivial to do reliably via computation, and curation of experimental data to validate these predictions or feed a data‐led approach remains a bottleneck.^[^ ^19^, ^20^, ^36^ ^]^ The most common approach to study and quantify host–guest binding in supramolecular assemblies is to study the change of one species’ physical property, such as a chemical resonance in NMR spectroscopy, or the absorption band in UV–vis spectroscopy.^[^ ^37^ ^]^ Typically host–guest binding studies using NMR are time consuming and expensive, requiring a large amount of material and deuterated solvents, especially for full titrations. Our aim here was therefore to develop a high‐throughput host–guest assay in a plate‐based format for MOCs. The ability to carry out many single‐point NMR binding studies at a known host–guest equivalence in parallel, performed in an automated way, allows for the rapid identification of strong binding guests, the elucidation of trends in binding behavior, and the potential identification of promising molecular separations.

For this proof‐of‐concept host–guest assay and to validate that binding studies can be translated into a high‐throughput automated protocol, the MOC (**cage 1**, **Zn_4_(A1)_4_.(NTf_2_)_8_**), concentration (0.0008 mmol mL^−1^), host–guest ratio (1:4), and range of guests (32 neutral species), were selected based on literature precedents. For example, **cage 1** has previously been reported to bind neutral guests such as ^t^BuOH and cyclohexane, which were also included in the screen as controls to validate the workflow.^[^ ^25^, ^26^ ^]^ The experiment concentration was selected where equimolar concentrations of the host and the host–guest complex would give a *K*_a_ ca. 10^3^ M⁻¹. This allows for stronger (*K*_a_ >> 10^3^ M⁻¹), intermediate (*K*_a_ ≃ 10^3^ M⁻¹) and weaker association (*K*_a_ << 10^3^ M⁻¹) to be estimated, based on previously reported ranges of values for *K*_a_ in related systems.^[^ ^25^, ^26^ ^]^ Samples were prepared in 96‐well plates using stock solutions on the Opentrons OT‐2 (500 µL of 0.0008 M host in acetonitrile‐*d_3_* and 75 µL of 0.0214 M guest stock solutions in acetonitrile) before being sealed and vortexed at room temperature (298 K), and then transferred to high‐throughput NMR tubes on the platform before being analyzed by ^1^H NMR spectroscopy after 1 and 7 days (see Supporting Information Section S6 for full details). The accuracy of automated dispensing was also validated by checking the obtained host–guest ratio via integration of free **cage 1** and unbound guest (Supporting Information Figure S102), which confirmed a 1:4 stoichiometric ratio had been achieved.

**Figure 7:**
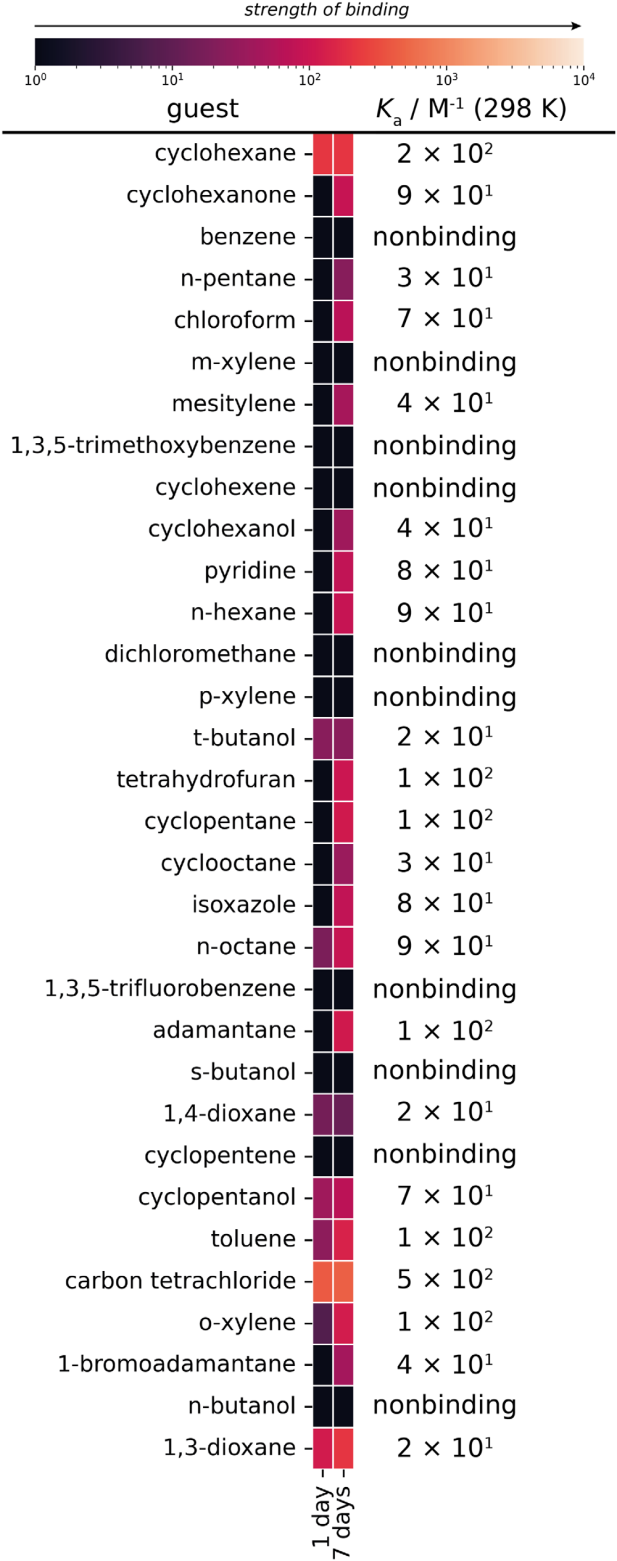
A heatmap of the calculated *K*_a_ (298 K) values from the single‐point ^1^H NMR binding assay of the host MOC, cage 1, and 32 neutral guests, for any host–guest complexes formed after 1 day and 7 days. The largest *K*_a_ value between 1 day and 7 days for each host–guest combination is shown.

From the assay, a host–guest complex was inferred to have formed if there were two sets of host peaks, one from the “empty” MOC and the other from the guest⊂MOC in slow exchange. Observation of changes in the host peak shifts were selected over changes in the guest peaks due to the aliphatic nature of many of the guests—these guests were often found in the upfield region of the ^1^H NMR spectra and therefore overlapped with solvent signals, making assignment of free and complexed guest a less viable approach in our screen. In cases where encapsulation was observed and slow‐exchange is assumed, the association constant (*K*_a_) was calculated from the integrations of host peaks relative to the host–guest peaks, assuming only a singular guest can be encapsulated (Figure 7, Supporting Information section S6). The calculated *K*_a_ values from this automated screen give a picture of the overall binding behavior in a MOC, informing a researcher of whether encapsulation has occurred and giving an indication of the relative strength of binding, which in turn can identify which combinations may be taken forward to perform full titrations on. Even from a relatively simple screen of 32 guests with one host, trends may be elucidated and differences in binding may direct a MOC for use in separations.

For **cage 1**, the strongest binding guests were carbon tetrachloride > cyclohexane > toluene > *o*‐xylene > cyclopentane, and the proportion of bound guests increased from 25% (8/32) after 1 day to 69% (22/32) after 7 days, indicating that guest uptake was slow. Separations of hydrocarbons from crude oil and alkenes from alkanes have been labelled as two of the seven chemical separations crucial to future prosperity.^[^ ^38^ ^]^ In this MOC host‐guest assay, *o*‐xylene⊂**cage 1** was observed but no encapsulation occurred for the *p*‐ or *m*‐ isomers; *t‐*butanol⊂**cage 1** was also observed but the *n*‐ and *s*‐isomers exhibited no binding, and preferential binding of an alkane over an alkene was observed with cyclohexane⊂**cage 1** versus cyclohexene, with the latter again showing no binding.

**Figure 8:**
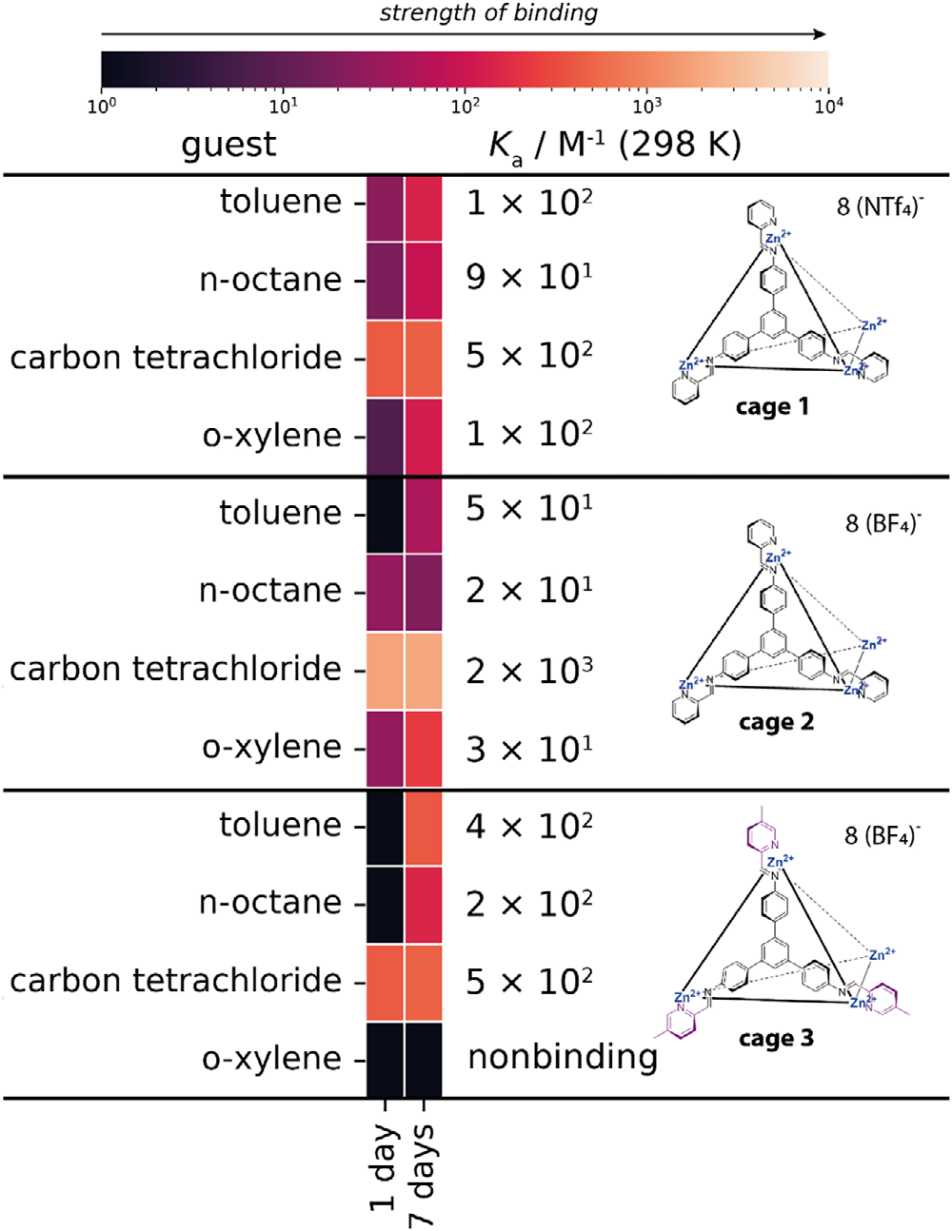
Comparison heatmaps and the calculated *K_a_* (298 K) values from the single‐point NMR binding assay of the hosts cage 1, cage 2, and cage 3 with a subset of 4 guests – toluene, *n*‐octane, carbon tetrachloride, and *o*‐xylene. The largest *K*_a_ value between 1 day and 7 days for each host–guest combination is shown.

After studying the host–guest behavior of **cage 1**, we then turned our attention to comparing the binding of a subset of guests (toluene, *n*‐octane, carbon tetrachloride, and *o*‐xylene) in the structurally analogous **cages 2** and **3** (Figure 8). **Cage 1** and **cage 2** differ only through their counterions, and **cage 2** and **cage 3** only differ based on whether the pyridyl ring is methylated or not, but computationally, the MOC cores are similar in size, suggesting **cages 1–3** have similar cavity sizes (Table S7). Therefore, this secondary host–guest screen allows us to systematically vary the cages by keeping the pyridyl substitution fixed (**cage 1** vs. **cage 2**) and then the anion fixed (**cage 2** vs. **cage 3**) to determine the impact of each of these on any changes to guest recognition and in binding.

Electrostatics can govern host–guest behavior, where a counterion that is larger than the host void space may be necessary in order to preserve guest binding capability. The bis(trifluoromethylsulfonyl)imide (triflimide, NTf_2_^−^) counterion has been previously chosen for MOCs as a non‐ or weakly‐coordinating counterion, and in the literature it was inferred to be too large to fit inside of **cage 1**.^[^ ^26^ ^]^ In contrast, the smaller size of the tetrafluoroborate (BF_4_^−^) counterion would allow possible occupation of the internal cavity, which may compete for binding with guest molecules. Nonetheless, the same general trend is observed for **cage 1** and **cage 2** where the binding was observed as follows: carbon tetrachloride > toluene > *o*‐xylene > *n*‐octane. One difference between binding was found within toluene⊂**cage 2,** where binding was slower (not observed after 1 day but was for 7 days) and weaker by an order of magnitude than for toluene⊂**cage 1**. In contrast, the association constant for carbon tetrachloride⊂**cage 2** > carbon tetrachloride⊂**cage 1**, however, this should be interpreted with care due to the single‐point derivation of these *K*_a_ values. However, for **cage 3**, the binding trends differ as follows: carbon tetrachloride > toluene > *n*‐octane, with no binding of *o‐*xylene observed. In addition, binding of toluene and *n‐*octane in **cage 3** was slower and only apparent after 7 days, although *n*‐octane had a higher preliminary *K*_a_ than for both **cage 2** and **cage 1**. This trend may indicate that methylation of the tetrahedral MOC may slow guest encapsulation, along with competitive binding from the tetrafluoroborate (BF_4_)^−^ counterion. As binding was slow, a longer time was required for the host–guest complexes to equilibrate and for encapsulation to occur.

Overall, both the full guest assay with **cage 1**, and the subset assays with **cage 2** and **cage 3**, have yielded a wealth of information into the binding behavior of this class of MOCs. This richness of data is not commonly recorded, nor is it reported in the literature. This study reveals that even subtle changes in counterion identity and ligand substitution have a measurable effect on guest‐binding properties, further contextualizing the requirement for an automated workflow in both the automated synthesis and property screening of a range of MOCs on a reasonable timescale to elucidate trends and find applications within industrial molecular separations.

## Conclusion

In conclusion, we have developed an automated low‐cost workflow for rapidly screening the self‐assembly landscape and host–guest behavior of MOCs. The use of an automated platform for screening the assembly outcomes enables the parallelization of MOC synthesis, made possible due to the reduced volume of reactants, specifically ligand, required per reaction, and the decreased human resource required in the preparation and analysis of the samples. Automation of data analysis through a free, simple, open‐source workflow created a method to rapidly assign HRMS and interpret ^1^H NMR spectra to determine the reaction outcome, which was based on a combined assessment of the reactivity by conversion and identification of the topological species, reducing experimental cost and effort. On rapid filtering of the results, identification and scale‐up of hits was then carried out. One could argue that a limitation of our workflow is that analysis was conducted on crude reaction mixtures without any purification steps, but it should be noted that self‐assembly reactions are often analyzed as crude mixtures before attempts at isolation are carried out to identify the equilibrium product, which could be perturbed on work‐up. Indeed, a change in the tetrahedron:helicate ratio was observed between crude analysis of the automated screens and after subsequent scale‐up and isolation attempts. However, due to the trade‐off between the information gained and the lack of multiple additional, expensive steps to achieve purification in an automated manner, this level of analysis was deemed acceptable. Possible extensions of our automated workflow could include a simple purification step, such as precipitation of the assembled metal‐organic complexes and filtration through filter plates, enabling analysis of both the crude and isolated materials, although handling dispersions can be challenging in an automated manner. Additionally, the integration of inline or online analytical techniques could be valuable. For example, using ReactIR for IR spectroscopy, or automated sampling for NMR spectroscopic analysis, could enable monitoring of the assembly process. However, employing these techniques may be challenging at the elevated temperatures employed in this study.

Although only a small subset of variables was screened here under a single set of reaction conditions, the extensive structural diversity of reported MOCs has been achieved through variation of parameters that include the metal ion identity, the metal counterion, topicity of the ligand, the angle and spacing between the coordinating sites of a ligand, reaction stoichiometry, solvent choice, overall concentration, the reaction temperature, and sometimes the inclusion of a guest molecule to act as a template.^[^ ^23^, ^27^ ^]^ Specifically, by tuning the reagent stoichiometries in a subsequent screen after identification of variable tetrahedron:helicate product ratios in the initial screen to identify hits, clean formation of either assembly could be achieved. This workflow could also be readily extended to include reaction condition screening and template addition in the automated screening to further study the influence on the reaction outcome for conversion, reactivity, and topological outcomes in the future. Pairing this automated experimental workflow with computational modelling allowed experimental observations to be rationalized *post facto*. Further work is needed to streamline the interconnection of these workflows to aid prediction in the future to direct synthesis.

Finally, our automated workflow was extended to include screening of host–guest binding. A general knowledge of binding was readily achieved for a MOC, and trends elucidated, which in the future, may direct further studies and applications in molecular separations. This was followed by systematic variation of the MOC, keeping the ligand functionality and then the anion fixed, to determine the impact of each of these factors on guest binding. In addition, while only a single solvent was used for the host–guest assay here, the workflow could be expanded to include different solvents, additional guests, a wider variety of MOCs, and different operational conditions. Furthermore, there is scope to expand the methods used for screening host–guest binding, for example, UV–vis measurements, as inline automated analyses or automated preparation of samples in a plate‐based format for offline analysis, could be used for determining association constants by titration. The dynamic nature of the assemblies, along with solvent and counterion effects, can drastically influence a MOC's ability to bind a guest. This limits our current ability to accurately predict binding through simulation, so screening this property in a high‐throughput approach could be used to generate a host–guest binding database which can train predictive models for host–guest pairing with a high degree of accuracy.

## Supporting Information

The authors have cited additional references within the Supporting Information.

## Conflict of Interests

The authors declare no conflict of interest.

## Supporting information



Supporting Information

## Data Availability

The data that support the findings of this study are openly available in Zenodo at https://doi.org/10.5281/zenodo.14183035, and the associated code for data curation and automated analysis is available at https://github.com/GreenawayLab/development‐automated‐workflow‐mocs.
