# Supplementary material for: Development of an Automated Workflow for Screening the Assembly and Host–Guest Behavior of Metal‐Organic Cages Towards Accelerated Discovery
Source: Angew Chem Int Ed Engl. 2025 Apr 17;64(24):e202424270. doi: 10.1002/anie.202424270 (PMC12144869; doi:10.1002/anie.202424270)
Supplement: Supplementary file 1 — Supporting Information [file ANIE-64-e202424270-s001.docx]

**Supporting Information**

**Development of an Automated Workflow for Screening the Assembly and Host-Guest Behaviour of Metal-Organic Cages towards Accelerated Discovery**

Annabel R. Basford,^a^ Aaron H. Bernardino,^a,b^ Paula C.P. Teeuwen,^b^ Benjamin D. Egleston,^a^ Joshua Humphreys,^a^ Kim E. Jelfs,^a^ Jonathan R. Nitchske,^b^ Imogen A. Riddell^c*^ and Rebecca L. Greenaway^a^*

^a^ Department of Chemistry, Molecular Sciences Research Hub, Imperial College London, White City Campus, 82 Wood Lane London W12 0BZ, UK.

^b^ Yusuf Hamied Department of Chemistry, University of Cambridge, Lensfield Road, Cambridge CB2 1EW, UK.

^c^ Department of Chemistry, University of Manchester, Oxford Road, Manchester M13 9PL, UK.

Emails: imogen.riddell@manchester.ac.uk; r.greenaway@imperial.ac.uk

Contents

[**S1. General Synthetic & Analytical Methods** 3](#_Toc193188248)

[**S2. Automated Screen for MOC Assembly** 5](#_Toc193188249)

[**S2.1 Automated Synthesis** 5](#_Toc193188250)

[**S2.2 Automated Characterisation and Analysis** 9](#_Toc193188251)

[**S2.3 Characterisation Data of Automated Screen** 11](#_Toc193188252)

[**S3. Scale up of M_4_L_4_ Metal-Organic Cages** 61](#_Toc193188253)

[**S3.1 Scale-up of Zn_4_(A1)_4_.(NTf_2_)_8_ cage 1** 61](#_Toc193188254)

[**S3.2 Scale-up of Zn_4_(A1)_4_.(BF_4_)_8_ cage 2** 65](#_Toc193188255)

[**S3.3 Scale-up of Zn_4_(A3)_4_.(BF_4_)_8_ cage 3** 69](#_Toc193188256)

[**S3.4. Diffusion NMR** 73](#_Toc193188257)

[**S4 Automated Screen for Effect of Precursor Stoichiometry and Concentration Study** 82](#_Toc193188258)

[**S4.1 Automated Synthesis** 82](#_Toc193188259)

[**S4.2.1 Automated Data Analysis** 84](#_Toc193188260)

[**S5. Computational Modelling** 93](#_Toc193188261)

[**S5.1 Tetrahedrons, M_4_L_4_** 93](#_Toc193188262)

[**S5.2 Icosahedrons, M_12_L_12_** 94](#_Toc193188263)

[**S6. Automated Guest Assay** 96](#_Toc193188264)

[**S7. References** 107](#_Toc193188265)

# **S1. General Synthetic & Analytical Methods**

**Materials:** Chemicals were purchased from TCI UK, Fluorochem, or Sigma-Aldrich. Solvents were reagent or HPLC grade and purchased from Fisher Scientific, except for acetonitrile-*d_3_* which was purchased from Sigma-Aldrich and Fluorochem. All solvents and chemicals were used as received, unless specified.

**Batch Synthesis:** All reactions were stirred magnetically using Teflon-coated stirring bars. Where heating was required, the reactions were warmed using a stirrer hotplate with heating blocks with the stated temperature being measured externally to the reaction flask with an attached probe. Removal of solvents was carried out using a rotary evaporator.

**Automated MOC Synthesis:** High-throughput experimentation and sample preparation was conducted using an Opentrons OT-2 liquid handling platform (robot v7.2.1 and 7.2.1 app version).^1^ Protocols were written using the OT-2 Python Protocol API Version 2.9, and the code required to replicate the protocol is available at (https://github.com/GreenawayLab/development-automated-workflow-mocs/tree/master/Opentrons_Protocols). The OT-2 was fitted with a single-channel GEN2 300 μL pipette and used with Opentrons OT-2 Tips, 300 μL. Both 24-well (for 8 mL vials) and 48-well (for 2 mL vials) reactor blocks were purchased from Analytical Sales, and the 6-well ANSI/SLAS microplate holder for DURAN 25 mL bottles was purchased from VWR. Reactions were conducted in capped 2 mL vials (Fisherbrand Certified Vial Kit 9mm short thread, Clear Glass, 1.5mL, PP Screw Cap, Center hole with a Silicone/PTFE septum). Micro PTFE stirrer bars (2 x 5 mm) were purchased from Scientific Laboratory Supplies and used across the high-throughput screens. Parallel solvent evaporation was conducted using an Analytical Sales EquaVAP 48-well evaporator.^2^

**FTIR:** Infrared spectra were recorded using an Agilent Technologies Cary 630 FTIR spectrometer. Spectra were acquired across a 4000-400 cm^-1^ range, with 32 background scans, a resolution of 16 and a Happ-Genzel Apodization.

**NMR Spectra:** ^1^H and ^13^C NMR spectra for precursor and scaled-up MOC characterisation were obtained using a Bruker AV400 (400 MHz/101 MHz) at 298 K spectrometer using 7”, OD 5.0 mm tubes. Automated cage characterisation samples were prepared using the Opentrons OT-2 with acetonitrile-*d_3_*, and ^1^H NMR spectra obtained with a Bruker AV400 (400 MHz) at 298 K fitted with a ‘SampleXpress’ automatic sample changer that allows both single tube submission and 96 well plate arrays of 4”, 5.0 mm outer diameter tubes in a fully automated system.^3^ The host-guest assay was conducted with 0.5 mL of MOC stock solution in acetonitrile-*d_3_* and 0.15 mL of guest stock solution in acetonitrile. Spectra were acquired using a deuterium lock in CD_3_CN (δ = 1.94 ppm) for ^1^H NMR, and CD_3_CN (δ = 1.32, 118.26 ppm) for ^13^C NMR. NMR data are presented as follows: chemical shift, peak multiplicity (s = singlet, d = doublet, t = triplet, q = quartet, qu = quintet, m = multiplet, br = broad, app = apparent), coupling constants (*J* / Hz), and integration. Chemical shifts are expressed in ppm on a δ scale relative to δ CD_3_CN (δ = 1.94 ppm).

**2D NMR:** Samples were measured at room temperature using a Bruker Avance III HD 400 MHz or Bruker DRX500 (400 MHz/126 MHz) spectrometer equipped with a gradient unit providing a maximum gradient output of 53.5 G/cm (5.35 G/cmA) and running with TopSpin3.6.5. The ^1^H COSY experiment was measured using the Bruker pulse program cosygpqf with a spectral width of 4800 Hz (centred on 5 ppm) and 512 data points. 128 experiments were collected in the indirect dimension using a 1s relaxation delay and 4 scans per experiment. The gradient pulses were sine-shaped (SINE.100) and set to 10% of maximum output. The data was processed using 512 data points in the direct dimension and 512 in the indirect dimension applying a sine bell function in both dimensions. The ^1^H-^13^C HSQC experiment was measured using the Bruker pulse program hsqcetgpsi2 with a spectral width of 6400 Hz (centred on 7 ppm) and 1024 data points in the ^1^H direct dimension and 24,000 Hz (centred on 110 ppm) in the ^13^C indirect dimension. A 1 s relaxation delay was employed and 200 experiments collected in the indirect dimension each with 4 scans. All gradient pulses were smoothed-square shaped (SMSQ10.100) and in the ratio of 80%:20.1%:11%:-5% of maximum output. The data was processed using 1024 data points and cosine squared functions in both dimensions

**HRMS (ESI+):** High-resolution mass spectrometry (HRMS) was obtained on a Waters Xevo TQD machine and a Waters SYNAPT G2-Si LC, infused from a Harvard Syringe Pump at a rate of 10 μL per minute.

# **S2. Automated Screen for MOC Assembly**

### **S2.1 Automated Synthesis**

**
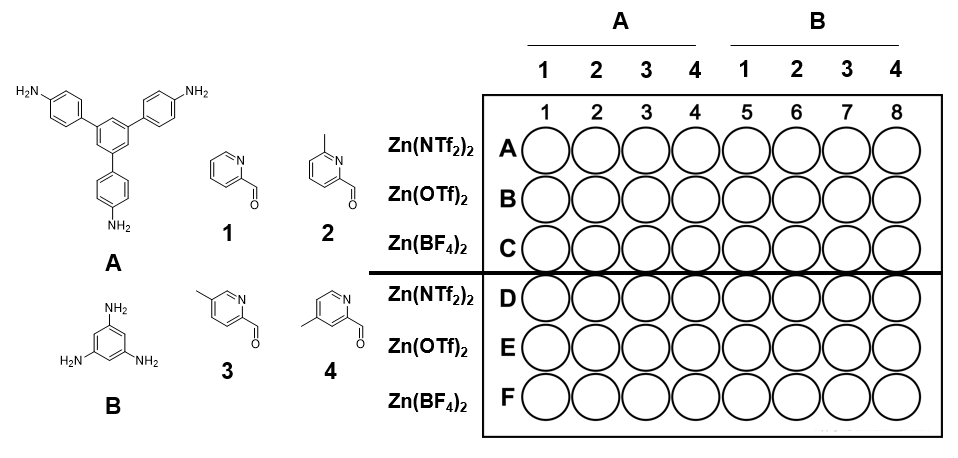
**

**Figure S1:** Plate format for automated screening of metal-organic cages. Triamines **A** and **B** were screened against four aldehydes (**1-4**) and three metal counter ions – Zn(NTf_2_)_2_, Zn(OTf)_2_ and Zn(BF_4_)_2_. This was repeated twice to check reproducibility, totalling 48 reactions.

**Automated synthesis screen general method: Zn_4_(A1)_4_.(NTf_2_)_8_** (**cage 1**) was selected from literature and the reaction conditions adapted to total the same overall precursor concentration of our previous automated study.^4,5^ Automated experimental screening was conducted using the Opentrons OT-2 liquid handling platform (robot v7.2.1 and 7.2.1 app version) with settings based on the optimisation and calibration of the system with the bulk reaction solvent used for screening (in this case, acetonitrile, Table S1 and Figure S2) – gantry speed of 250 mm s^-1^, and plunger flow rates (for aspirating and dispensing) of 70 µL s^-1^ using the GEN2 300 µL Opentrons proprietary pipette. Before transferring a new substance stock solution, 100 µL of the solution was aspirated into the pipette which was then lifted to the top of the vial, followed by a 10 s delay, and dispensed back into the same vial. This was done to pre-saturate and allow any swelling of the pipette tip, which was found to reduce the dripping during transfer across the deck. A maximum volume of 250 µL of a stock solution was transferred at a time, using a 15 µL air gap. Where more than 250 µL of a substance was required, multiple transfers were made. Protocols were written using OT-2 Python Protocol API Version 2.9 and simulated prior to conducting each run using the Opentrons Python package - the code required to replicate the protocol is available at https://github.com/GreenawayLab/development-automated-workflow-mocs/tree/master/Opentrons_Protocols. Following these settings, the OT-2 transferred volumes of the triamine stock solutions, followed by volumes of the aldehyde stock solutions, then the Zn(II) salt stock solutions, from a 24-well plate and topped up to a total volume of 1 mL with acetonitrile from a 6-well solvent plate, stock solution concentrations, reaction ratios and stock solution volumes are found in Tables S2 and S3. Reactions were stirred at 70 °C for 17 hours. The reaction samples were allowed to cool to room temperature, where a 20 μL aliquot was taken and dissolved in 1 mL MeCN for analysis using high-resolution mass spectrometry. The bulk reaction samples were then then placed in a 48-well EquaVAP parallel solvent evaporator before being returned to the OT-2 deck and redissolved in 700 μL acetonitrile-d_3_, 600 μL of this solution was then transferred into a 3D printed 96-well NMR tube holder plate (https://github.com/GreenawayLab/StreamliningAutomated-Discovery-POCs/tree/main/3D-printing) and ^1^H NMR analysis was undertaken.

**Table S1:** Calibration 1 of Opentrons OT-2 with acetonitrile across volumes 20 – 280 μL as to not exceed the maximum volume of the pipette when considering a 15 μL airgap. Dispenses were carried out twice per targeted volume and averaged. Gantry speeds were set to 250 mm s^-1^ for the X, Y and Z axes. The 300 μL pipette aspiration and dispense flowrate was set to 70 μL s^-1^ and transfers included a pre-saturation step (100 μL aspiration, 10 s delay, 100 μL dispense back into the source vial) to prevent dripping and allow accurate transfers. Vials were pre-weighed before the dispense and after, with the actual dispensed volume calculated using the density of acetonitrile and the accuracy against the target volume calculated.

| **Target Dispense Volume / μL** | **Average Dispensed Volume / μL** | **Δvol / μL** | **Standard deviation** |
| --- | --- | --- | --- |
| 20 | 9 | -12 | 1.97 |
| 40 | 28 | -12 | 2.16 |
| 60 | 47 | -13 | 0.95 |
| 80 | 68 | -12 | 1.21 |
| 100 | 89 | -11 | 1.40 |
| 120 | 108 | -12 | 3.82 |
| 140 | 130 | -10 | 1.27 |
| 160 | 148 | -12 | 1.46 |
| 180 | 169 | -11 | 1.46 |
| 200 | 189 | -11 | 3.69 |
| 210 | 199 | -11 | 2.86 |
| 220 | 210 | -10 | 1.91 |
| 230 | 221 | -9 | 0.06 |
| 240 | 232 | -8 | 4.64 |
| 250 | 239 | -11 | 0.89 |
| 260 | 251 | -9 | 0.13 |
| 270 | 259 | -11 | 0.70 |
| 280 | 274 | -6 | 2.48 |

**
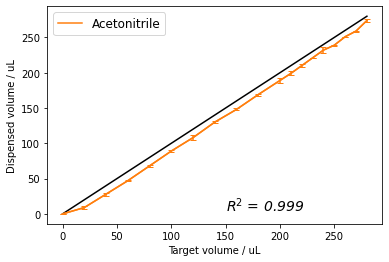
**

**Figure S2:** Calibration 1 of the Opentrons OT-2 with acetonitrile (orange) compared to the targeted dispense volumes (black). Dispenses were measured up to 280 μL and averaged with error bars included showing the standard deviation.

**Table S2:** Precursor stock solutions in acetonitrile for the automated screening.

| **Precursor** | **Molecular Weight / g mol^-1^** | **Stock Solution concentration / mg mL^-1^** | **Stock Solution concentration / mmol mL^-1^** |
| --- | --- | --- | --- |
| **A** | 351.45 | 5 | 0.0142 |
| **B** | 123.14 | 5 | 0.0406 |
| **1** | 107.11 | 5 | 0.0467 |
| **2** | 121.14 | 5 | 0.0413 |
| **3** | 121.14 | 5 | 0.0413 |
| **4** | 121.14 | 5 | 0.0413 |
| **Zn(NTf_2_)_2_** | 625.68 | 5 | 0.0080 |
| **Zn(OTf)_2_** | 363.53 | 5 | 0.0138 |
| **Zn(BF_4_)_2_.6H_2_O** | 365.09 | 5 | 0.0137 |

**Table S3:** Stock solution and solvent volumes used in each reaction on the Opentrons OT-2 platform for the automated MOC synthesis. The ratio of triamine:aldehyde:counterion was 4:12:4, 0.0019:0.0057:0.0019 totalling 0.0095 mmol in 1 mL.

| **Vial** | **Triamine** | **Amount of triamine (mmol)** | **Volume triamine stock solution (mL)** | **Aldehyde** | **Amount of aldehyde (mmol)** | **Volume of aldehyde (mL)** | **Counter ion** | **Amount of counter ion (mmol)** | **Volume of counter ion (mL)** | **Volume of CH_3_CN**  **top-up (total = 1 mL)** |
| --- | --- | --- | --- | --- | --- | --- | --- | --- | --- | --- |
| A1 | **A** | 0.0019 | 0.134 | **1** | 0.0057 | 0.122 | **Zn(NTf_2_)_2_** | 0.0019 | 0.238 | 0.507 |
| A2 | **A** | 0.0019 | 0.134 | **2** | 0.0057 | 0.138 | **Zn(NTf_2_)_2_** | 0.0019 | 0.238 | 0.491 |
| A3 | **A** | 0.0019 | 0.134 | **3** | 0.0057 | 0.138 | **Zn(NTf_2_)_2_** | 0.0019 | 0.238 | 0.491 |
| A4 | **A** | 0.0019 | 0.134 | **4** | 0.0057 | 0.138 | **Zn(NTf_2_)_2_** | 0.0019 | 0.238 | 0.491 |
| A5 | **B** | 0.0019 | 0.047 | **1** | 0.0057 | 0.122 | **Zn(NTf_2_)_2_** | 0.0019 | 0.238 | 0.593 |
| A6 | **B** | 0.0019 | 0.047 | **2** | 0.0057 | 0.138 | **Zn(NTf_2_)_2_** | 0.0019 | 0.238 | 0.577 |
| A7 | **B** | 0.0019 | 0.047 | **3** | 0.0057 | 0.138 | **Zn(NTf_2_)_2_** | 0.0019 | 0.238 | 0.577 |
| A8 | **B** | 0.0019 | 0.047 | **4** | 0.0057 | 0.138 | **Zn(NTf_2_)_2_** | 0.0019 | 0.238 | 0.577 |
| B1 | **A** | 0.0019 | 0.134 | **1** | 0.0057 | 0.122 | **Zn(OTf)_2_** | 0.0019 | 0.138 | 0.606 |
| B2 | **A** | 0.0019 | 0.134 | **2** | 0.0057 | 0.138 | **Zn(OTf)_2_** | 0.0019 | 0.138 | 0.590 |
| B3 | **A** | 0.0019 | 0.134 | **3** | 0.0057 | 0.138 | **Zn(OTf)_2_** | 0.0019 | 0.138 | 0.590 |
| B4 | **A** | 0.0019 | 0.134 | **4** | 0.0057 | 0.138 | **Zn(OTf)_2_** | 0.0019 | 0.138 | 0.590 |
| B5 | **B** | 0.0019 | 0.047 | **1** | 0.0057 | 0.122 | **Zn(OTf)_2_** | 0.0019 | 0.138 | 0.693 |
| B6 | **B** | 0.0019 | 0.047 | **2** | 0.0057 | 0.138 | **Zn(OTf)_2_** | 0.0019 | 0.138 | 0.677 |
| B7 | **B** | 0.0019 | 0.047 | **3** | 0.0057 | 0.138 | **Zn(OTf)_2_** | 0.0019 | 0.138 | 0.677 |
| B8 | **B** | 0.0019 | 0.047 | **4** | 0.0057 | 0.138 | **Zn(OTf)_2_** | 0.0019 | 0.138 | 0.677 |
| C1 | **A** | 0.0019 | 0.134 | **1** | 0.0057 | 0.122 | **Zn(BF_4_)_2_** | 0.0019 | 0.139 | 0.606 |
| C2 | **A** | 0.0019 | 0.134 | **2** | 0.0057 | 0.138 | **Zn(BF_4_)_2_** | 0.0019 | 0.139 | 0.590 |
| C3 | **A** | 0.0019 | 0.134 | **3** | 0.0057 | 0.138 | **Zn(BF_4_)_2_** | 0.0019 | 0.139 | 0.590 |
| C4 | **A** | 0.0019 | 0.134 | **4** | 0.0057 | 0.138 | **Zn(BF_4_)_2_** | 0.0019 | 0.139 | 0.590 |
| C5 | **B** | 0.0019 | 0.047 | **1** | 0.0057 | 0.122 | **Zn(BF_4_)_2_** | 0.0019 | 0.139 | 0.692 |
| C6 | **B** | 0.0019 | 0.047 | **2** | 0.0057 | 0.138 | **Zn(BF_4_)_2_** | 0.0019 | 0.139 | 0.676 |
| C7 | **B** | 0.0019 | 0.134 | **3** | 0.0057 | 0.138 | **Zn(BF_4_)_2_** | 0.0019 | 0.139 | 0.676 |
| C8 | **B** | 0.0019 | 0.134 | **4** | 0.0057 | 0.138 | **Zn(BF_4_)_2_** | 0.0019 | 0.139 | 0.676 |
| D1 | **A** | 0.0019 | 0.134 | **1** | 0.0057 | 0.122 | **Zn(NTf_2_)_2_** | 0.0019 | 0.238 | 0.507 |
| D2 | **A** | 0.0019 | 0.134 | **2** | 0.0057 | 0.138 | **Zn(NTf_2_)_2_** | 0.0019 | 0.238 | 0.491 |
| D3 | **A** | 0.0019 | 0.134 | **3** | 0.0057 | 0.138 | **Zn(NTf_2_)_2_** | 0.0019 | 0.238 | 0.491 |
| D4 | **A** | 0.0019 | 0.134 | **4** | 0.0057 | 0.138 | **Zn(NTf_2_)_2_** | 0.0019 | 0.238 | 0.491 |
| D5 | **B** | 0.0019 | 0.047 | **1** | 0.0057 | 0.122 | **Zn(NTf_2_)_2_** | 0.0019 | 0.238 | 0.593 |
| D6 | **B** | 0.0019 | 0.047 | **2** | 0.0057 | 0.138 | **Zn(NTf_2_)_2_** | 0.0019 | 0.238 | 0.577 |
| D7 | **B** | 0.0019 | 0.134 | **3** | 0.0057 | 0.138 | **Zn(NTf_2_)_2_** | 0.0019 | 0.238 | 0.577 |
| D8 | **B** | 0.0019 | 0.134 | **4** | 0.0057 | 0.138 | **Zn(NTf_2_)_2_** | 0.0019 | 0.238 | 0.577 |
| E1 | **A** | 0.0019 | 0.134 | **1** | 0.0057 | 0.122 | **Zn(OTf)_2_** | 0.0019 | 0.138 | 0.606 |
| E2 | **A** | 0.0019 | 0.134 | **2** | 0.0057 | 0.138 | **Zn(OTf)_2_** | 0.0019 | 0.138 | 0.590 |
| E3 | **A** | 0.0019 | 0.134 | **3** | 0.0057 | 0.138 | **Zn(OTf)_2_** | 0.0019 | 0.138 | 0.590 |
| E4 | **A** | 0.0019 | 0.134 | **4** | 0.0057 | 0.138 | **Zn(OTf)_2_** | 0.0019 | 0.138 | 0.590 |
| E5 | **B** | 0.0019 | 0.047 | **1** | 0.0057 | 0.122 | **Zn(OTf)_2_** | 0.0019 | 0.138 | 0.693 |
| E6 | **B** | 0.0019 | 0.047 | **2** | 0.0057 | 0.138 | **Zn(OTf)_2_** | 0.0019 | 0.138 | 0.677 |
| E7 | **B** | 0.0019 | 0.134 | **3** | 0.0057 | 0.138 | **Zn(OTf)_2_** | 0.0019 | 0.138 | 0.677 |
| E8 | **B** | 0.0019 | 0.134 | **4** | 0.0057 | 0.138 | **Zn(OTf)_2_** | 0.0019 | 0.138 | 0.677 |
| F1 | **A** | 0.0019 | 0.134 | **1** | 0.0057 | 0.122 | **Zn(BF_4_)_2_** | 0.0019 | 0.139 | 0.606 |
| F2 | **A** | 0.0019 | 0.134 | **2** | 0.0057 | 0.138 | **Zn(BF_4_)_2_** | 0.0019 | 0.139 | 0.590 |
| F3 | **A** | 0.0019 | 0.134 | **3** | 0.0057 | 0.138 | **Zn(BF_4_)_2_** | 0.0019 | 0.139 | 0.590 |
| F4 | **A** | 0.0019 | 0.134 | **4** | 0.0057 | 0.138 | **Zn(BF_4_)_2_** | 0.0019 | 0.139 | 0.590 |
| F5 | **B** | 0.0019 | 0.047 | **1** | 0.0057 | 0.122 | **Zn(BF_4_)_2_** | 0.0019 | 0.139 | 0.692 |
| F6 | **B** | 0.0019 | 0.047 | **2** | 0.0057 | 0.138 | **Zn(BF_4_)_2_** | 0.0019 | 0.139 | 0.676 |
| F6 | **B** | 0.0019 | 0.134 | **3** | 0.0057 | 0.138 | **Zn(BF_4_)_2_** | 0.0019 | 0.139 | 0.676 |
| F8 | **B** | 0.0019 | 0.134 | **4** | 0.0057 | 0.138 | **Zn(BF_4_)_2_** | 0.0019 | 0.139 | 0.676 |

### **S2.2 Automated Characterisation and Analysis**

**S2.2.1 Sample preparation**

Following the reaction times, a 20 μL sample was taken for HRMS analysis, where it was diluted with acetonitrile and filtered through glass fibre (Sartorius MG 160 Grade White Quartz Microfiber Filters).^6^ The remaining reaction mixture solvent was removed using an EquaVAP,^2^ before the samples were redissolved in 600 μL acetonitrile-*d_3_* and ^1^H NMR analysis undertaken.

**S2.2.2 Automated Data Analysis**

**moc_ms_analyser.py**

The automated analysis of the raw HRMS data was carried out using the Python script *moc_ms_analyser.py*, extended from source code of our cage database tool package – *cagey* – which was originally written for analysis of porous organic cages.^5,7^ To streamline analysis of the HRMS for analysis of metal-organic cages, the script was adapted to calculate a wider range of masses for combinations of the reaction precursors expected to form in the reaction screen. These were based on the possible connectivity between six-coordinate metal ions, tri-topic ligands and it’s corresponding di-topic intermediates (where the triamine has undergone two imine condensation reactions with a given aldehyde while a free amine remains), and with a varying number of counterions. For each reaction, the metal, triamine, aldehyde and counterion SMILES were loaded as a dictionary and the empirical formulae were calculated for the metal ion, tri-topic ligand, di-topic intermediate, and counterion. These were based on the following rules for stoichiometry of the assemblies based on the minimum connectivity to form a linear oligomer (Equation S1) and the maximum saturation of the metal sites (Equation 2), where N_Zn_, N_Tri_, and N_Di_ are the integer counts of the Zn(II) metal, tri-topic ligand and di-topic intermediate respectively:

$N_{\mathrm{Tri}} + N_{\mathrm{Di}} +1 \leq N_{\mathrm{Zn}}$ (S1)

$N_{\mathrm{Zn}}\leq2N_{\mathrm{Tri}}+ N_{\mathrm{Di}}+1$ (S2)

The number of possible structures predicted by these equations was verified manually for N_Tri_ + N_Di_ ≤ 3 by sketching the possible connectivity of the Zn(II) metal and the tri-topic ligand and di-topic intermediate. The possible number of counterions (N_Cnt_) for each assembly was an integer 0 ≤ N_Cnt_ ≤ 2N_Zn._ This gave a total of 257 combinations with N_Zn_ ≤ 7, matching the value calculated by the analyser using the equations. The allowed combinations of these components were then calculated for N_Tri_ + N_Di_ ≤ 12, with their corresponding empirical formulae, charges and the isotope splitting patterns, using the Python package *pyOpenMS,*^8^ and were combined into a dictionary. The experimental HRMS data was then searched for a peak of the predicted m/z isotope splitting for each of the calculated possible solutions. If a peak was found within a tolerance of 5 ppm of the predicted m/z peak, the structure was detected and then written to a dataframe containing: the formula of the structure, found m/z, found intensity, predicted m/z, predicted intensity and predicted charge. The dataframe was then ordered by formula and the m/z splitting between the peaks calculated to assign the charge. The results were then filtered to formulae found where the observed charge matched the predicted charge, yielding a machine readable dataframe of the HRMS results. All HRMS analysis scripts can be found on the GitHub page (<https://github.com/GreenawayLab/development-automated-workflow-mocs/tree/master/ms>).

**moc­_nmr_analyser.py**

The automated analysis of the raw ^1^H NMR data was carried out using the Python script *moc_nmr_analyser.py*, adapted from source code of our cage database tool package – *cagey* – which was found to have 98% accuracy between automated and manual analysis.^5,7^ Firstly, the raw ^1^H NMR spectra were subjected to an initial standardised pre-processing procedure which involved baseline correction, phase correction, and Fourier transformation in the proprietary Bruker TopSpin software. The pre-processed spectra of all reactions were then exported to a singular folder and the path/to/folder/ given as the singular argument to the Python script. The script utilises *nmrglue*, an open-source package for processing, manipulating, and analysing NMR spectra within Python, and provides several utility functions for reading Bruker NMR files, peak picking, and integrating NMR data. Using this software, aldehyde peaks were identified by performing peak picking in the chemical shift region of 9.0-11.0 ppm at a threshold of 10000 a.u. using the peak picking function within the *nmrglue* package, using the ‘connected’ algorithm. The ‘connected’ algorithm identifies all nodes (peaks) that are above a certain threshold, iteratively determining whether each of those nodes are separated from one another, defining each separate node as an individual peak. Imine peaks and other peaks within the aromatic region were detected within the range of 6.5-9.0 ppm, using the same peak picking algorithm and threshold. The range of 9-11 ppm was selected for detecting the aldehyde peak to accommodate the additional deshielding effects of the aromatic ring in close proximity to the aldehyde proton, present in many of the trialdehyde precursors. The range of 6.5-9.0 ppm was selected for the imine peaks due to their known occurrence across a wide range of shifts influenced by external factors that affect the proton environment, such as hydrogen bonding. To avoid detecting false positive peaks from the NMR solvent acetonitrile, the peak corresponding to this solvent at 1.96 ppm and its satellites were filtered after peak picking. Analysis was undertaken to assess for conversion, where any aldehyde peak was identified would indicate incomplete conversion. The percentage intensity relative to the largest imine peak was calculated and categorized into three outcomes – (i) aldehyde is below 1% and categorized as minor, (ii) aldehyde is between 1-5% and can still be considered for scale-up, and (iii) aldehyde is above 5% and conversion is not satisfactory. All ^1^H NMR automated analysis scripts can be found on the GitHub page (https://github.com/GreenawayLab/development-automated-workflow-mocs/tree/master/nmr).

### **S2.3 Characterisation Data of Automated Screen**

Following the automated synthetic screen and analysis of 48 reactions, (24 combinations, each repeated once), six combinations were identified to yield the optimal outcome. This outcome found the targeted **M_N_L_N_** or **M_2_X_3_** (X = L or I) as major peaks in the HRMS spectrum and identified the residual aldehyde peak from unreacted aldehyde precursor to be <1%, relative to the largest identified cage peak found in the imine region. In the case of these six ‘hits’, full assignment of the ^1^H NMR has been attempted in both the reaction and the repeat to identify firstly, whether a single or a mixture of species formed, and then if a single topology, whether a targeted MOC topology could be assigned. For all 24 precursor combinations, spectra have been included but not all have been fully assigned. All HRMS and ^1^H NMR raw data can be found on Zenodo at https://doi.org/10.5281/zenodo.14183035.


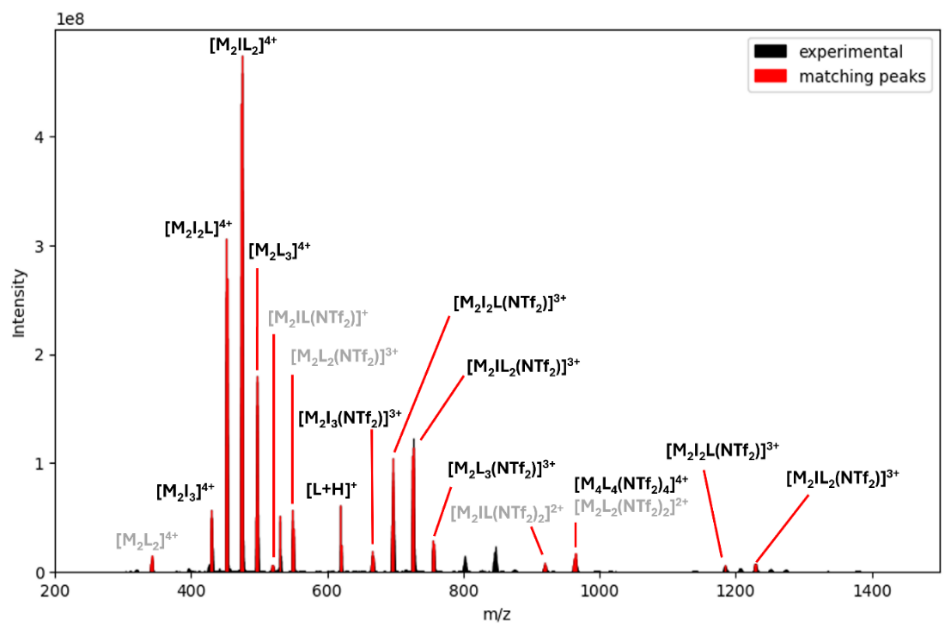

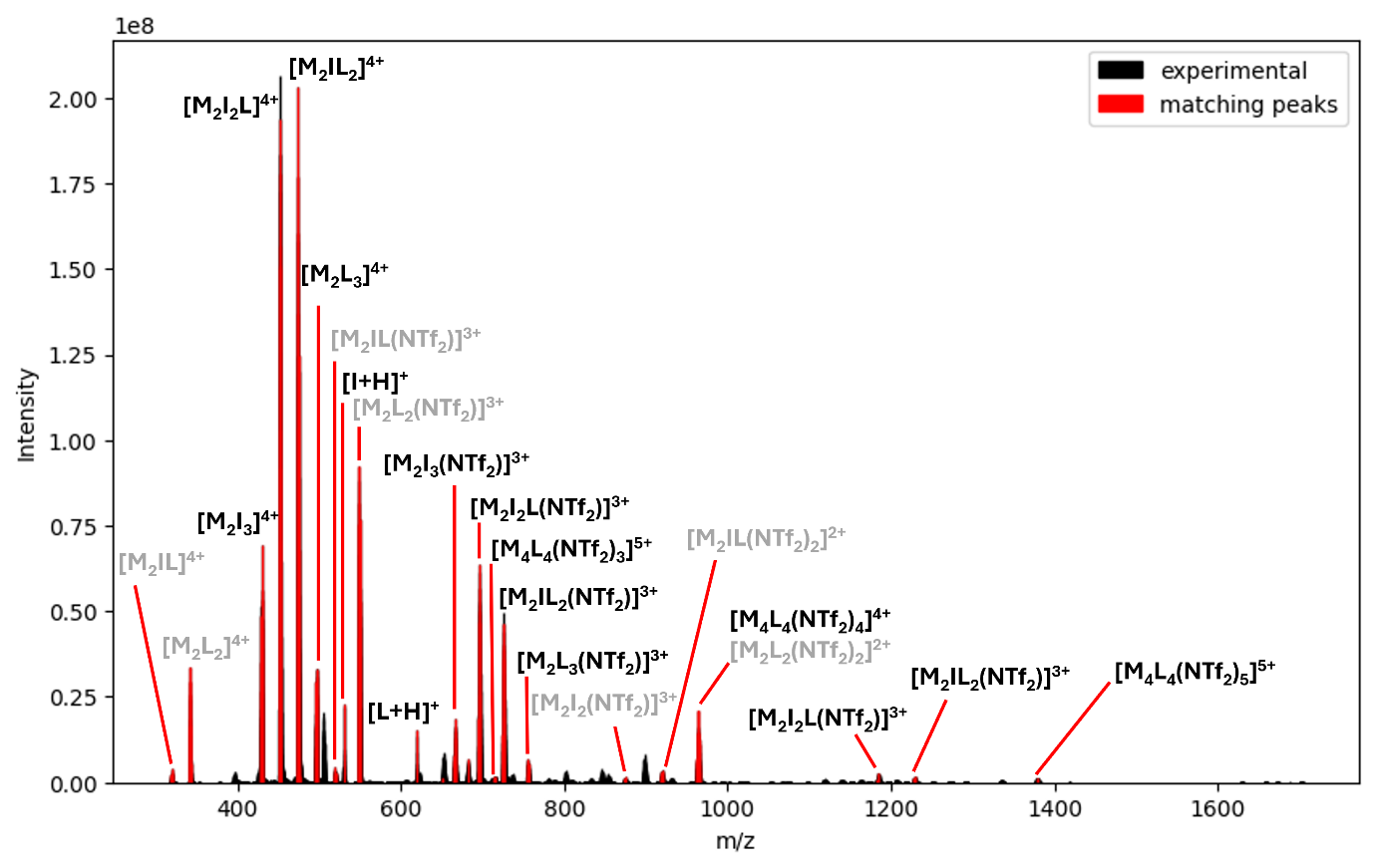


**Figure S3:** HRMS spectrum of the reaction between triamine **A**, aldehyde **1** and metal counter ion Zn(NTf_2_)_2_  for the first (top) and second (bottom) repeat screen. HRMS data are in black and the matching peaks from the automated HRMS analysis are identified in red. Peaks of targeted **M_N_L_N_** or **M_2_X_3_** where X = L or I are labelled in black and fragments or intermediates are labelled in grey.

**a**

**a**

**b**

**b,d**

**c**

**c**

**d**

**e**

**e**

**f**

**g**

**h**

**h**

**f**

**g**

**a**

**b,d**

**c**

**e**

**h**

**f**

**g**

**Figure S4:** ^1^H NMR (CD_3_CN) spectrum of the reaction between triamine **A**, aldehyde **1** and metal counter ion Zn(NTf_2_)_2_  for the first (top) and the second (bottom) repeat screen.


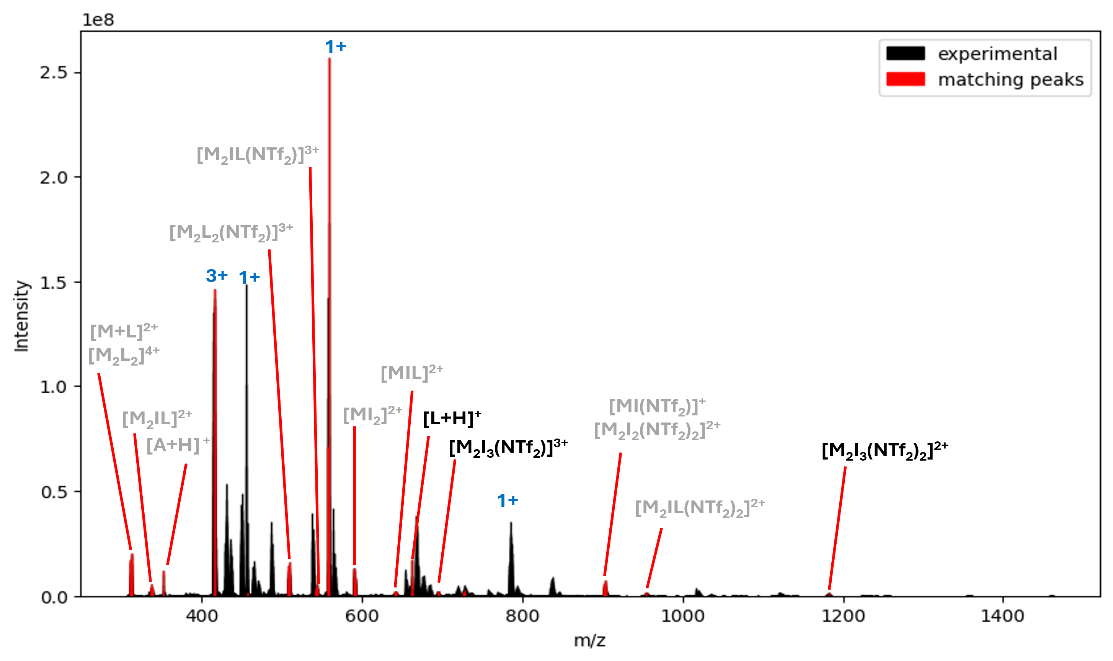

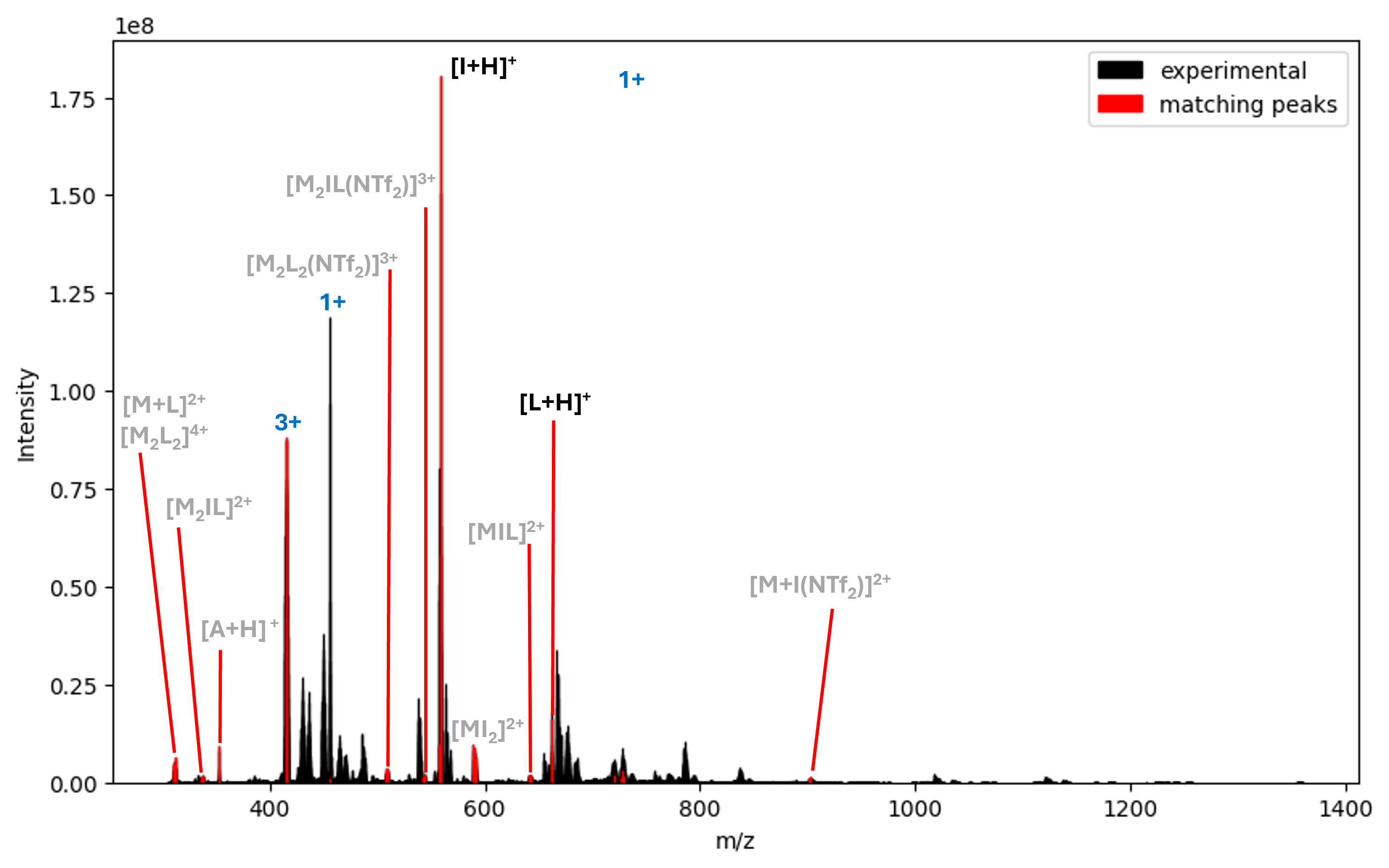


**Figure S5:** HRMS spectrum of the reaction between triamine **A**, aldehyde **2** and metal counter ion Zn(NTf_2_)_2_ for the first (top) and the second (bottom) repeat screen. HRMS data in black and the matching peaks from the automated HRMS analysis are identified in red. Peaks of targeted **M_N_L_N_** or **M_2_X_3_** where X = L or I are labelled (black) and fragments or intermediates are labelled in grey. Charges of major peaks in HRMS spectrum that were not identified are labelled in blue.

**Figure S6:** ^1^H NMR (CD_3_CN) spectrum of the reaction between triamine **A**, aldehyde **2** and metal counter ion Zn(NTf_2_)_2_  for the first (top) and the second (bottom) repeat screen.


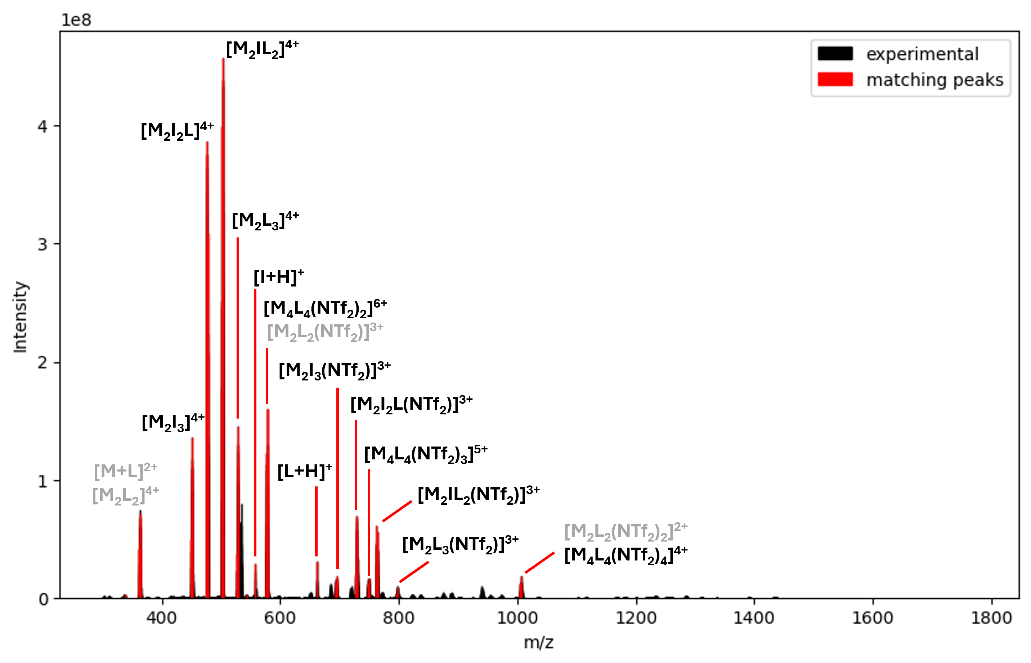

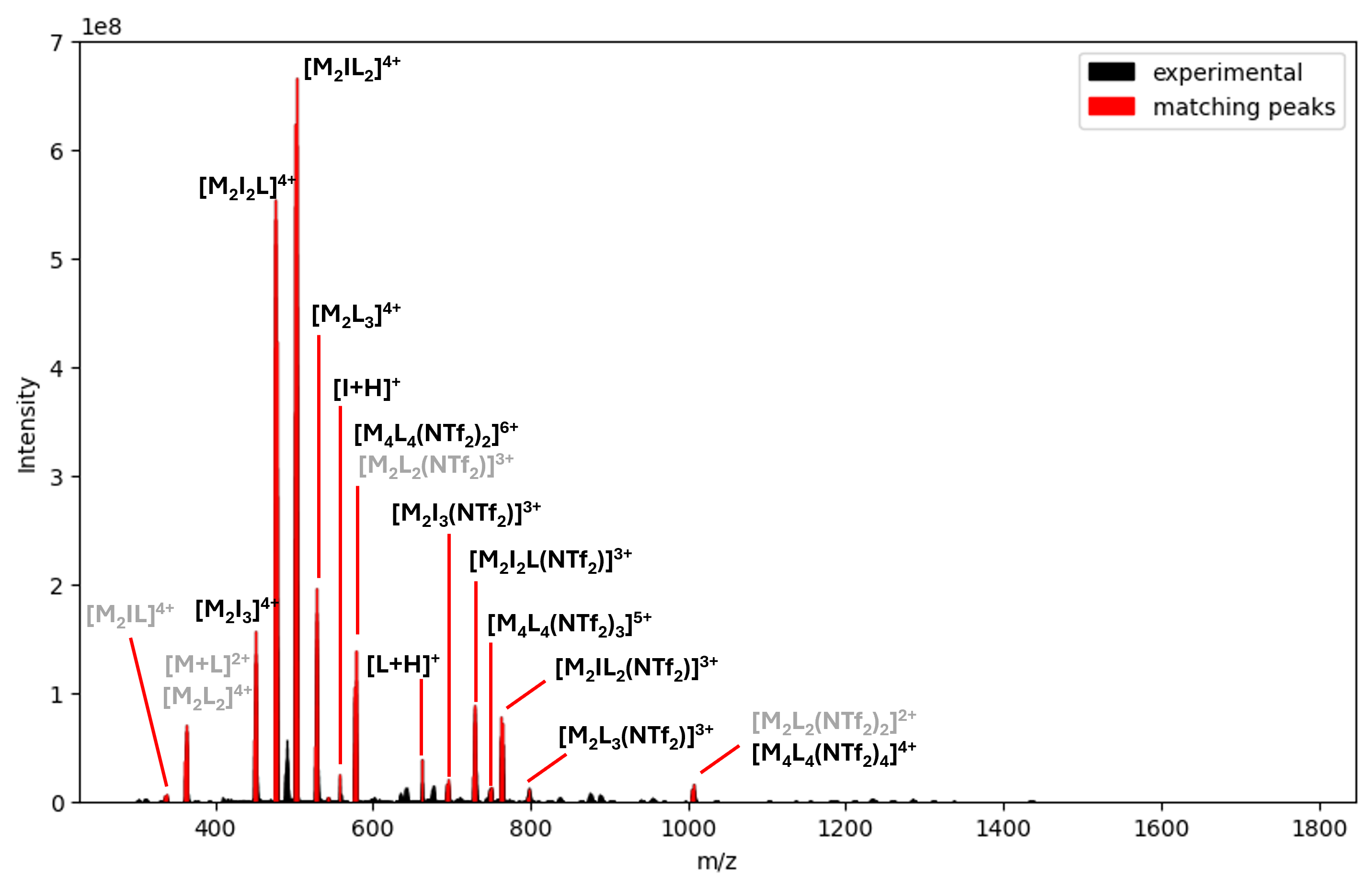


**Figure S7:** HRMS spectrum of the reaction between triamine **A**, aldehyde **3** and metal counter ion Zn(NTf_2_)_2_ for the first (top) and the second (bottom) repeat screen HRMS data in black and the matching peaks from the automated HRMS analysis are identified in red. Peaks of targeted **M_N_L_N_** or **M_2_X_3_** where X = L or I are labelled in black and fragments or intermediates are labelled in grey.

**Figure S8:** ^1^H NMR (CD_3_CN) spectrum of the reaction between triamine **A**, aldehyde **3** and metal counter ion Zn(NTf_2_)_2_ for the first (top) and the second (bottom) repeat screen.


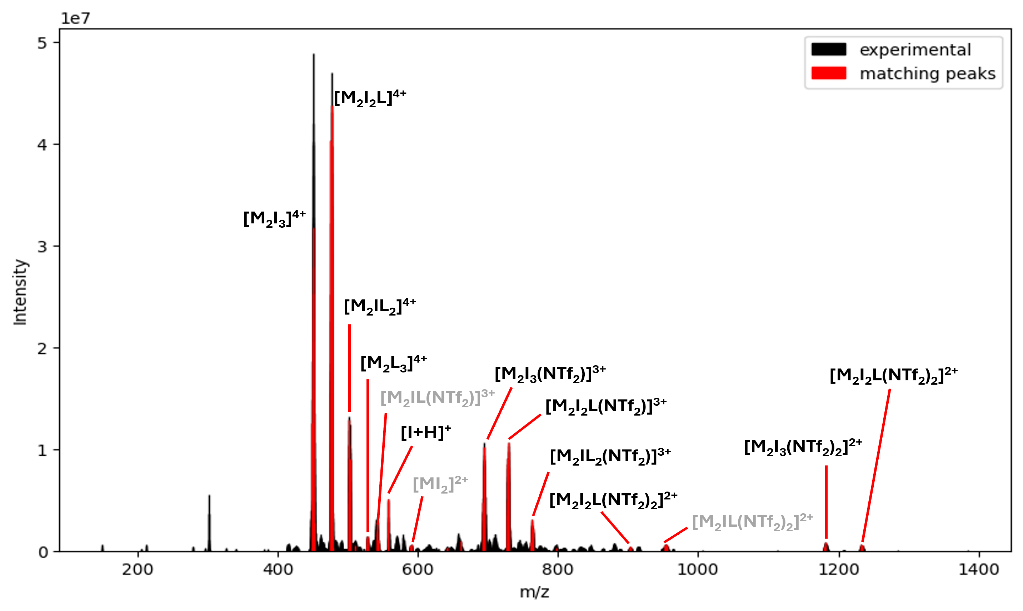

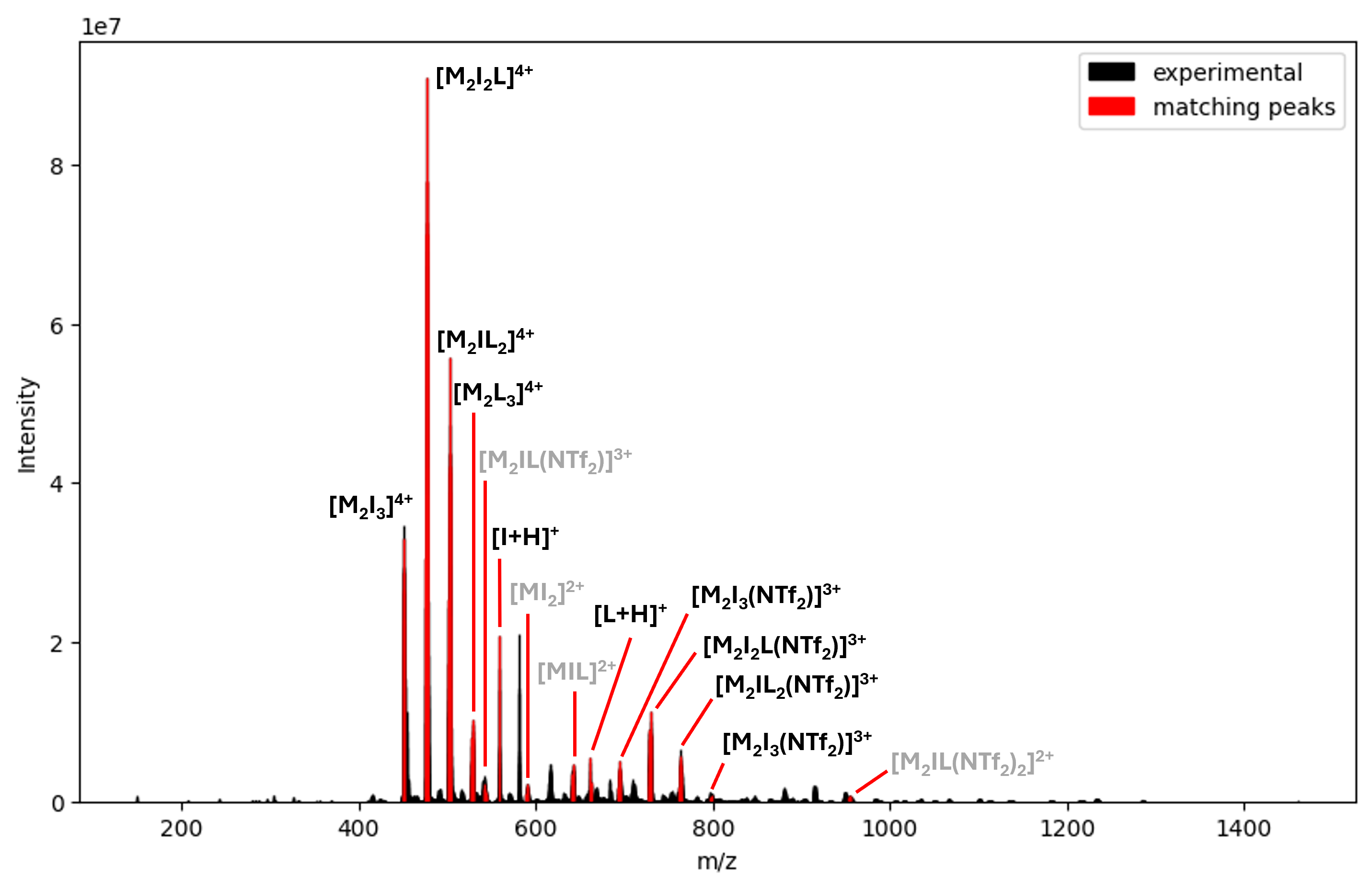


**Figure S9:** HRMS spectrum of the reaction between triamine **A**, aldehyde **4** and metal counter ion Zn(NTf_2_)_2_ for the first (top) and the second (bottom) repeat screen HRMS data in black and the matching peaks from the automated HRMS analysis are identified in red. Peaks of targeted **M_N_L_N_** or **M_2_X_3_** where X = L or I are labelled in black and fragments or intermediates are labelled in grey.


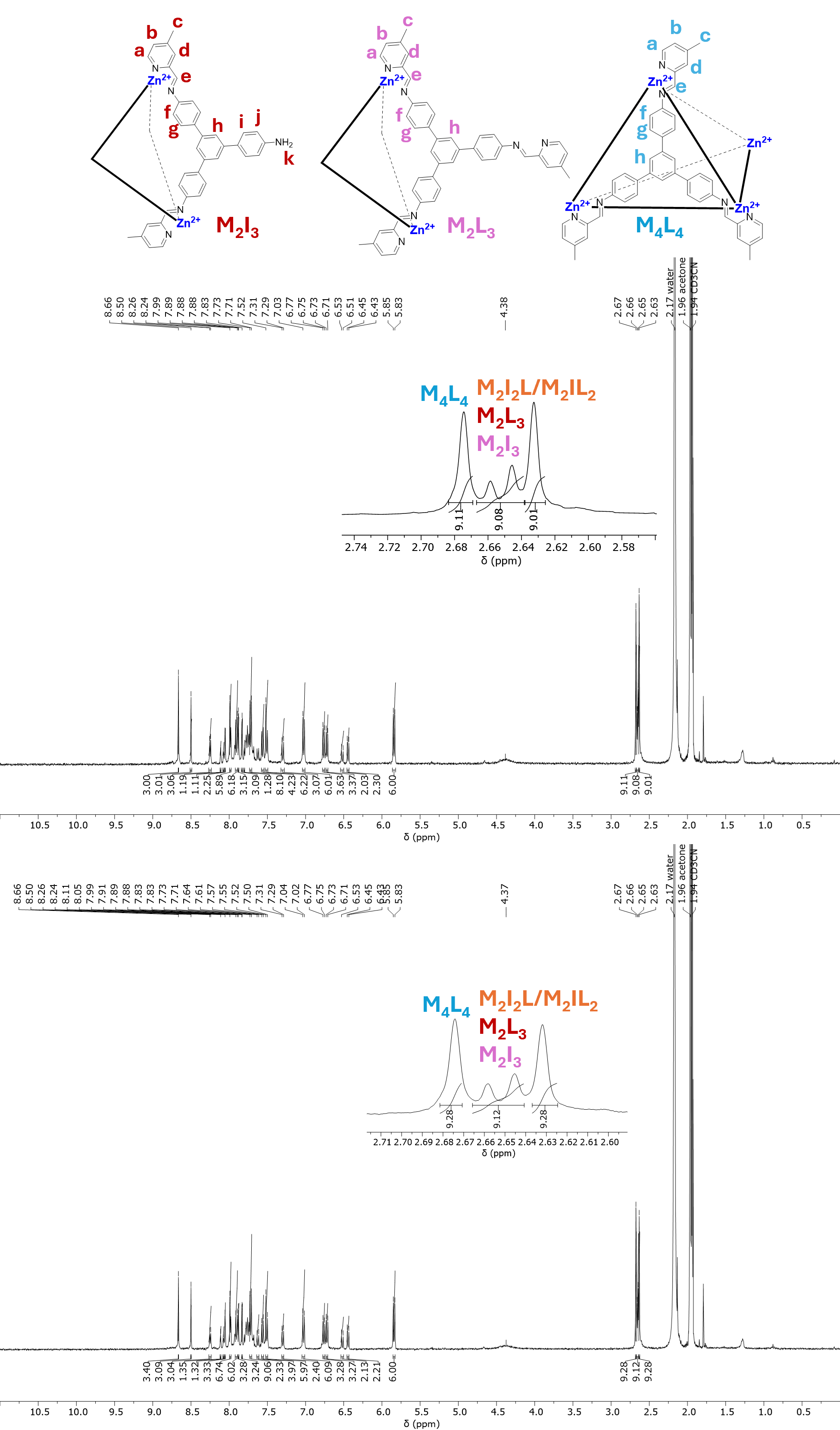


**Figure S10:** ^1^H NMR (CD_3_CN) spectrum of the reaction between triamine **A**, aldehyde **4** and metal counter ion Zn(NTf_2_)_2_ for the first (top) and the second (bottom) repeat screen.


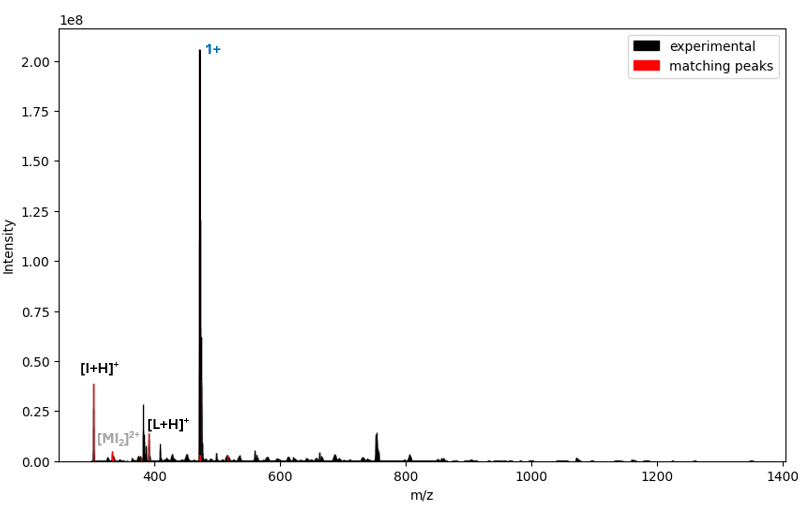

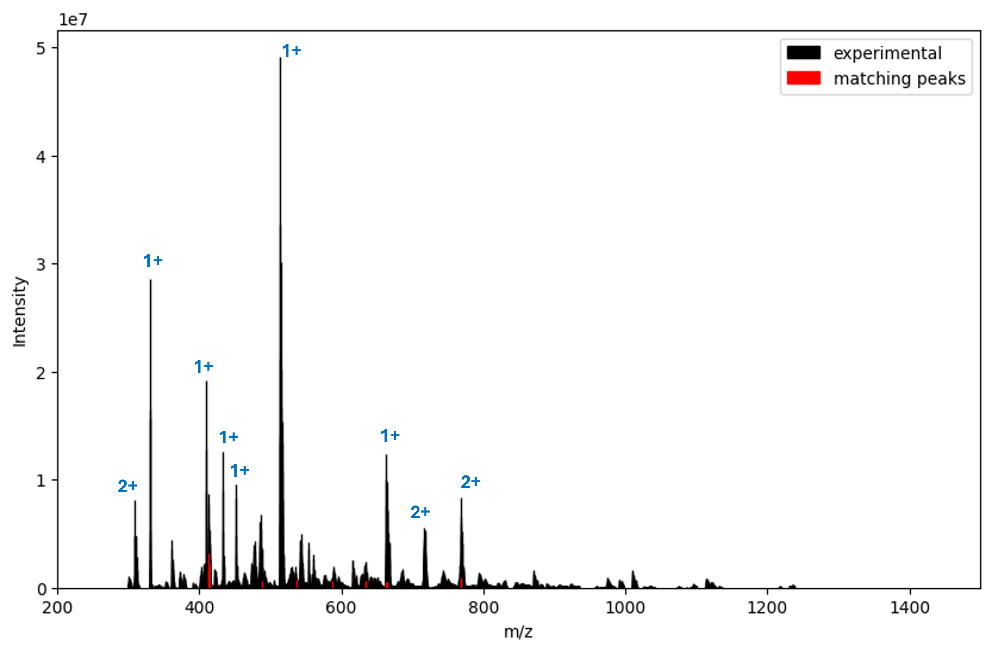


**Figure S11:** HRMS spectrum of the reaction between triamine **B**, aldehyde **1** and metal counter ion Zn(NTf_2_)_2_ for the first (top) and the second (bottom) repeat screen HRMS data in black and the matching peaks from the automated HRMS analysis are identified in red. Peaks of targeted **M_N_L_N_** or **M_2_X_3_** where X = L or I are labelled in black and fragments or intermediates are labelled in grey. Charges of major peaks in HRMS spectrum that were not identified are labelled in blue.

**Figure S12:** ^1^H NMR (CD_3_CN) spectrum of the reaction between triamine **B**, aldehyde **1** and metal counter ion Zn(NTf_2_)_2_ for the first (top) and the second (bottom) repeat screen

**Figure S13:** HRMS spectrum of the reaction between triamine **B**, aldehyde **2** and metal counter ion Zn(NTf_2_)_2_ for the first (top) and the second (bottom) repeat screen HRMS data in black and the matching peaks from the automated HRMS analysis are identified in red. Peaks of targeted **M_N_L_N_** or **M_2_X_3_** where X = L or I are labelled in black and fragments or intermediates are labelled in grey. Charges of major peaks in HRMS spectrum that were not identified are labelled in blue.


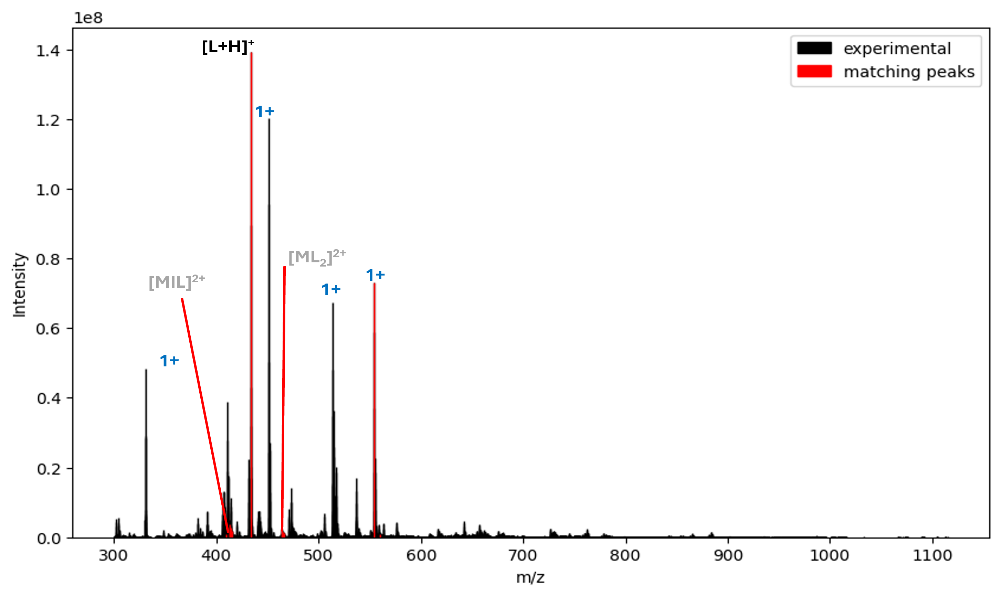

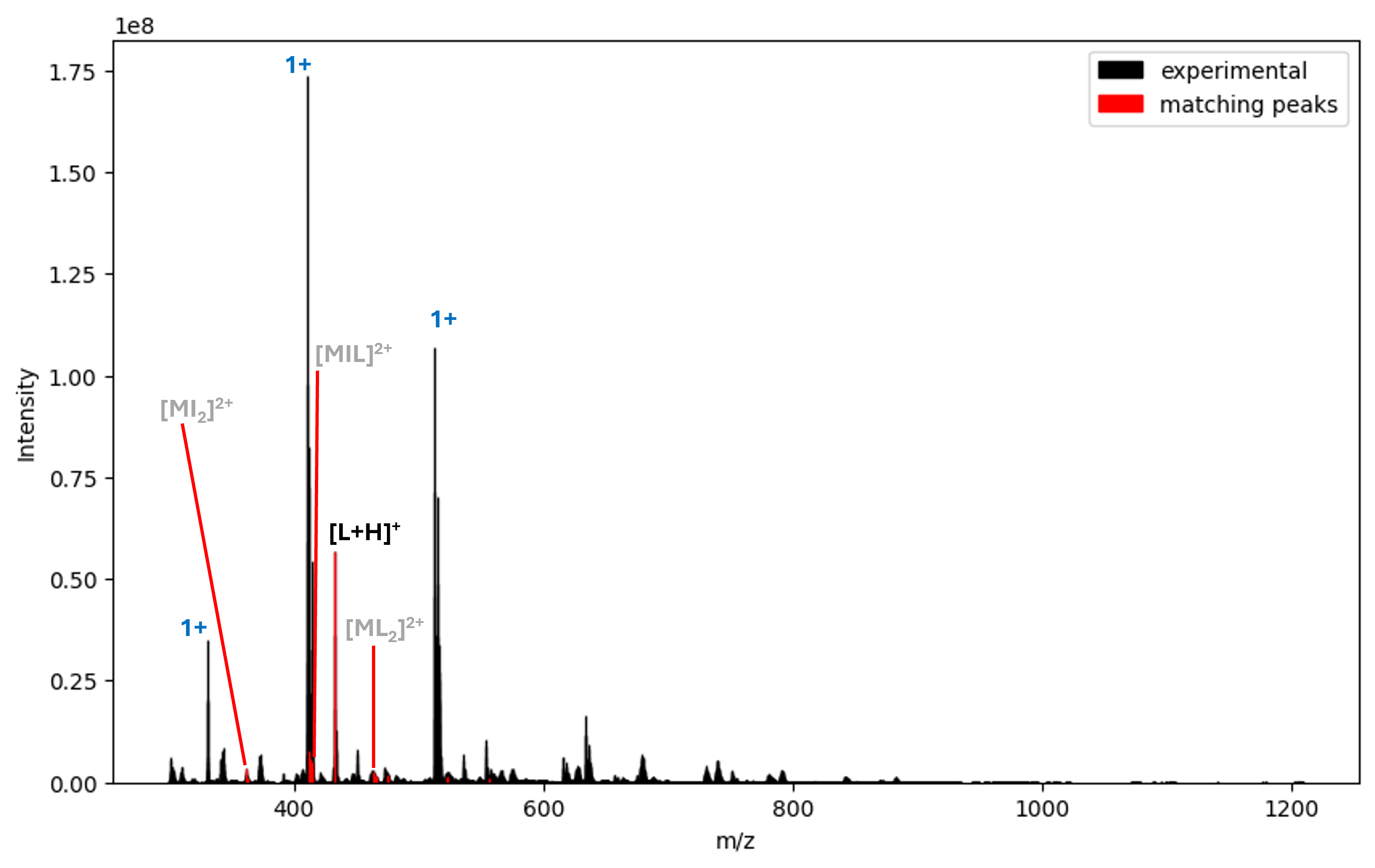


**Figure S14:** ^1^H NMR (CD_3_CN) spectrum of the reaction between triamine **B**, aldehyde **2** and metal counter ion Zn(NTf_2_)_2_ for the first (top) and the second (bottom) repeat screen.

**Figure S15:** HRMS spectrum of the reaction between triamine **B**, aldehyde **3** and metal counter ion Zn(NTf_2_)_2_ for the first (top) and the second (bottom) repeat screen HRMS data in black and the matching peaks from the automated HRMS analysis are identified in red. Peaks of targeted **M_N_L_N_** or **M_2_X_3_** where X = L or I are labelled in black and fragments or intermediates are labelled in grey. Charges of major peaks in HRMS spectrum that were not identified are labelled in blue.


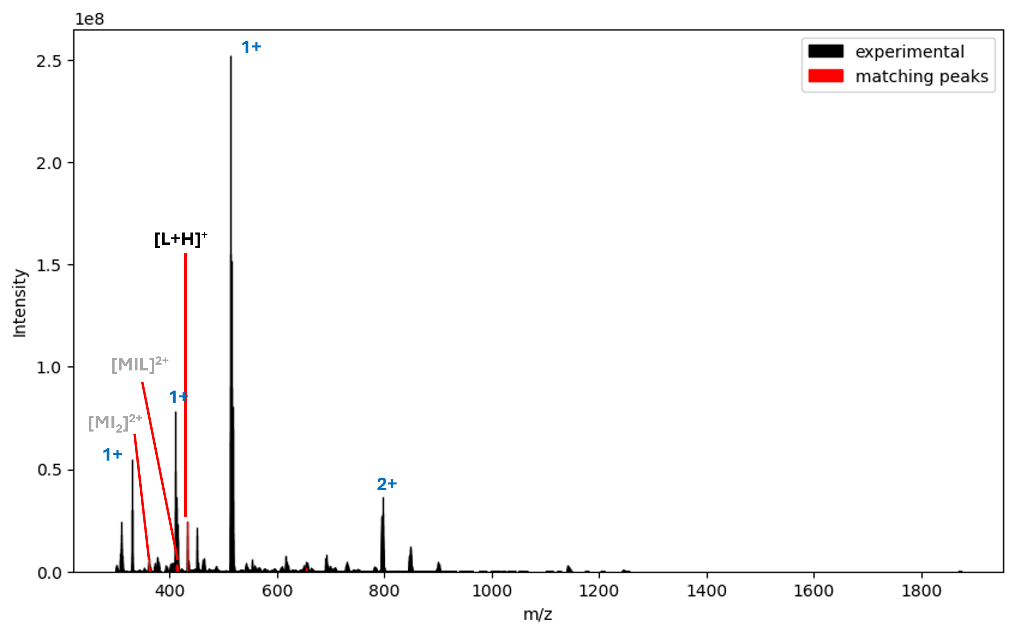

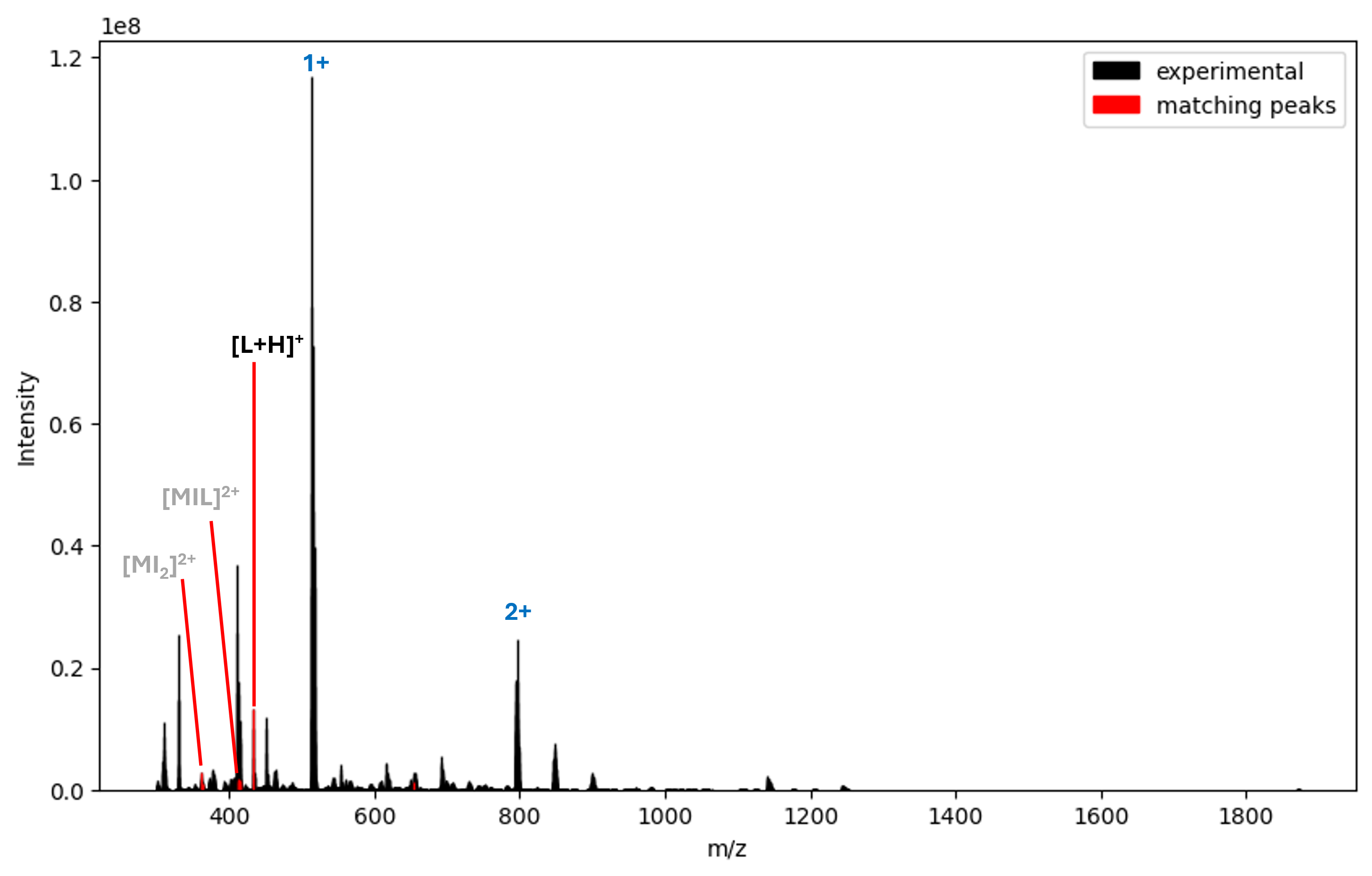


**Figure S16:** ^1^H NMR (CD_3_CN) spectrum of the reaction between triamine **B**, aldehyde **3** and metal counter ion Zn(NTf_2_)_2_ for the first (top) and the second (bottom) repeat screen.


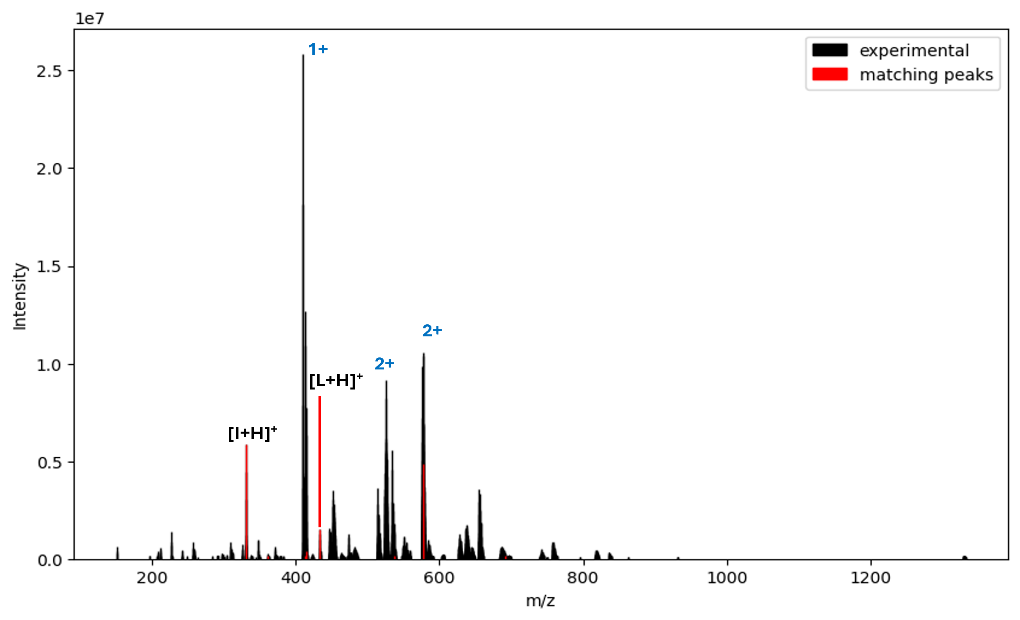

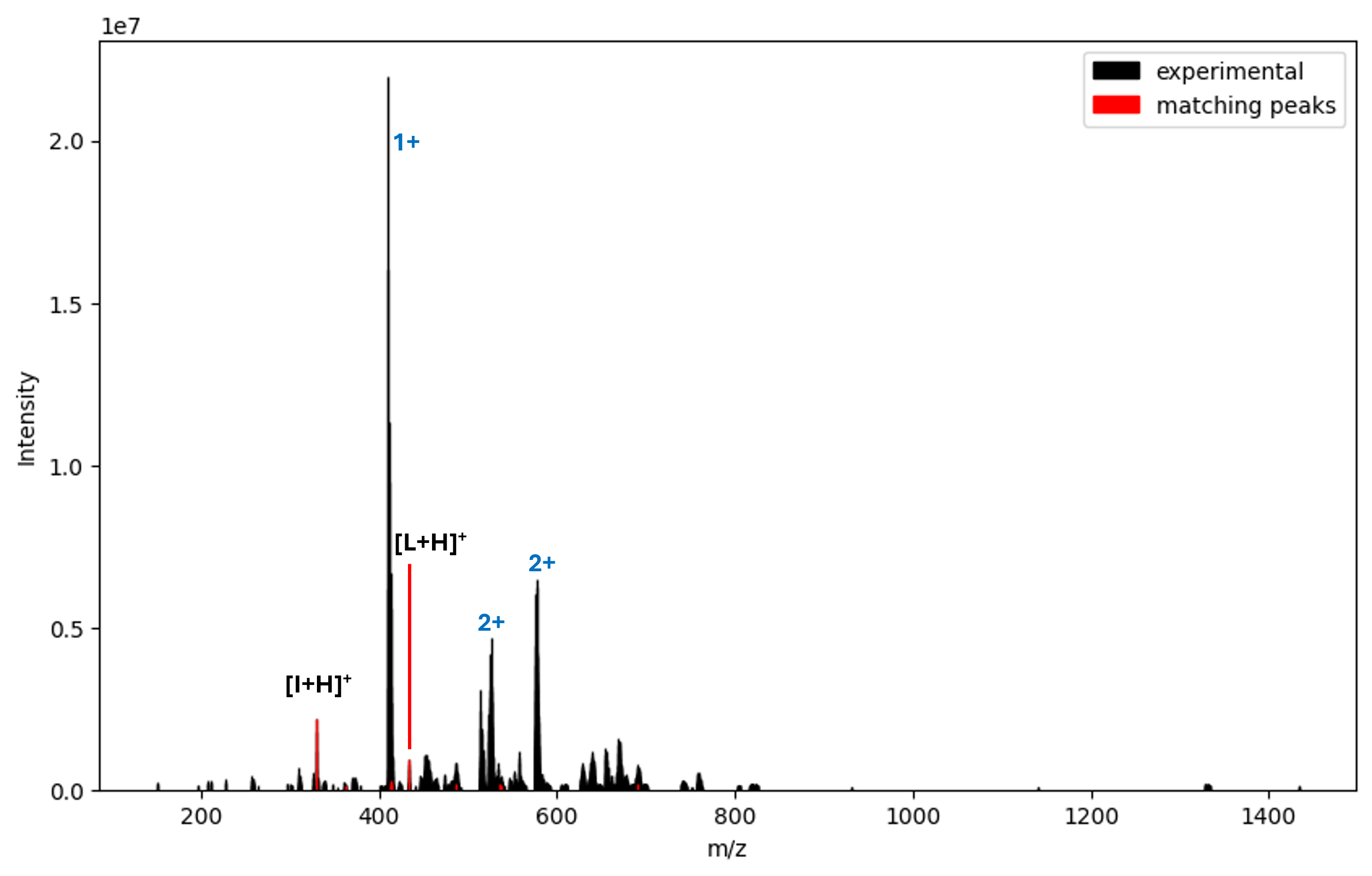


**Figure S17:** HRMS spectrum of the reaction between triamine **B**, aldehyde **4** and metal counter ion Zn(NTf_2_)_2_ for the first (top) and the second (bottom) repeat screen HRMS data in black and the matching peaks from the automated HRMS analysis are identified in red. Peaks of targeted **M_N_L_N_** or **M_2_X_3_** where X = L or I are labelled in black and fragments or intermediates are labelled in grey. Charges of major peaks in HRMS spectrum that were not identified are labelled in blue.

**Figure S18:** ^1^H NMR (CD_3_CN) spectrum of the reaction between triamine **B**, aldehyde **4** and metal counter ion Zn(NTf_2_)_2_ for the first (top) and the second (bottom) repeat screen


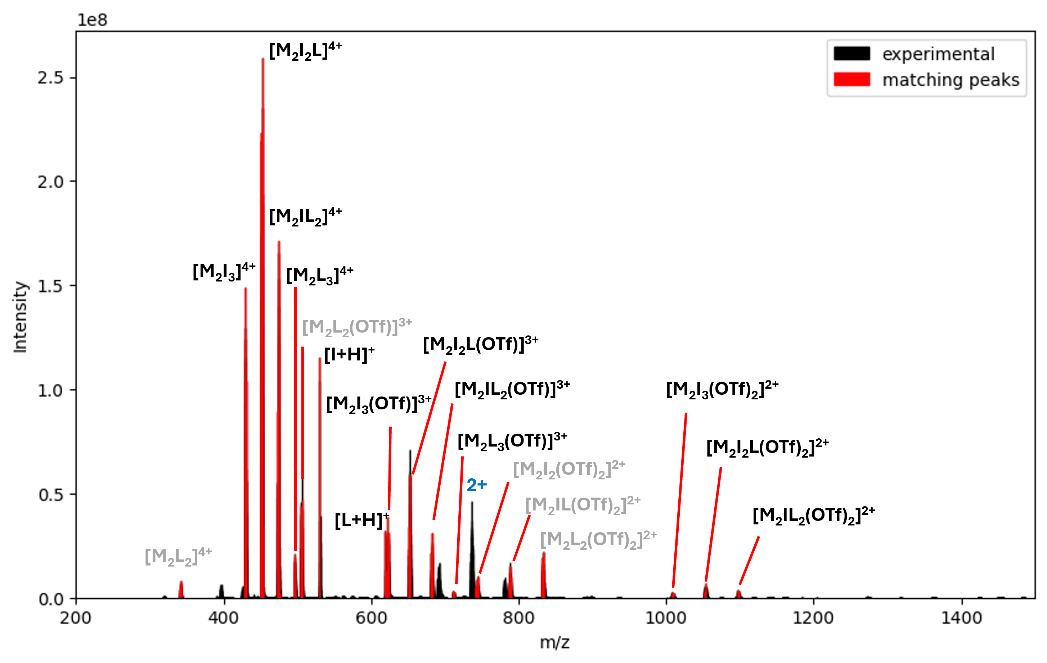

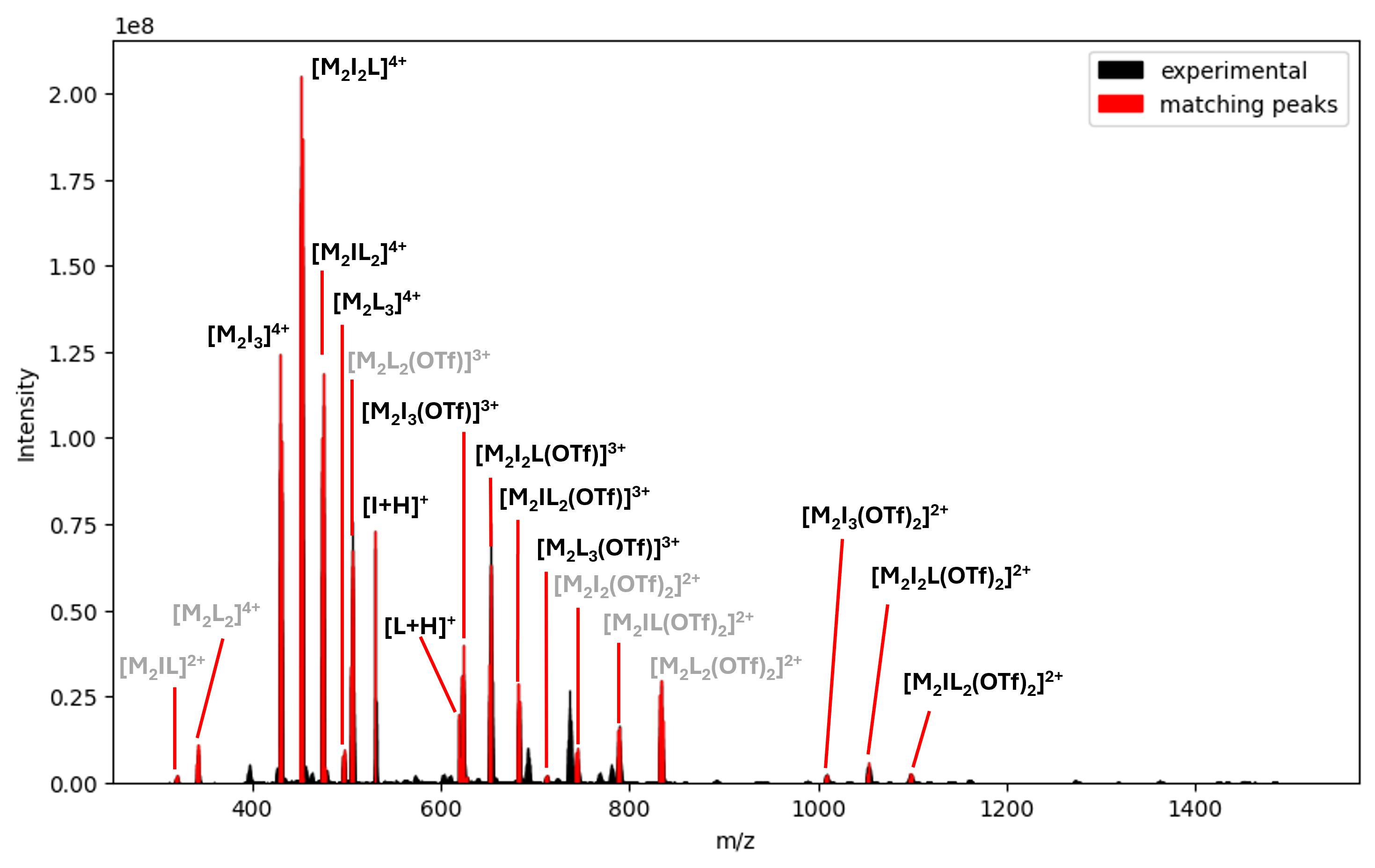


**Figure S19:** HRMS spectrum of the reaction between triamine **A**, aldehyde **1** and metal counter ion Zn(OTf)_2_ for the first (top) and the second (bottom) repeat screen HRMS data in black and the matching peaks from the automated HRMS analysis are identified in red. Peaks of targeted **M_N_L_N_** or **M_2_X_3_** where X = L or I are labelled in black and fragments or intermediates are labelled in grey. Charges of major peaks in HRMS spectrum that were not identified are labelled in blue.

**Figure S20:** ^1^H NMR (CD_3_CN) spectrum of the reaction between triamine **A**, aldehyde **1** and metal counter ion Zn(OTf)_2_ for the first (top) and the second (bottom) repeat screen


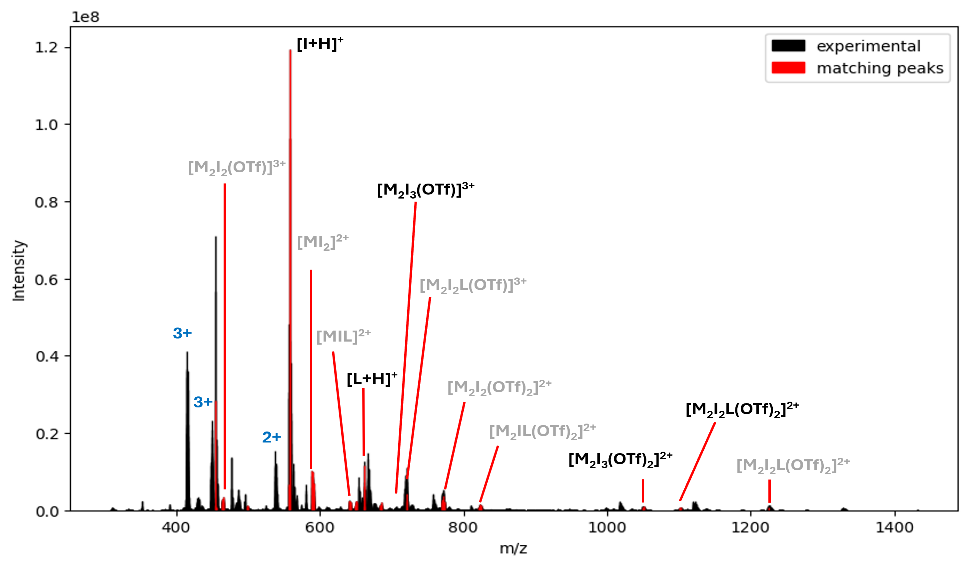

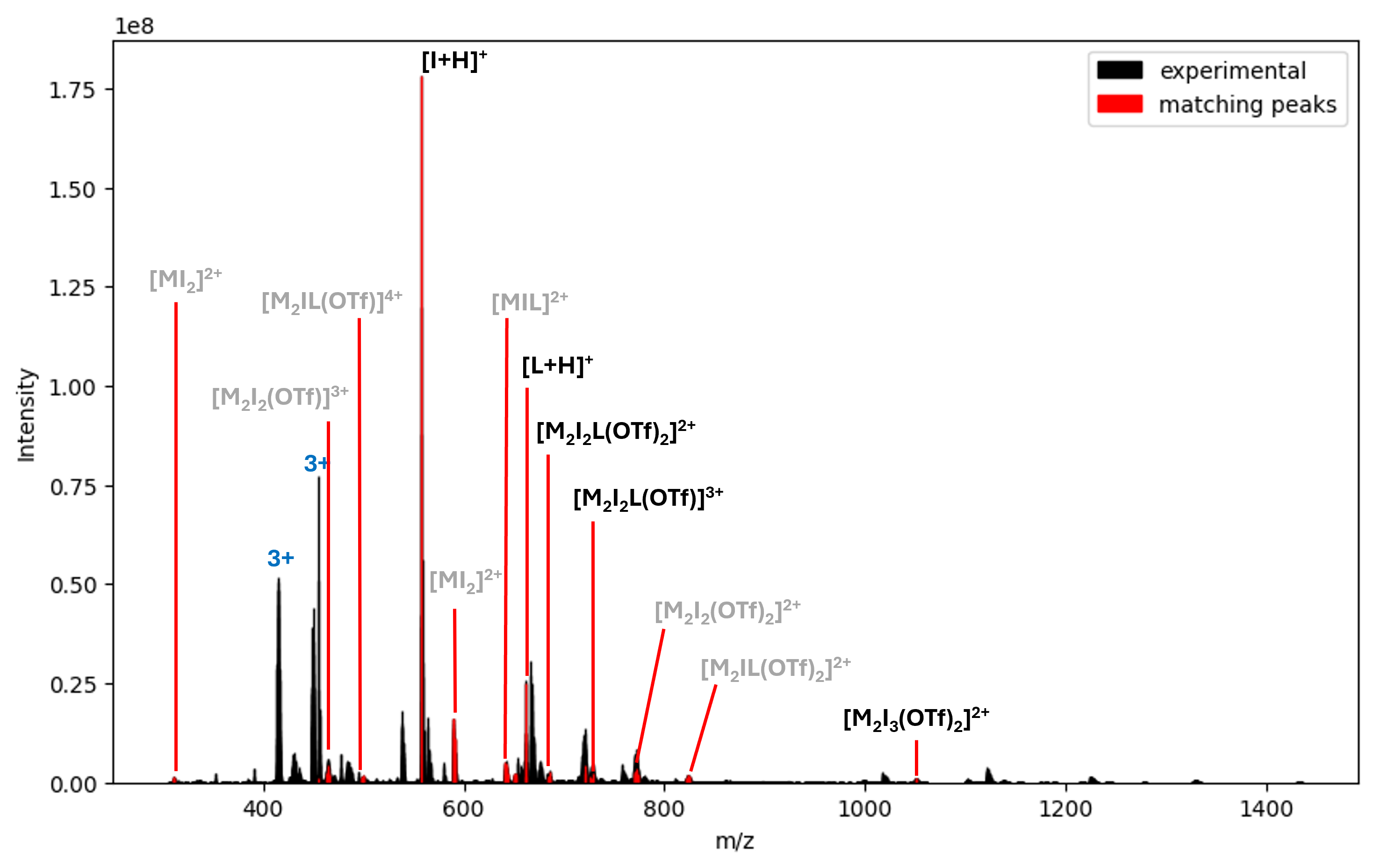


**Figure S21:** HRMS spectrum of the reaction between triamine **A**, aldehyde **2** and metal counter ion Zn(OTf)_2_ for the first (top) and the second (bottom) repeat screen HRMS data in black and the matching peaks from the automated HRMS analysis are identified in red. Peaks of targeted **M_N_L_N_** or **M_2_X_3_** where X = L or I are labelled in black and fragments or intermediates are labelled in grey. Charges of major peaks in HRMS spectrum that were not identified are labelled in blue.

**Figure S22:** ^1^H NMR (CD_3_CN) spectrum of the reaction between triamine **A**, aldehyde **2** and metal counter ion Zn(OTf)_2_ for the first (top) and the second (bottom) repeat screen.


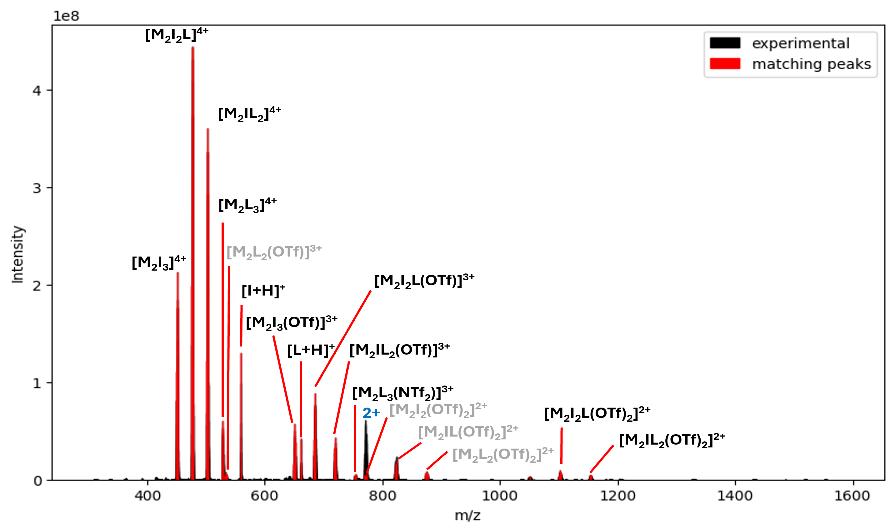

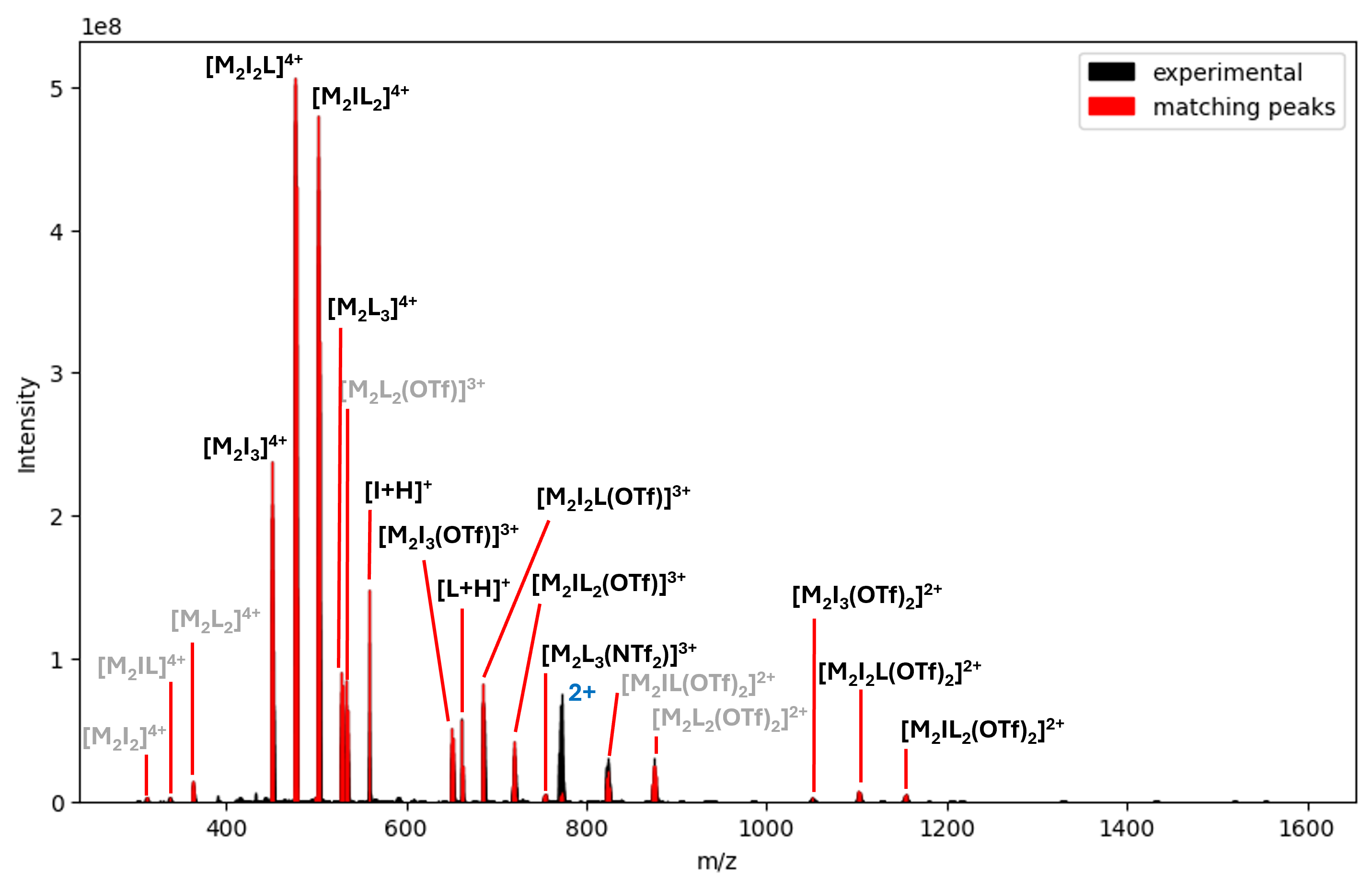


**Figure S23:** HRMS spectrum of the reaction between triamine **A**, aldehyde **3** and metal counter ion Zn(OTf)_2_ for the first (top) and the second (bottom) repeat screen HRMS data in black and the matching peaks from the automated HRMS analysis are identified in red. Peaks of targeted **M_N_L_N_** or **M_2_X_3_** where X = L or I are labelled in black and fragments or intermediates are labelled in grey. Charges of major peaks in HRMS spectrum that were not identified are labelled in blue.

**Figure S24:** ^1^H NMR (CD_3_CN) spectrum of the reaction between triamine **A**, aldehyde **3** and metal counter ion Zn(OTf)_2_ for the first (top) and the second (bottom) repeat screen.


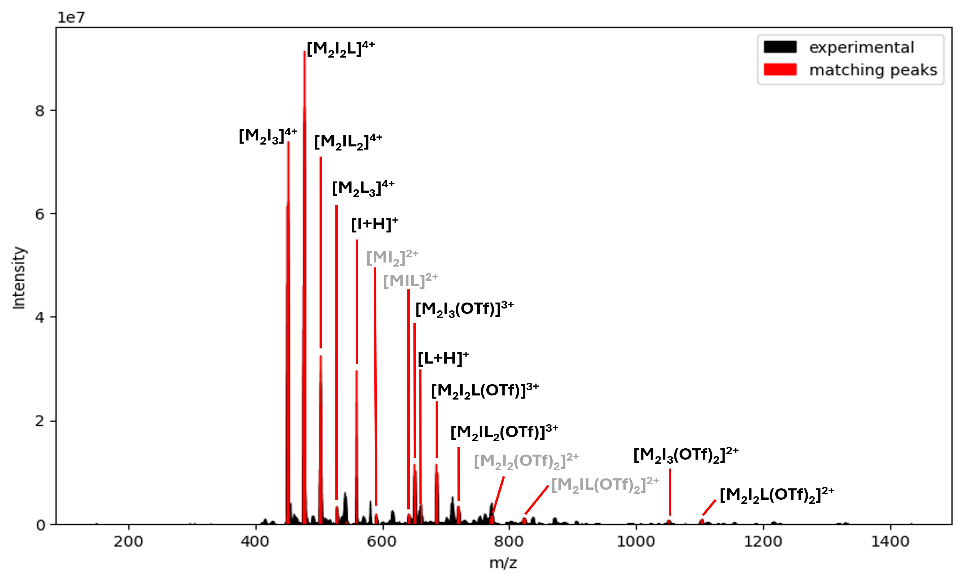

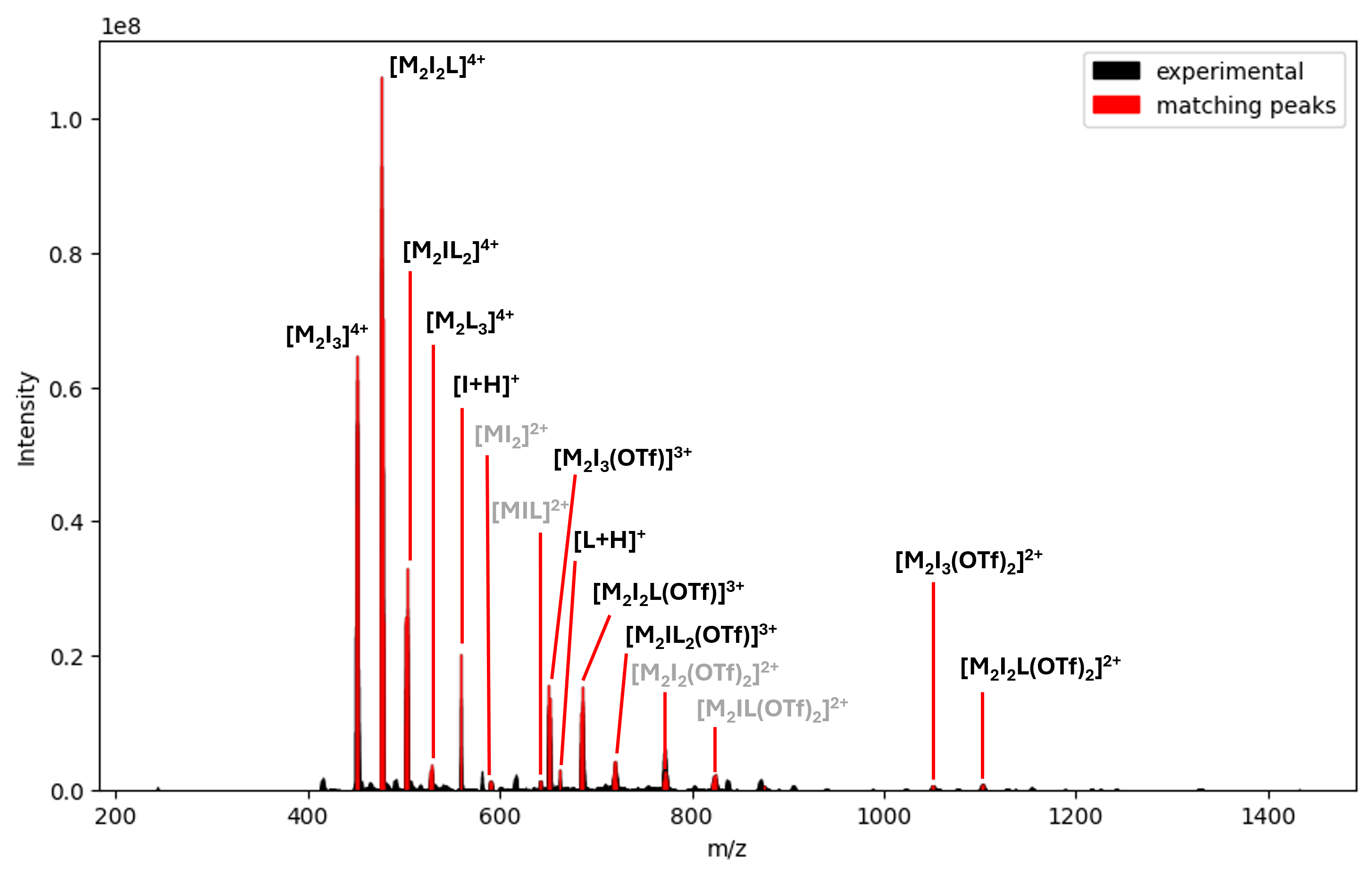


**Figure S25:** HRMS spectrum of the reaction between triamine **A**, aldehyde **4** and metal counter ion Zn(OTf)_2_ for the first (top) and the second (bottom) repeat screen HRMS data in black and the matching peaks from the automated HRMS analysis are identified in red. Peaks of targeted **M_N_L_N_** or **M_2_X_3_** where X = L or I are labelled in black and fragments or intermediates are labelled in grey. Charges of major peaks in HRMS spectrum that were not identified are labelled in blue.


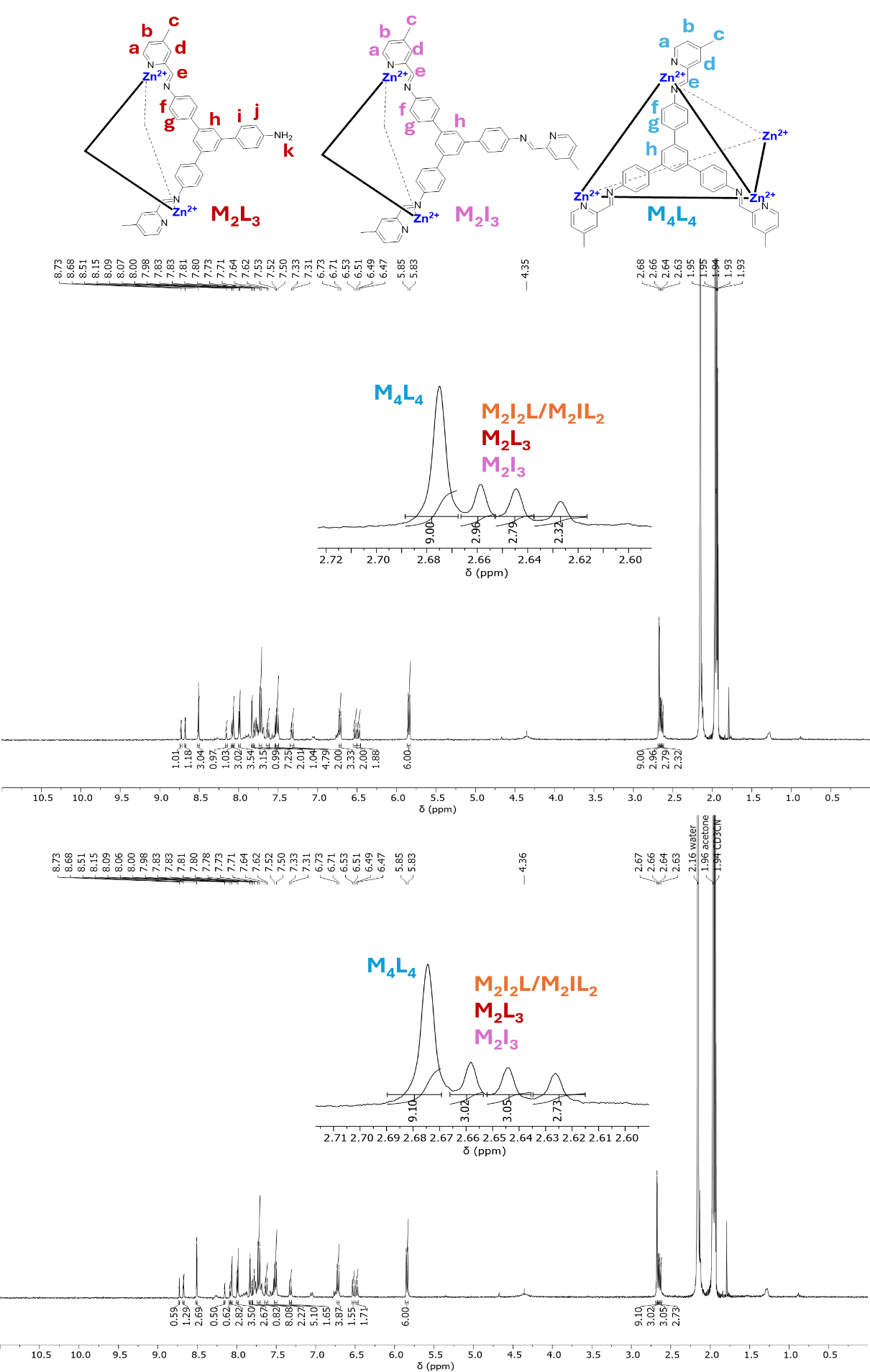


**Figure S26:** ^1^H NMR (CD_3_CN) spectrum of the reaction between triamine **A**, aldehyde **4** and metal counter ion Zn(OTf)_2_ for the first (top) and the second (bottom) repeat screen.


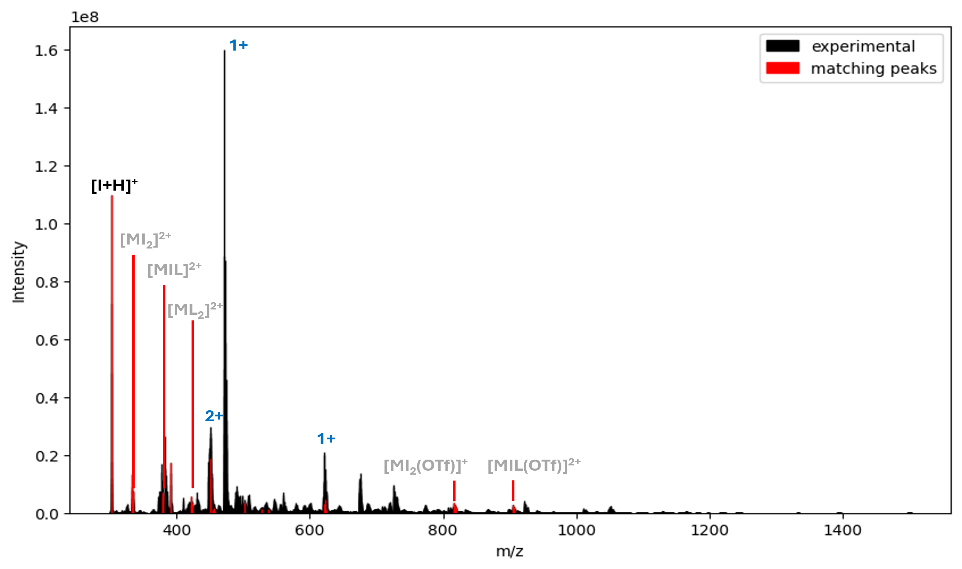

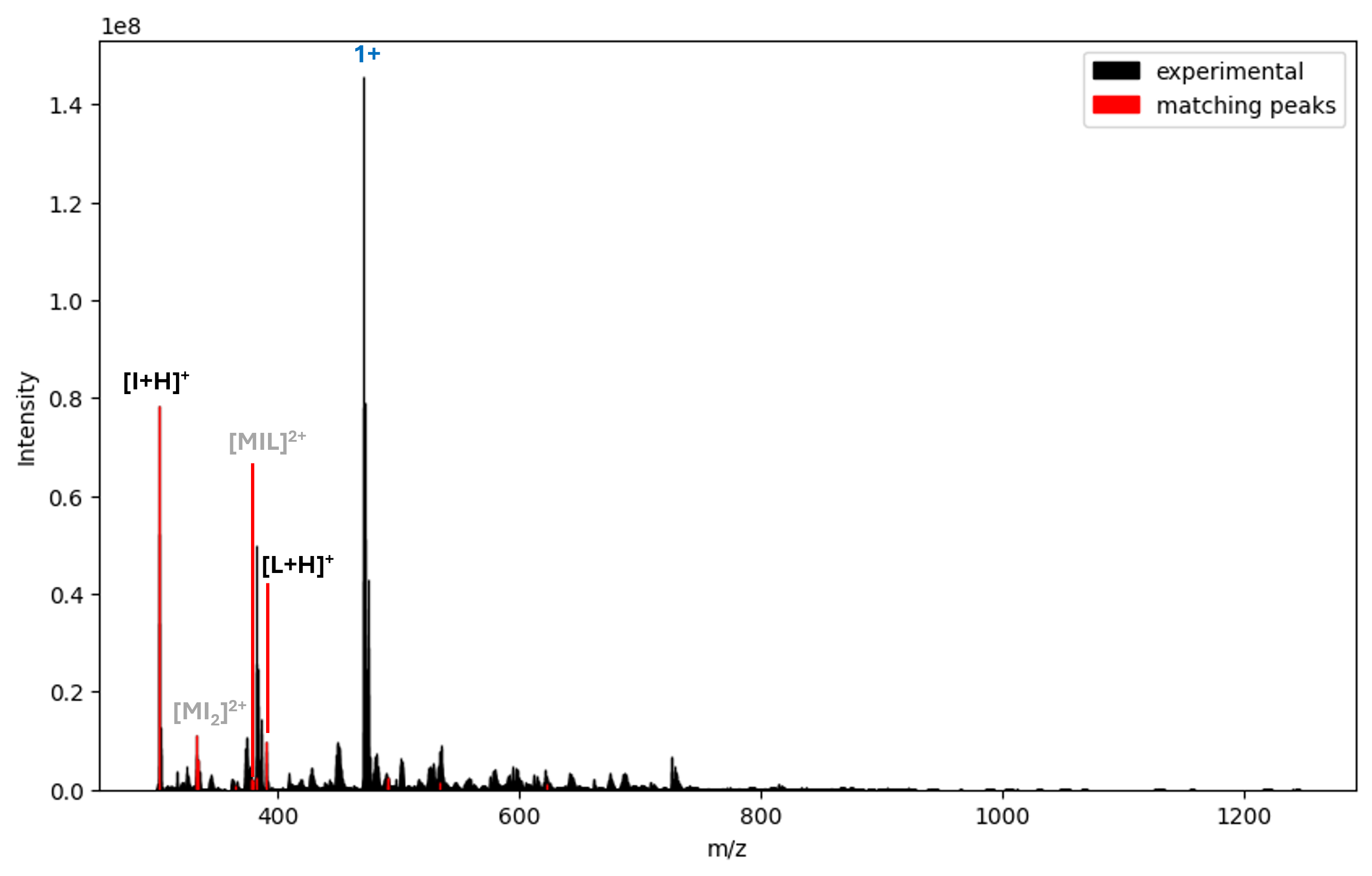


**Figure S27:** HRMS spectrum of the reaction between triamine **B**, aldehyde **1** and metal counter ion Zn(OTf)_2_ for the first (top) and the second (bottom) repeat screen HRMS data in black and the matching peaks from the automated HRMS analysis are identified in red. Peaks of targeted **M_N_L_N_** or **M_2_X_3_** where X = L or I are labelled in black and fragments or intermediates are labelled in grey. Charges of major peaks in HRMS spectrum that were not identified are labelled in blue.

**Figure S28:** ^1^H NMR (CD_3_CN) spectrum of the reaction between triamine **B**, aldehyde **1** and metal counter ion Zn(OTf)_2_ for the first (top) and the second (bottom) repeat screen.


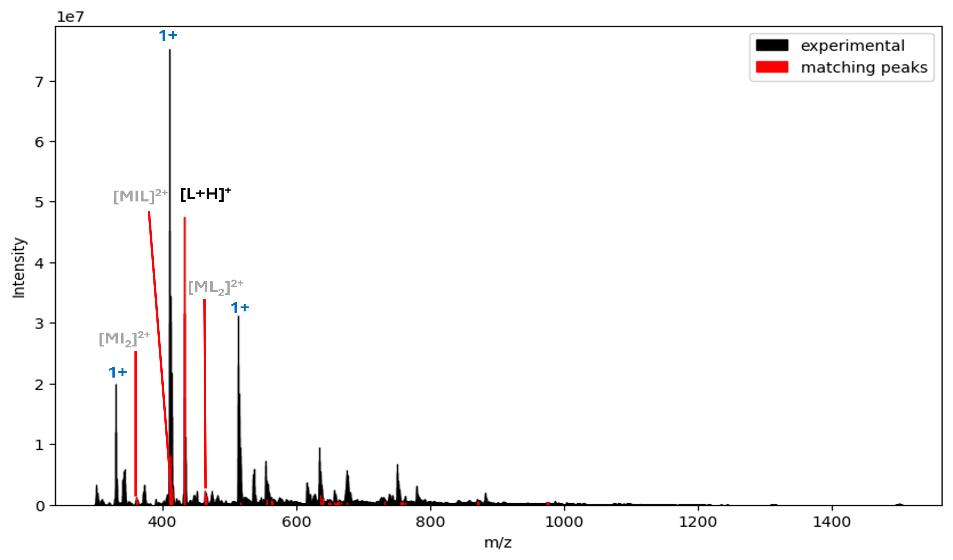

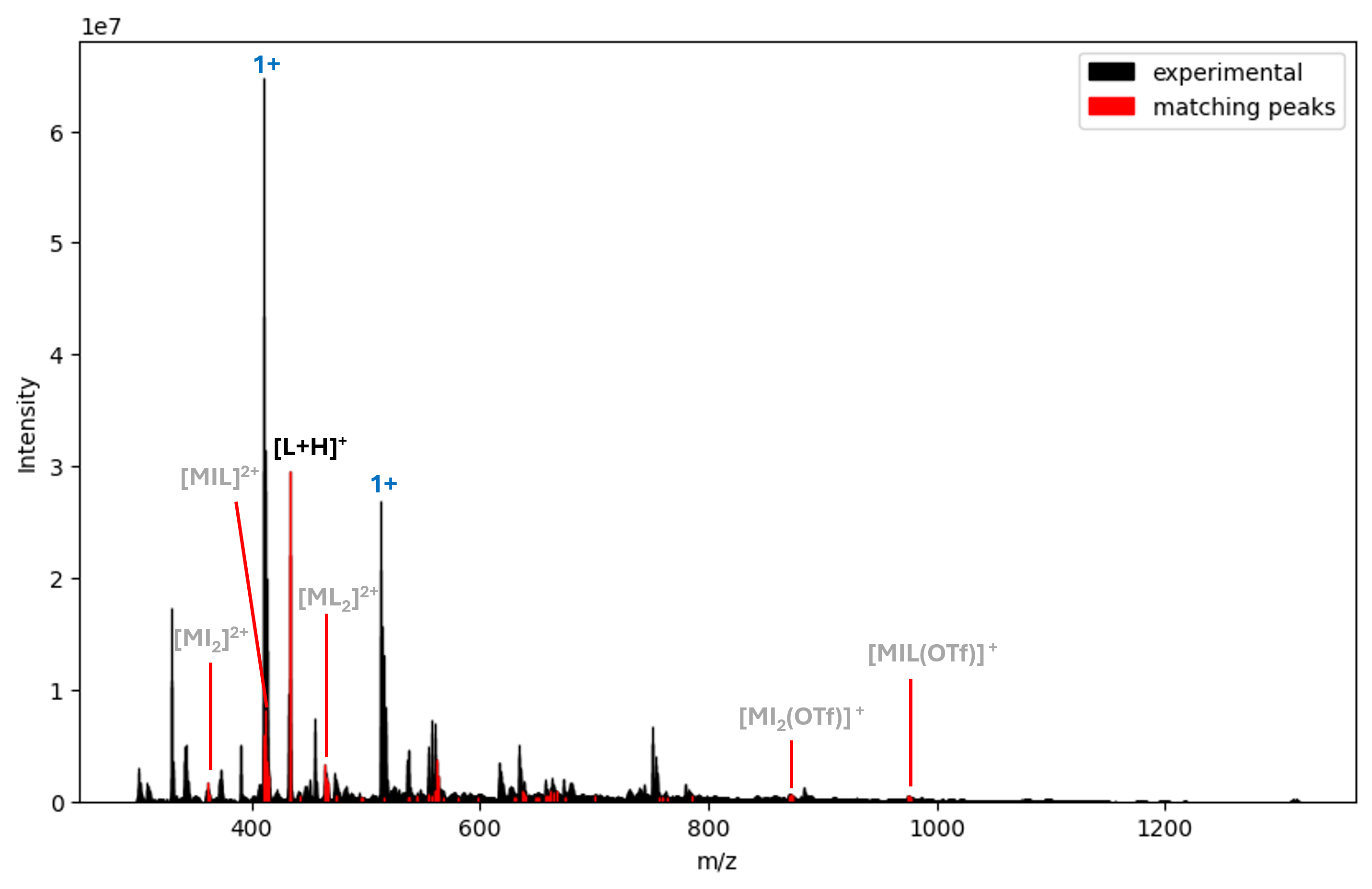


**Figure S29:** HRMS spectrum of the reaction between triamine **B**, aldehyde **2** and metal counter ion Zn(OTf)_2_ for the first (top) and the second (bottom) repeat screen HRMS data in black and the matching peaks from the automated HRMS analysis are identified in red. Peaks of targeted **M_N_L_N_** or **M_2_X_3_** where X = L or I are labelled in black and fragments or intermediates are labelled in grey. Charges of major peaks in HRMS spectrum that were not identified are labelled in blue.

**Figure S30:** ^1^H NMR (CD_3_CN) spectrum of the reaction between triamine **B**, aldehyde **2** and metal counter ion Zn(OTf)_2_ for the first (top) and the second (bottom) repeat screen.


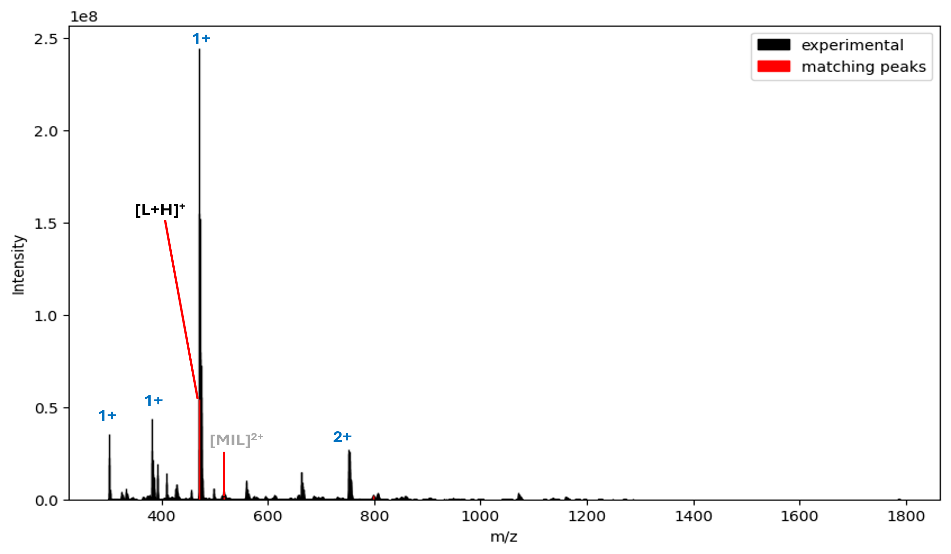

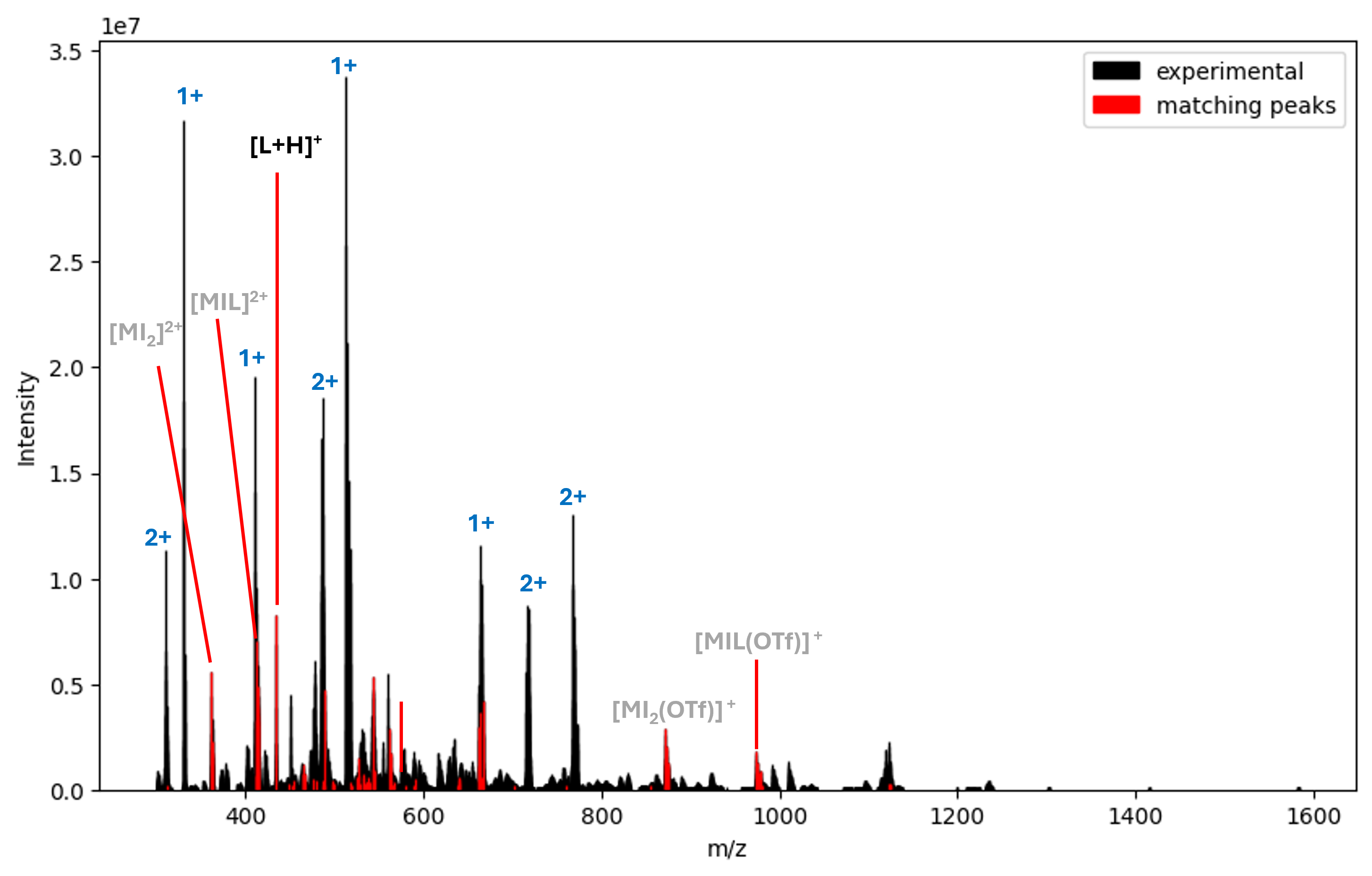


**Figure S31:** HRMS spectrum of the reaction between triamine **B**, aldehyde **3** and metal counter ion Zn(OTf)_2_ for the first (top) and the second (bottom) repeat screen HRMS data in black and the matching peaks from the automated HRMS analysis are identified in red. Peaks of targeted **M_N_L_N_** or **M_2_X_3_** where X = L or I are labelled in black and fragments or intermediates are labelled in grey. Charges of major peaks in HRMS spectrum that were not identified are labelled in blue.

**Figure S32:** ^1^H NMR (CD_3_CN) spectrum of the reaction between triamine **B**, aldehyde **3** and metal counter ion Zn(OTf)_2_ for the first (top) and the second (bottom) repeat screen.


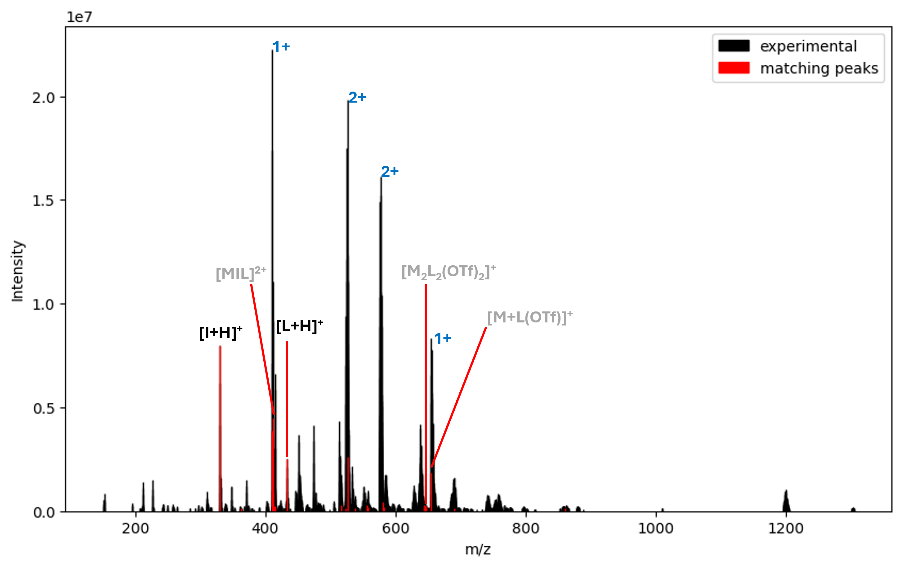

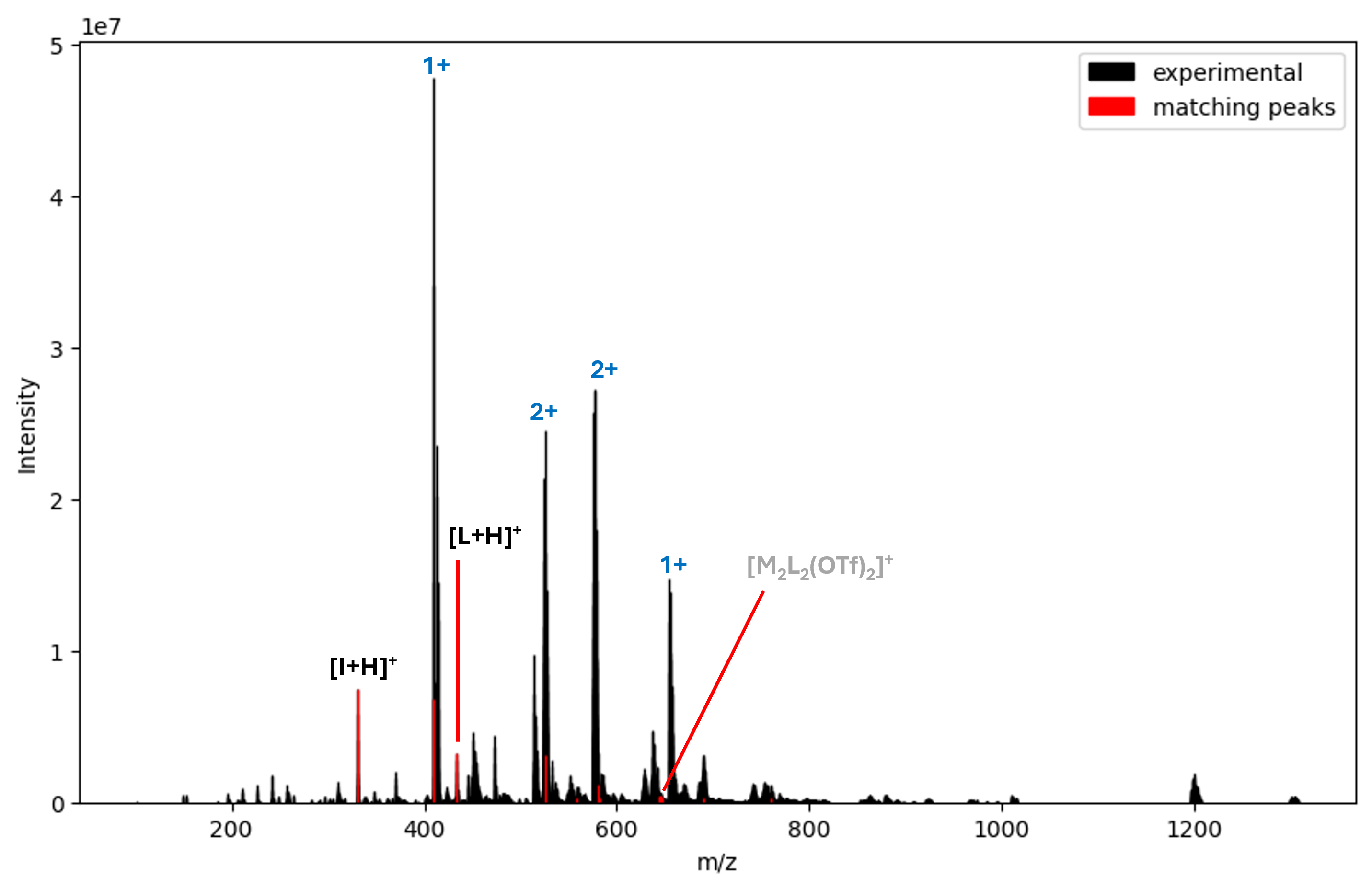


**Figure S33:** HRMS spectrum of the reaction between triamine **B**, aldehyde **4** and metal counter ion Zn(OTf)_2_ for the first (top) and the second (bottom) repeat screen HRMS data in black and the matching peaks from the automated HRMS analysis are identified in red. Peaks of targeted **M_N_L_N_** or **M_2_X_3_** where X = L or I are labelled in black and fragments or intermediates are labelled in grey. Charges of major peaks in HRMS spectrum that were not identified are labelled in blue.

**Figure S34:** ^1^H NMR (CD_3_CN) spectrum of the reaction between triamine **B**, aldehyde **4** and metal counter ion Zn(OTf)_2_ for the first (top) and the second (bottom) repeat screen.


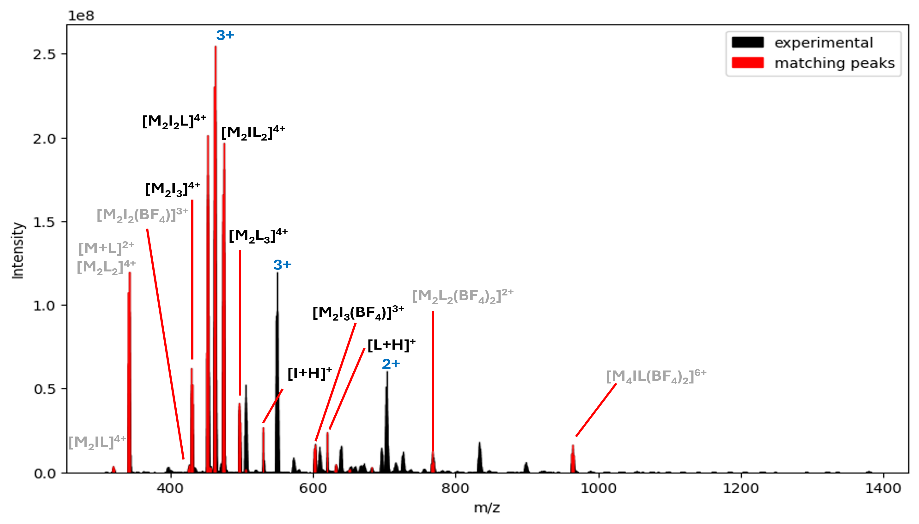

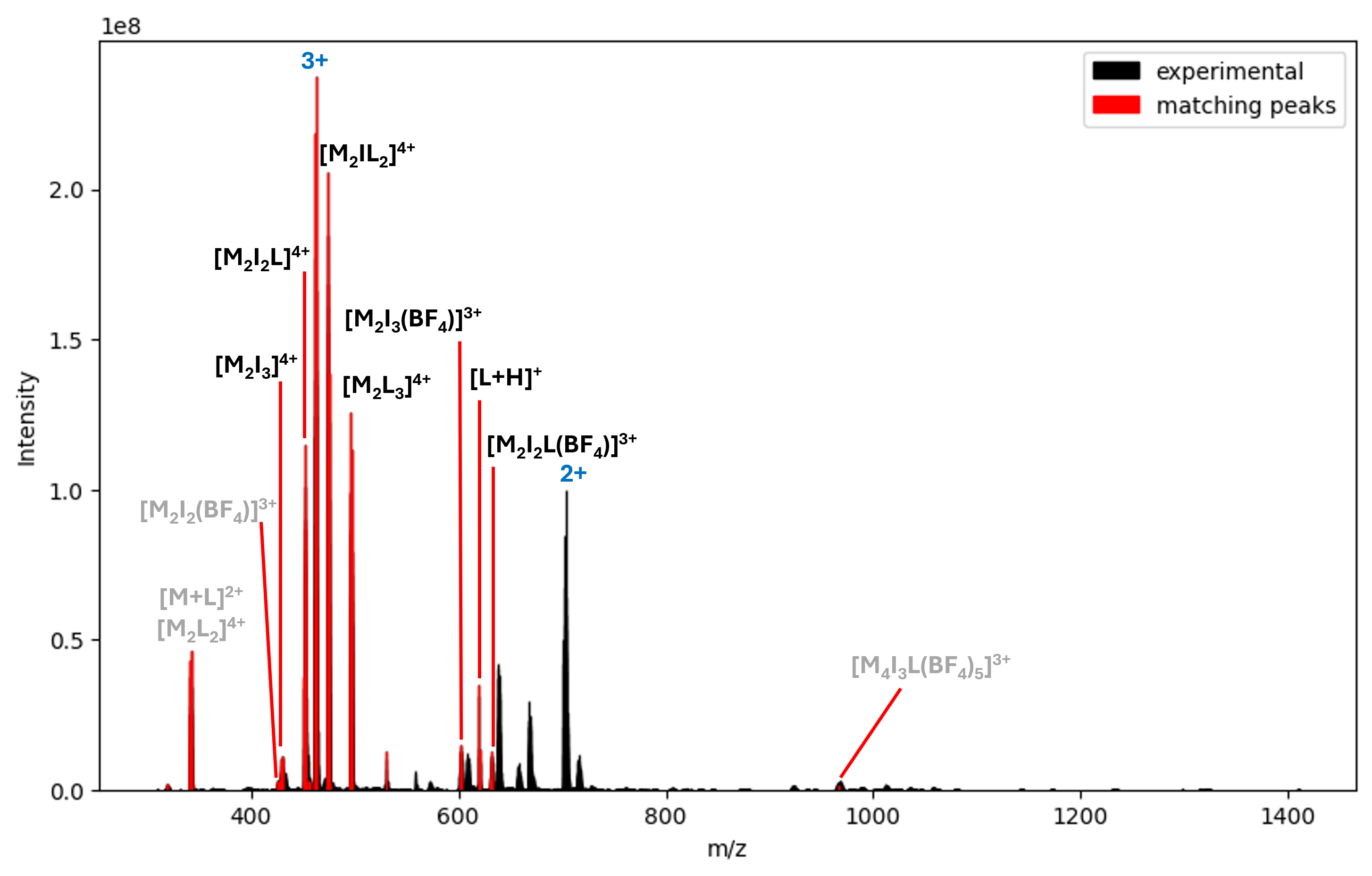


**Figure S35:** HRMS spectrum of the reaction between triamine **A**, aldehyde **1** and metal counter ion Zn(BF_4_)_2_ for the first (top) and the second (bottom) repeat screen HRMS data in black and the matching peaks from the automated HRMS analysis are identified in red. Peaks of targeted **M_N_L_N_** or **M_2_X_3_** where X = L or I are labelled in black and fragments or intermediates are labelled in grey. Charges of major peaks in HRMS spectrum that were not identified are labelled in blue.

**a**

**a**

**b**

**b,d**

**c**

**c**

**d**

**e**

**e**

**f**

**g**

**h**

**h**

**f**

**g**

**a**

**b,d**

**c**

**e**

**h**

**f**

**g**

**Figure S36:** ^1^H NMR (CD_3_CN) spectrum of the reaction between triamine **A**, aldehyde **1** and metal counter ion Zn(BF_4_)_2_ for the first (top) and the second (bottom) repeat screen.


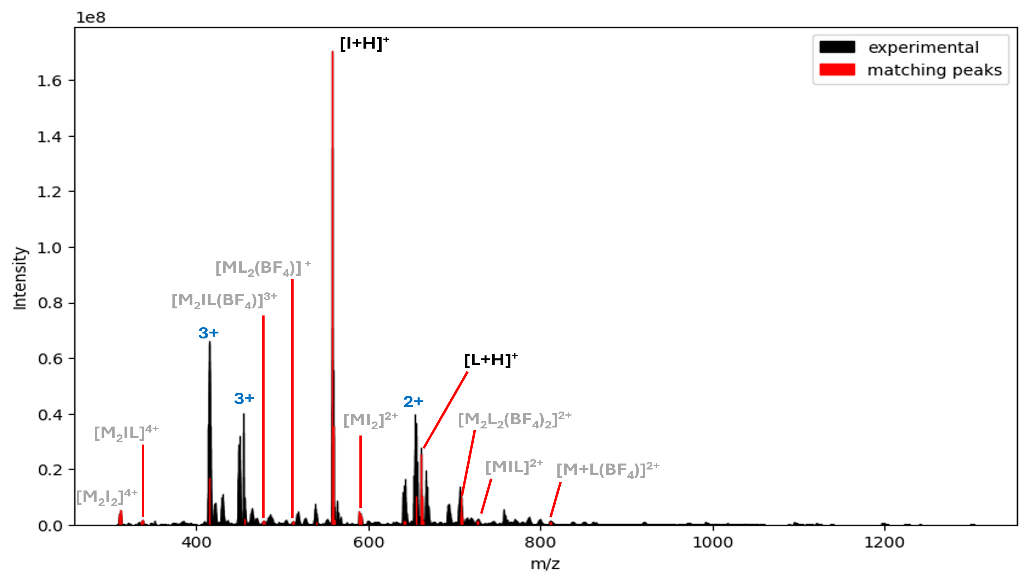

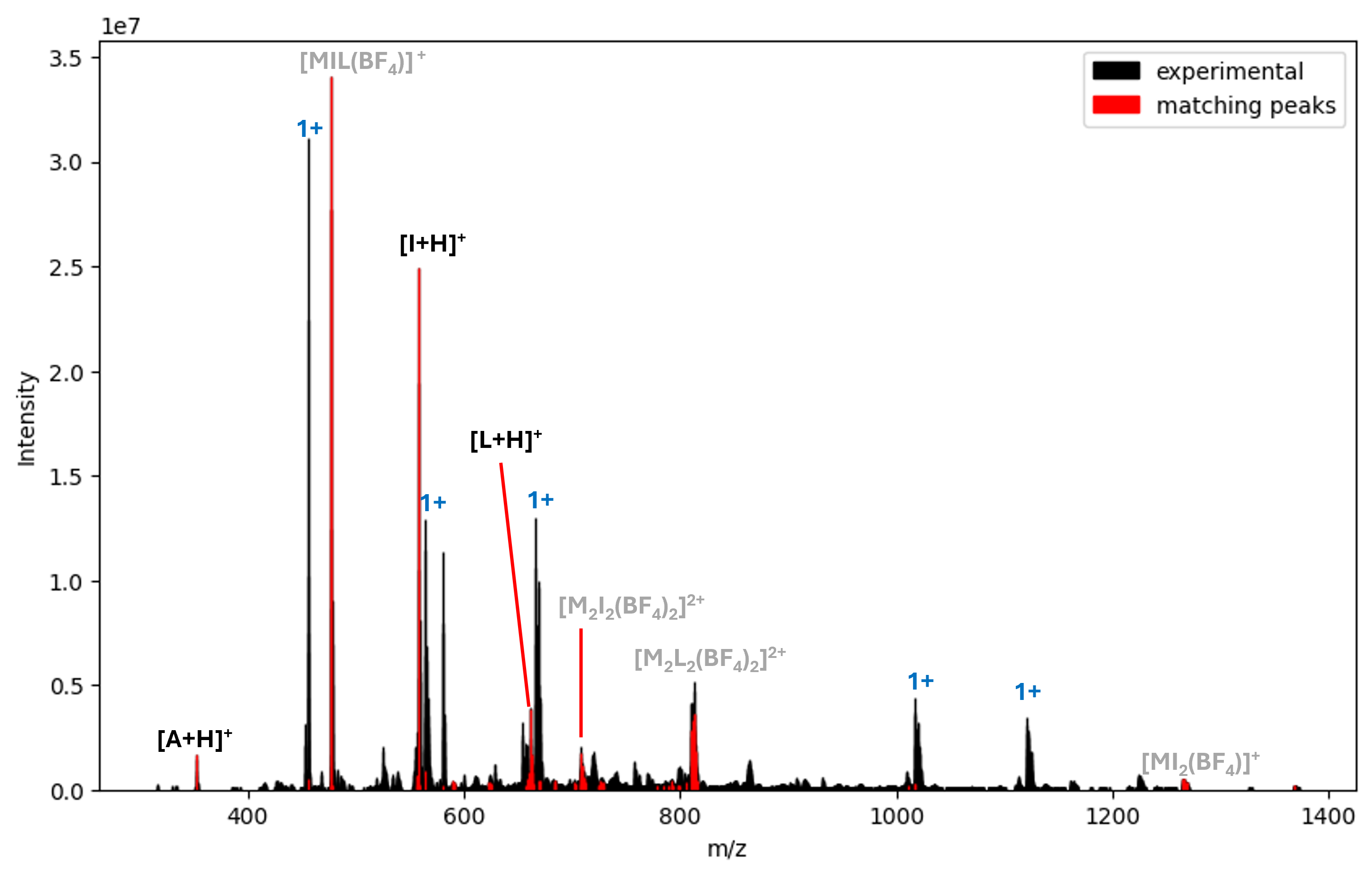


**Figure S37:** HRMS spectrum of the reaction between triamine **A**, aldehyde **2** and metal counter ion Zn(BF_4_)_2_ for the first (top) and the second (bottom) repeat screen HRMS data in black and the matching peaks from the automated HRMS analysis are identified in red. Peaks of targeted **M_N_L_N_** or **M_2_X_3_** where X = L or I are labelled in black and fragments or intermediates are labelled in grey. Charges of major peaks in HRMS spectrum that were not identified are labelled in blue.

**Figure S38:** ^1^H NMR (CD_3_CN) spectrum of the reaction between triamine **A**, aldehyde **2** and metal counter ion Zn(BF_4_)_2_ for the first (top) and the second (bottom) repeat screen.


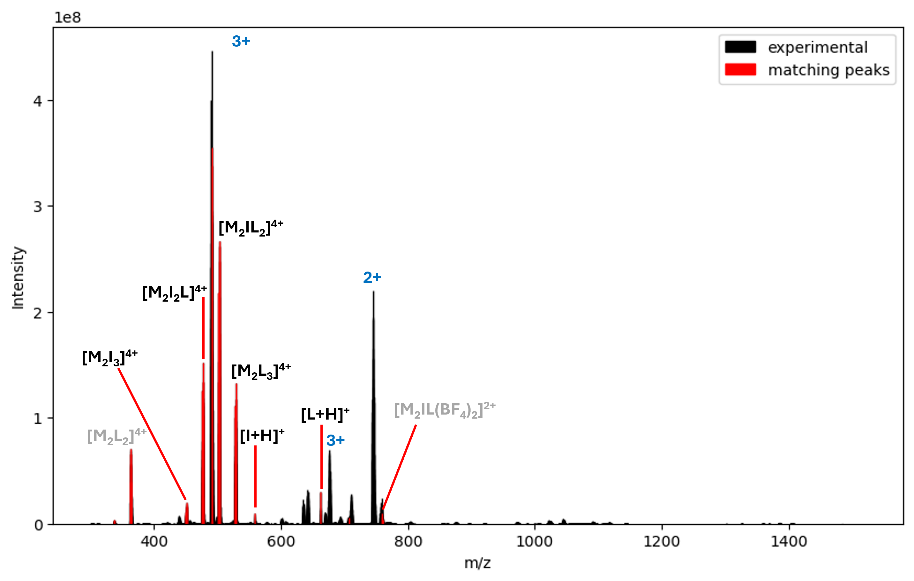

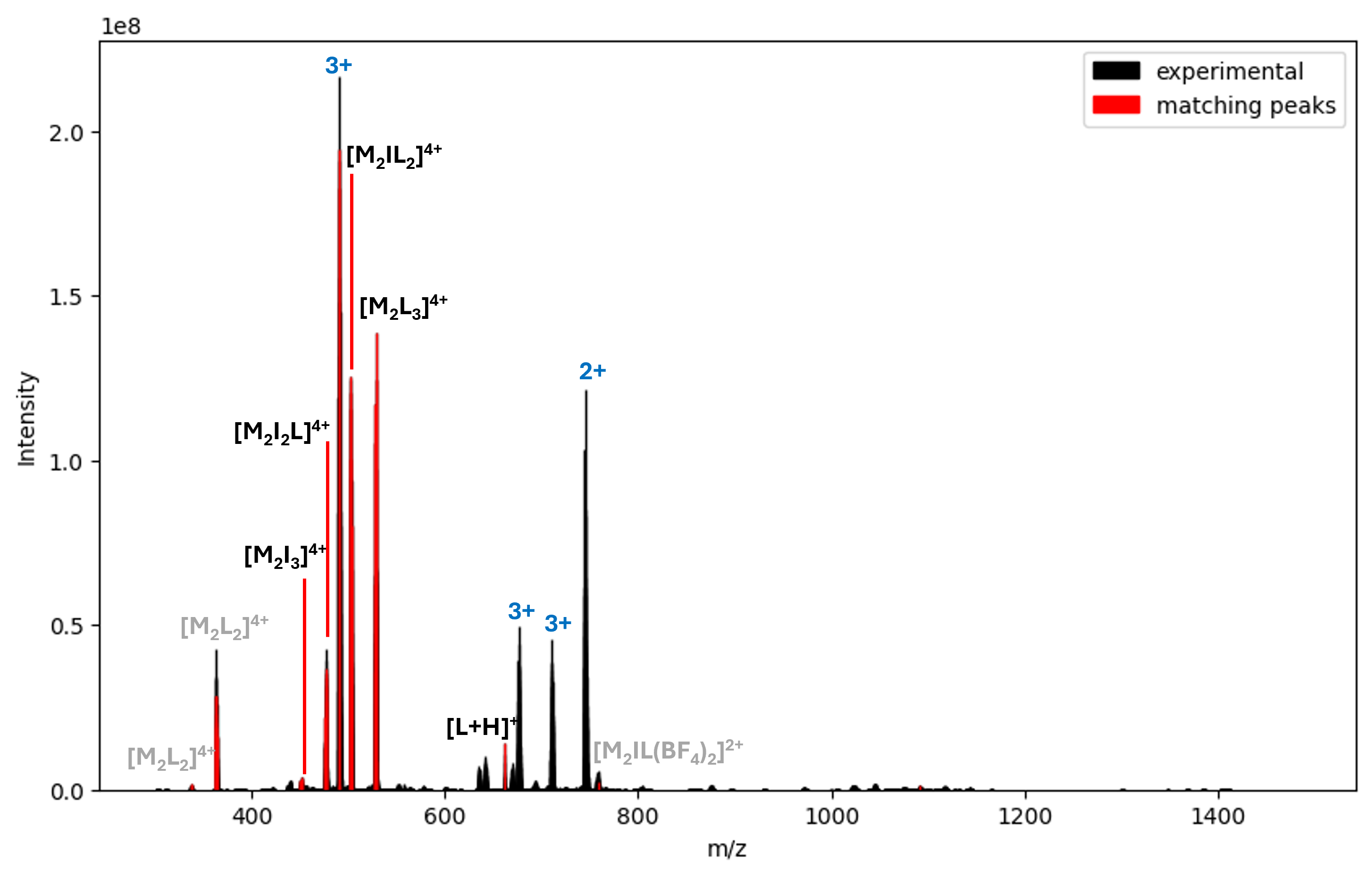


**Figure S39:** HRMS spectrum of the reaction between triamine **A**, aldehyde **3** and metal counter ion Zn(BF_4_)_2_ for the first (top) and the second (bottom) repeat screen HRMS data in black and the matching peaks from the automated HRMS analysis are identified in red. Peaks of targeted **M_N_L_N_** or **M_2_X_3_** where X = L or I are labelled in black and fragments or intermediates are labelled in grey. Charges of major peaks in HRMS spectrum that were not identified are labelled in blue.

**a**

**a**

**b**

**d**

**c**

**c**

**d**

**e**

**e**

**f**

**g**

**h**

**h**

**f**

**g**

**b**

**a**

**d**

**c**

**e**

**h**

**f**

**g**

**b**

**Figure S40:** ^1^H NMR (CD_3_CN) spectrum of the reaction between triamine **A**, aldehyde **3** and metal counter ion Zn(BF_4_)_2_ for the first (top) and the second (bottom) repeat screen.


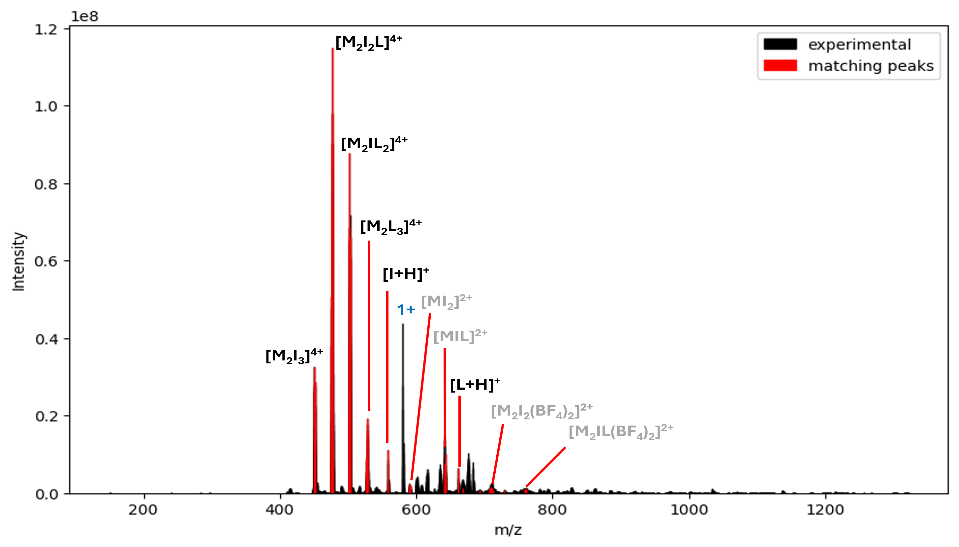

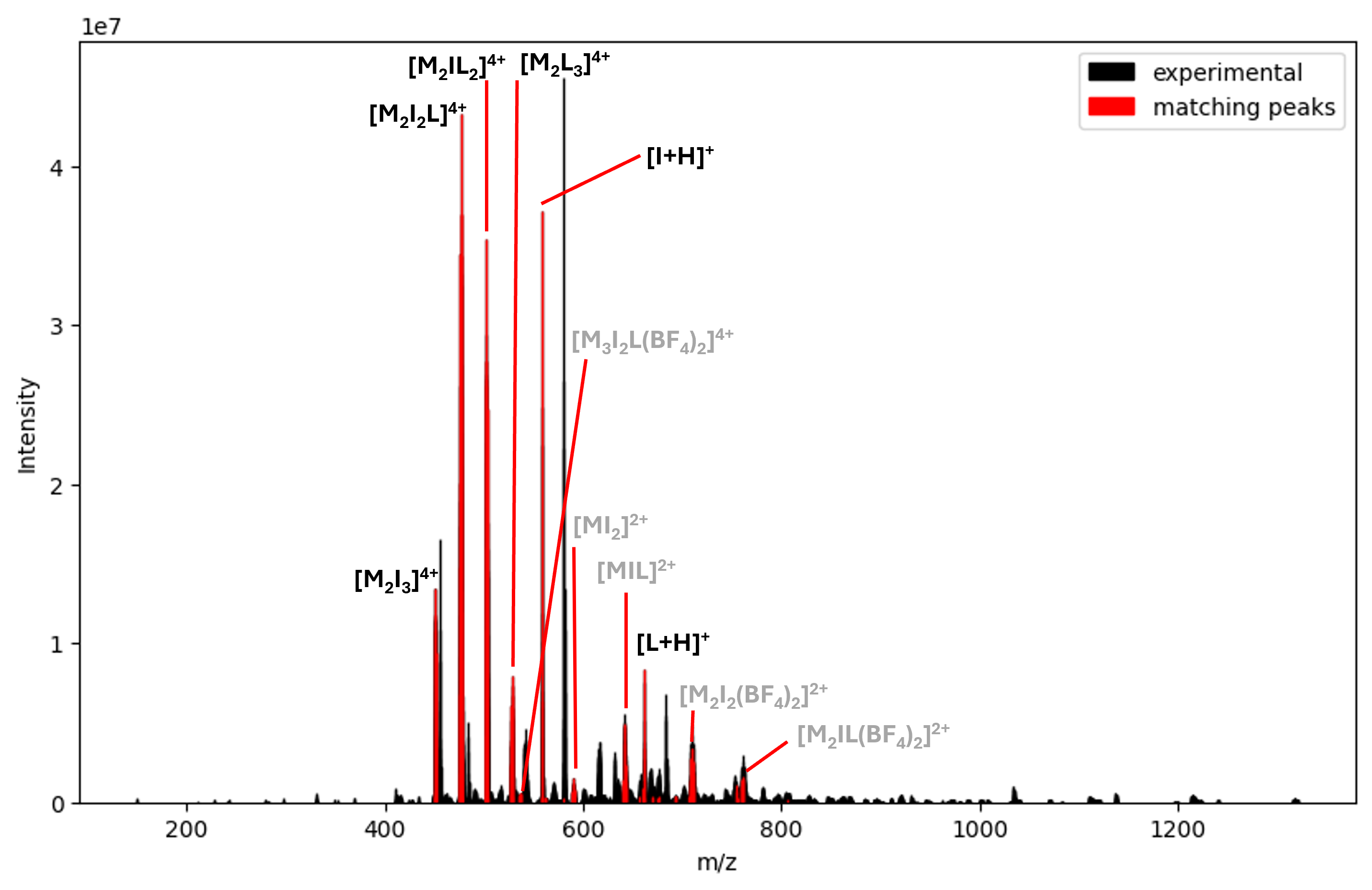


**Figure S41:** HRMS spectrum of the reaction between triamine **A**, aldehyde **4** and metal counter ion Zn(BF_4_)_2_ for the first (top) and the second (bottom) repeat screen HRMS data in black and the matching peaks from the automated HRMS analysis are identified in red. Peaks of targeted **M_N_L_N_** or **M_2_X_3_** where X = L or I are labelled in black and fragments or intermediates are labelled in grey. Charges of major peaks in HRMS spectrum that were not identified are labelled in blue.


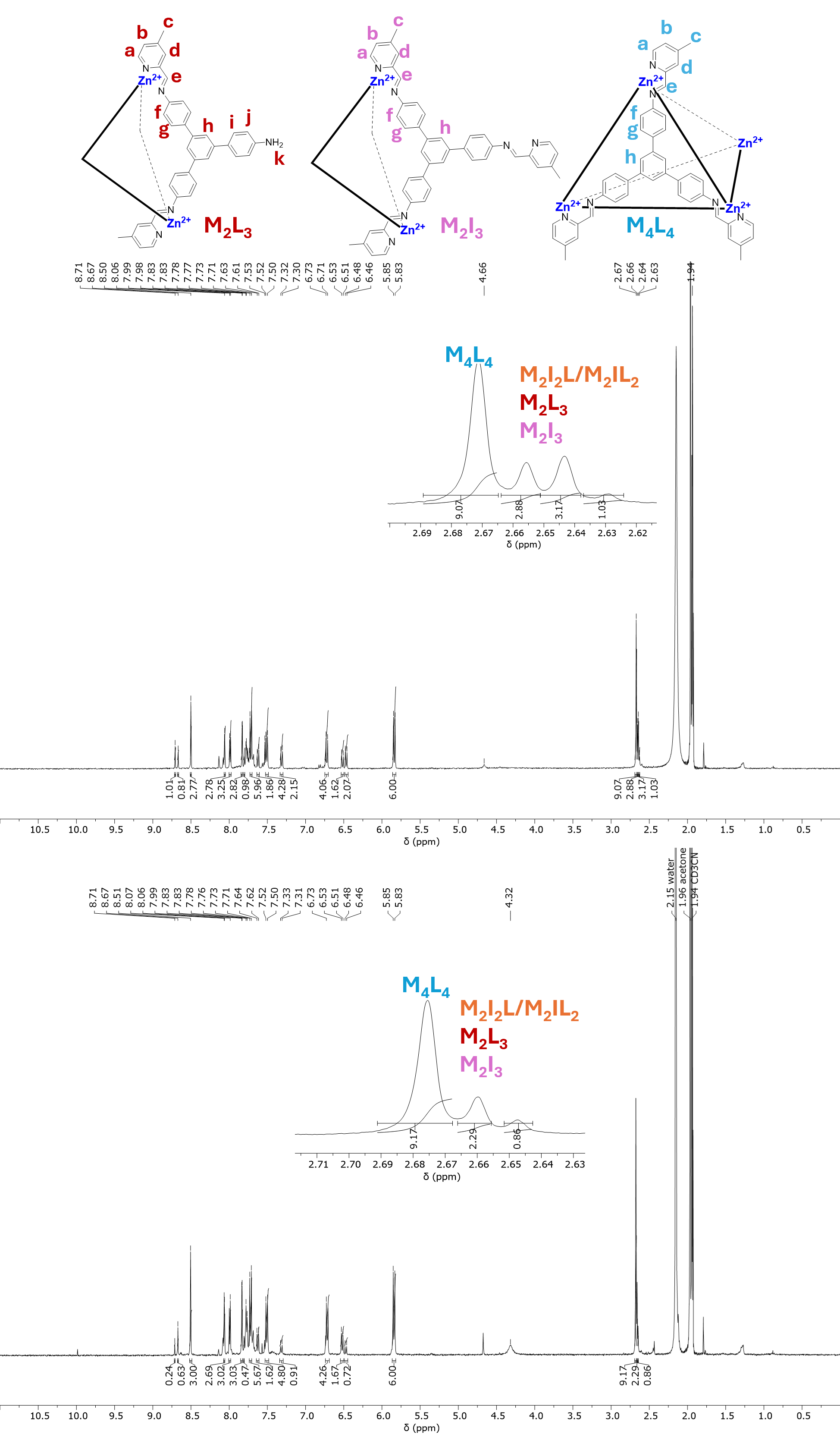


**Figure S42:** ^1^H NMR (CD_3_CN) spectrum of the reaction between triamine **A**, aldehyde **4** and metal counter ion Zn(BF_4_)_2_ for the first (top) and the second (bottom) repeat screen.


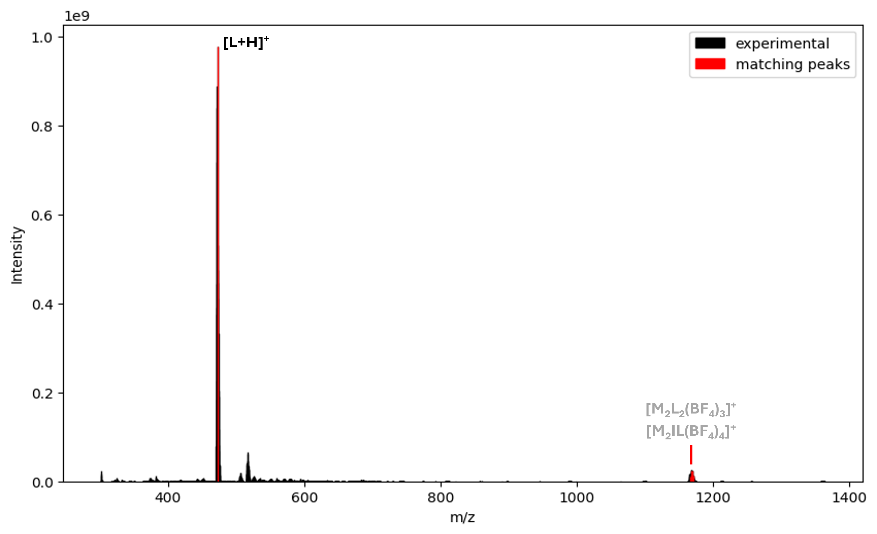

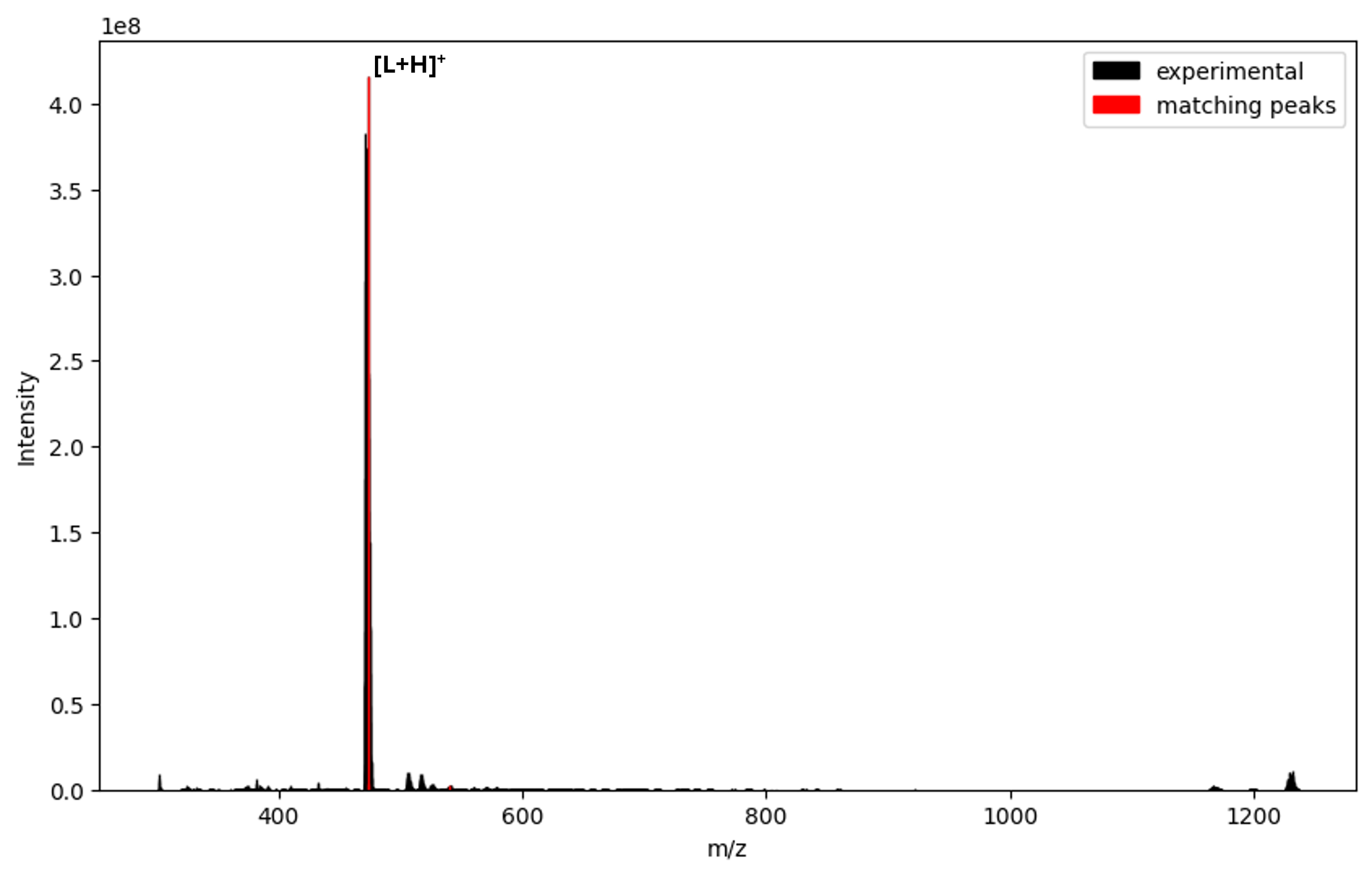


**Figure S43:** HRMS spectrum of the reaction between triamine **B**, aldehyde **1** and metal counter ion Zn(BF_4_)_2_ for the first (top) and the second (bottom) repeat screen HRMS data in black and the matching peaks from the automated HRMS analysis are identified in red. Peaks of targeted **M_N_L_N_** or **M_2_X_3_** where X = L or I are labelled in black and fragments or intermediates are labelled in grey. Charges of major peaks in HRMS spectrum that were not identified are labelled in blue.

**Figure S44:** ^1^H NMR (CD_3_CN) spectrum of the reaction between triamine **B**, aldehyde **1** and metal counter ion Zn(BF_4_)_2_ for the first (top) and the second (bottom) repeat screen


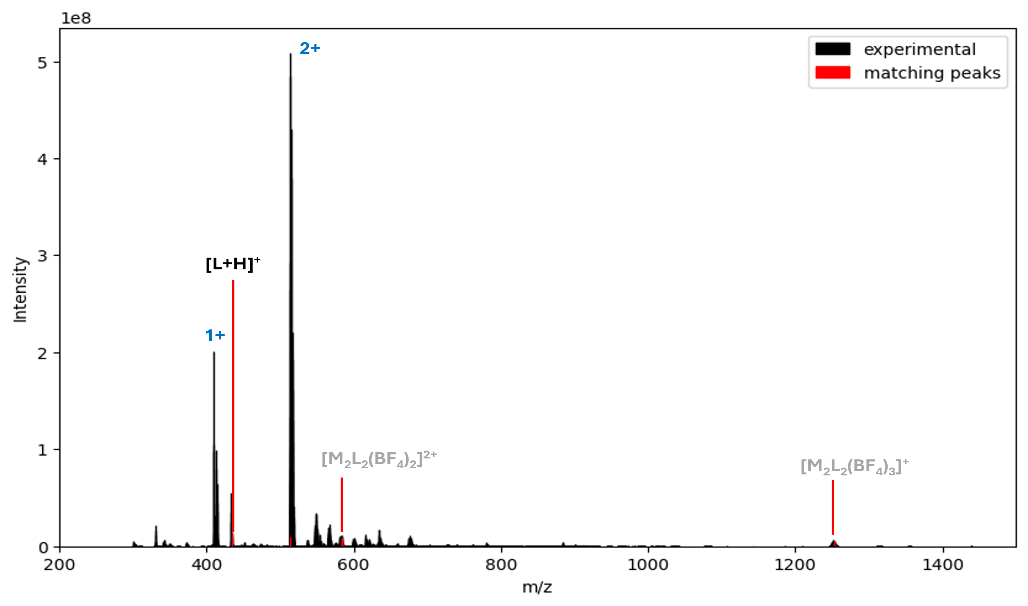

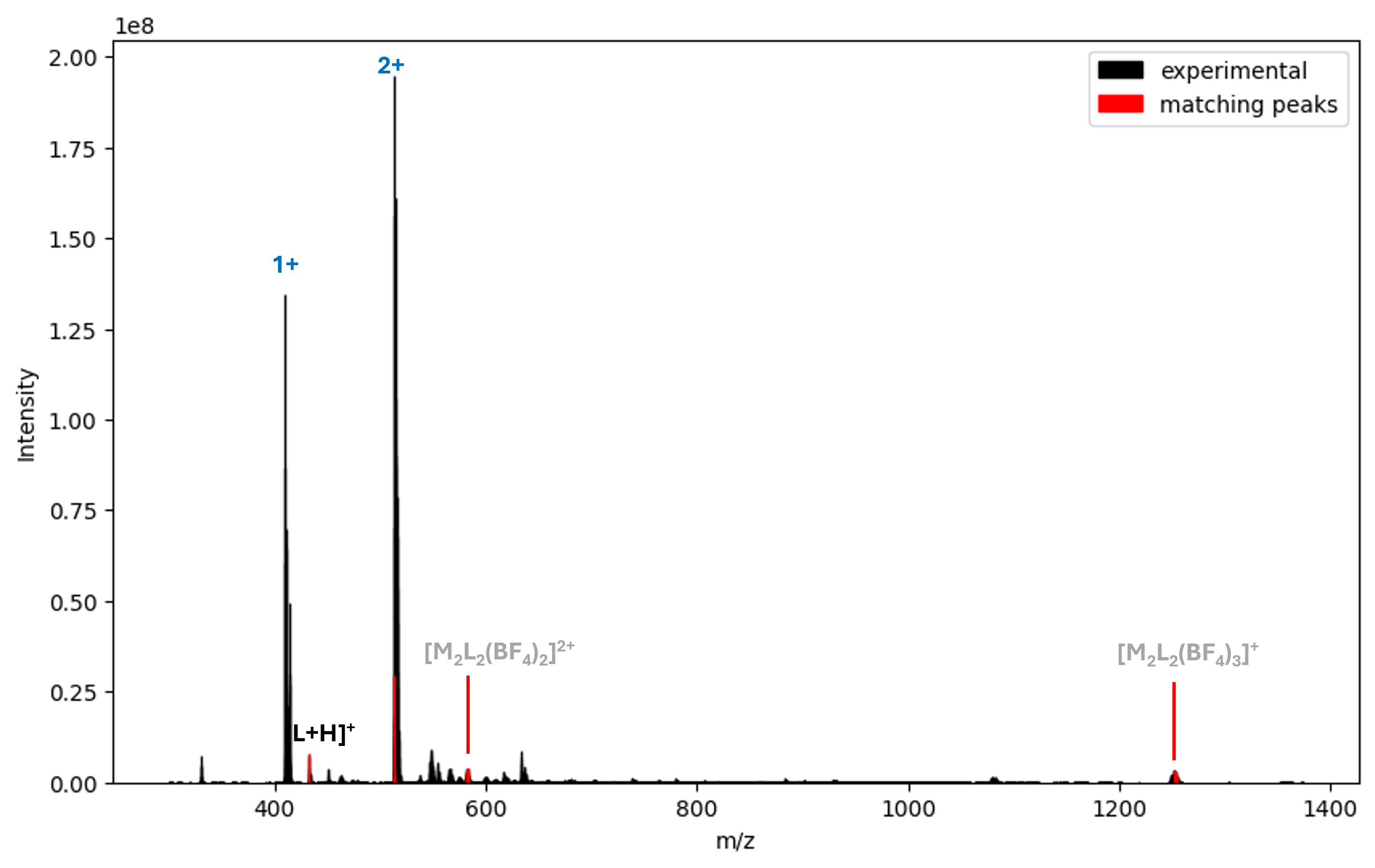


**Figure S45:** HRMS spectrum of the reaction between triamine **B**, aldehyde **2** and metal counter ion Zn(BF_4_)_2_ for the first (top) and the second (bottom) repeat screen HRMS data in black and the matching peaks from the automated HRMS analysis are identified in red. Peaks of targeted **M_N_L_N_** or **M_2_X_3_** where X = L or I are labelled in black and fragments or intermediates are labelled in grey. Charges of major peaks in HRMS spectrum that were not identified are labelled in blue.

**Figure S46:** ^1^H NMR (CD_3_CN) spectrum of the reaction between triamine **B**, aldehyde **2** and metal counter ion Zn(BF_4_)_2_ for the first (top) and the second (bottom) repeat screen.


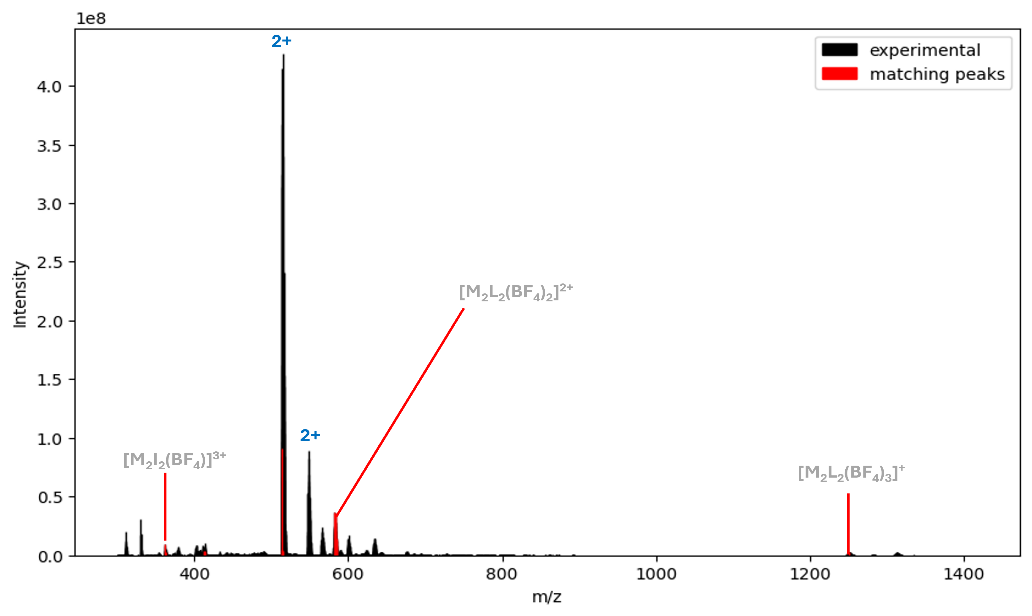

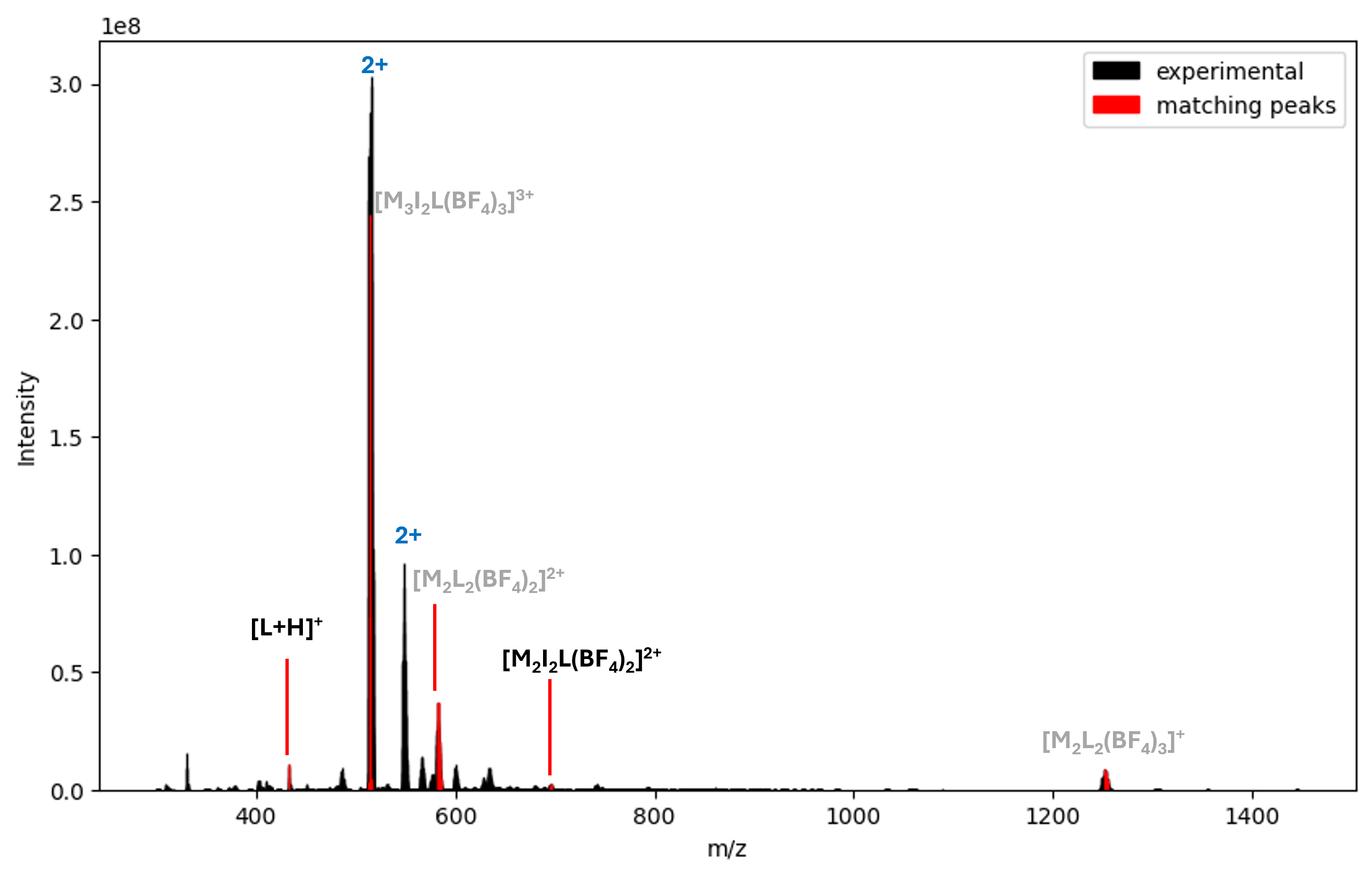


**Figure S47:** HRMS spectrum of the reaction between triamine **B**, aldehyde **3** and metal counter ion Zn(BF_4_)_2_ for the first (top) and the second (bottom) repeat screen HRMS data in black and the matching peaks from the automated HRMS analysis are identified in red. Peaks of targeted **M_N_L_N_** or **M_2_X_3_** where X = L or I are labelled in black and fragments or intermediates are labelled in grey. Charges of major peaks in HRMS spectrum that were not identified are labelled in blue.

**Figure S48:** ^1^H NMR (CD_3_CN) spectrum of the reaction between triamine **B**, aldehyde **3** and metal counter ion Zn(BF_4_)_2_ for the first (top) and the second (bottom) repeat screen.


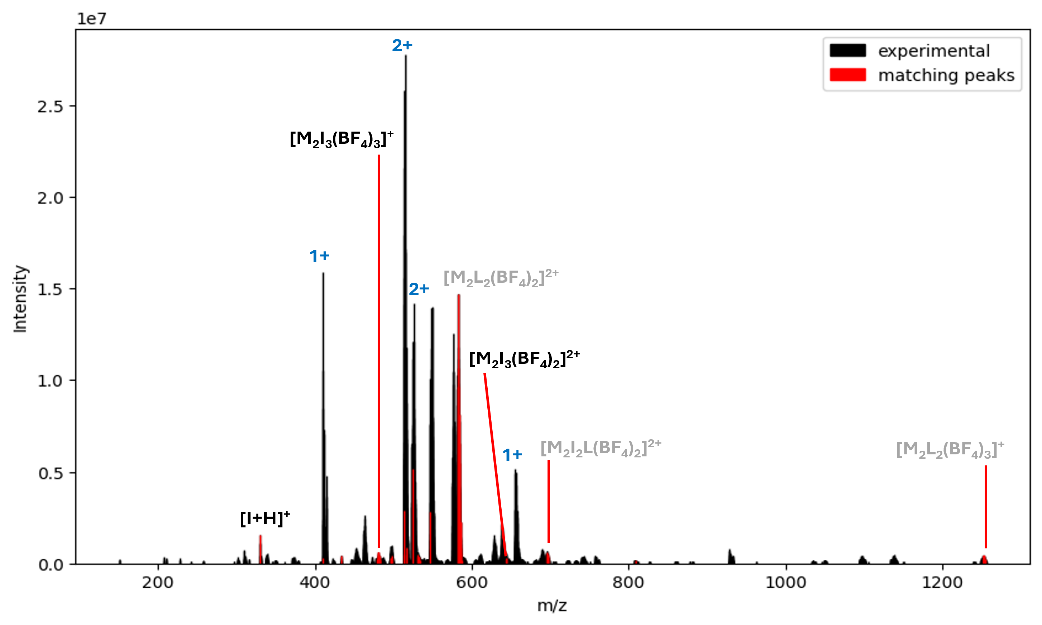

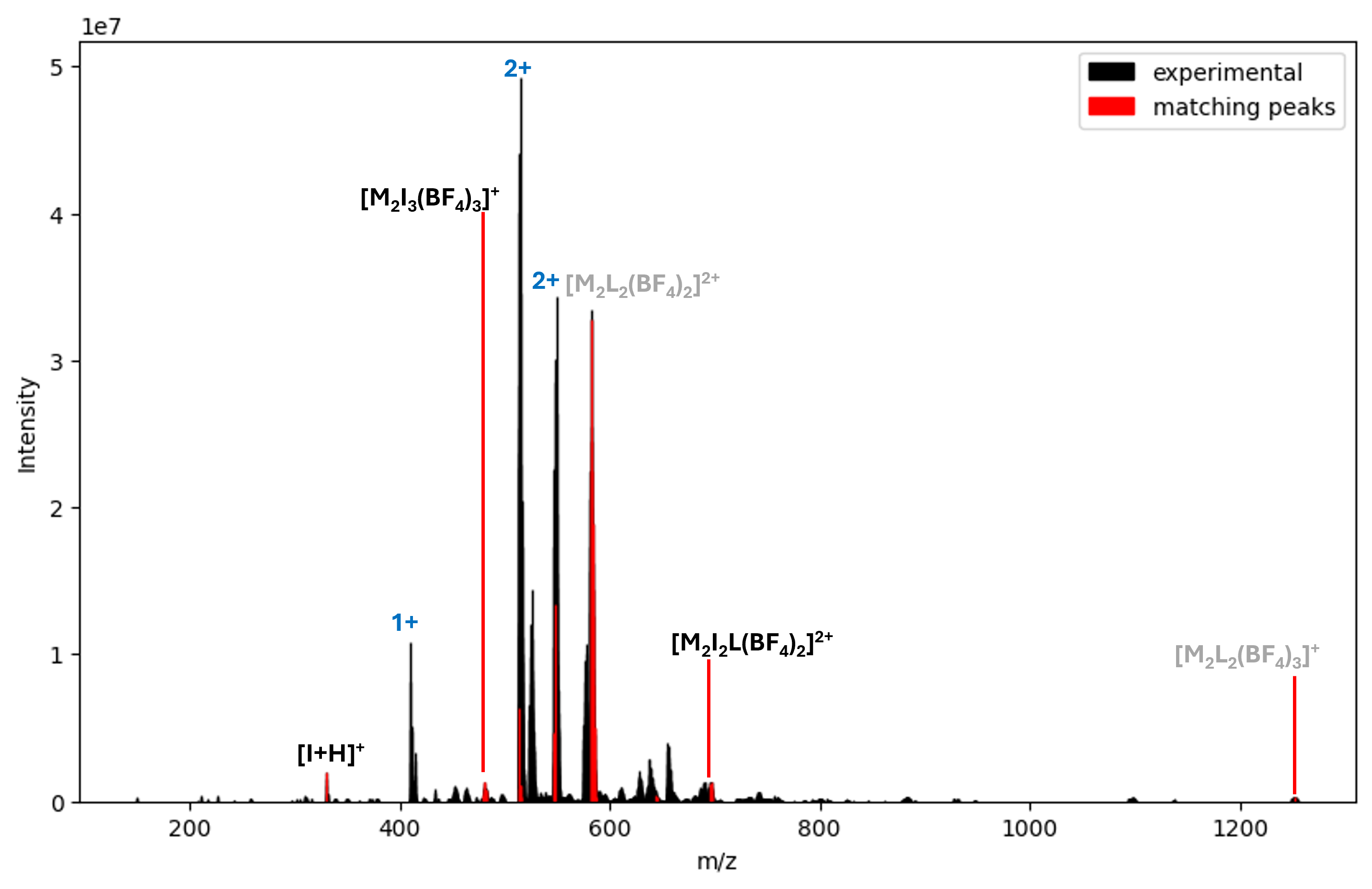


**Figure S49:** HRMS spectrum of the reaction between triamine **B**, aldehyde **4** and metal counter ion Zn(BF_4_)_2_ for the first (top) and the second (bottom) repeat screen HRMS data in black and the matching peaks from the automated HRMS analysis are identified in red. Peaks of targeted **M_N_L_N_** or **M_2_X_3_** where X = L or I are labelled in black and fragments or intermediates are labelled in grey. Charges of major peaks in HRMS spectrum that were not identified are labelled in blue.

**Figure S50:** ^1^H NMR (CD_3_CN) spectrum of the reaction between triamine **B**, aldehyde **4** and metal counter ion Zn(BF_4_)_2_ for the first (top) and the second (bottom) repeat screen.


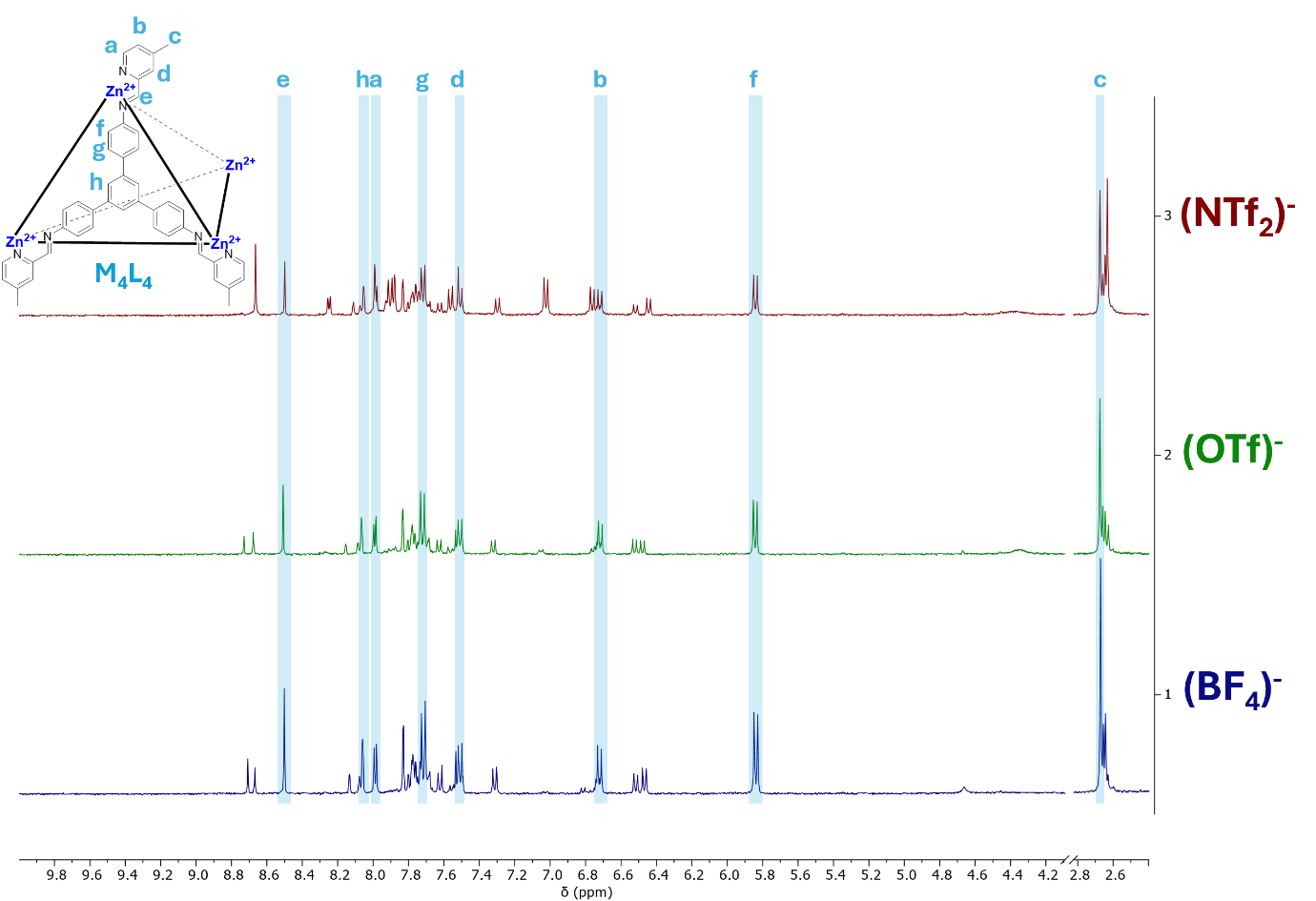


**Figure S51:** Stacked ^1^H NMR (CD_3_CN) spectra of the reaction between triamine **A**, aldehyde **4** and metal counter ions Zn(NTf_2_)_2_ (red), Zn(OTf)_2_ (green), Zn(BF_4_)_2_ (blue), with the assigned peaks for the **M_4_L_4_** tetrahedron.

# **S3. Scale up of M_4_L_4_ Metal-Organic Cages**

### **S3.1 Scale-up of Zn_4_(A1)_4_.(NTf_2_)_8_ cage 1**

To a round bottom flask equipped with stirrer bar and condenser, acetonitrile (150 mL) was added. To this solution, the triamine (**A**), aldehyde (**1**) and metal salt were added in a 4 : 12 : 4 ratio - **A** (5'-(4-aminophenyl)-[1,1':3',1''-terphenyl]-4,4''-diamine) (150 mg, 0.43 mmol), **1** picolinaldehyde (135 mg, 1.26 mmol), and **metal salt** Zn(NTf_2_)_2_ (269 mg, 0.43 mmol) - and the mixture stirred at 80 °C for 18 hours. After this time had elapsed the solution was concentrated slightly *in vacuo* and then diethyl ether added (2 x 150 mL) to produce a turbid solution. This mixture was divided into falcon tubes and spun in a centrifuge at 10,000 rpm for 10 mins. The solution was decanted off and the procedure repeated once more. The remaining solid was solubilized in MeCN and dried to yield the final product as a light yellow solid (270 mg, 0.054 mmol, 51%).

**^1^H NMR** (400 MHz, CD_3_CN) δ 8.75 (s, 3H), 8.58 – 8.40 (m, 3H), 8.32 (d, *J* = 7.7 Hz, 3H), 7.98 – 7.83 (m, 6H), 7.53 (s, 3H), 7.31 (d, *J* = 8.3 Hz, 6H), 6.46 (d, *J* = 8.3 Hz, 6H); **^13^C NMR** (101 MHz, CD_3_CN) δ 165.48, 150.51, 147.84, 147.24, 143.72, 142.98, 141.51, 132.17, 131.82, 129.39, 126.53, 123.38; **^19^F NMR** (377 MHz, CD_3_CN) δ -80.11; **ESI-MS:** *m/z*: 964.1008 [**Zn_4_(A1)_4_(NTf_2_)_4_**]^4+^, 549.4245 [**Zn_4_(A1)_4_(NTf_2_)_2_**]^6+^, 430.3766 [**Zn_4_(A1)_4_(NTf_2_)**]^7+^, in accordance with literature values.^4^

**Table S4:** The ratios of the imine N=C**H** signal integrations for the **M_4_L_4_** tetrahedron and the **M_2_X_3_** species in each mixture for the first screen, second repeat screen, and scale-up (**cage 1**) for the **A1/NTf_2_^-^** precursor combination.

|  | **M_4_L_4_ : M_2_X_3_** |
| --- | --- |
| **Screen 1** | 1 : 0.34 |
| **Screen 2** | 1 : 0.40 |
| **Scale-up** | 1 : 0.04 |

**Figure S52:** ^1^H NMR (CD_3_CN) spectrum of the scale-up of **cage 1.**

**Figure S53:** ^13^C NMR (CD_3_CN) spectrum of the scale-up of **cage 1.**

**Figure S54:** ^19^F NMR (CD_3_CN) spectrum of the scale-up of **cage 1.**

**Figure S55:** ^13^C^1^H HSQC NMR (CD_3_CN) spectrum of the scale-up of **cage 1.**

**Figure S56:** ^1^H^1^H COSY NMR (CD_3_CN) spectrum of the scale-up of **cage 1.**

**
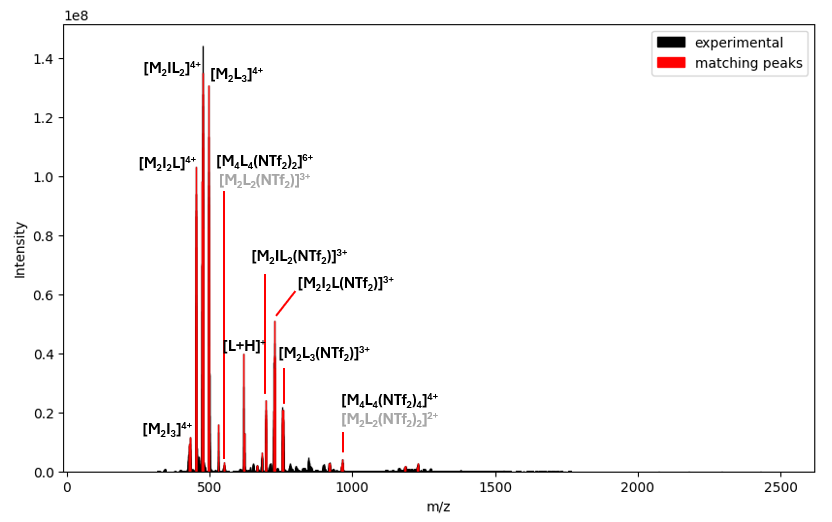
**

**Figure S57:** HRMS spectrum of the scale-up of **cage 1**

### **S3.2 Scale-up of Zn_4_(A1)_4_.(BF_4_)_8_ cage 2**

To a round bottom flask equipped with stirrer bar and condenser, acetonitrile (64 mL) was added. To this solution, the triamine (**A**), aldehyde (**3**) and metal salt were added in a 4 : 12 : 4 ratio – **A** 5'-(4-aminophenyl)-[1,1':3',1''-terphenyl]-4,4''-diamine (38 mg, 0.12 mmol), **3** picolinaldehyde (39 80mg, 0.36 mmol), and **metal salt** Zn(BF_4_)_2_ hydrate (29 mg, 0.12 mmol). The mixture was heated at 80 °C for 48 hours. After this time had elapsed the solution was concentrated slightly *in vacuo* and then diethyl ether was added (2 x 64 mL) to produce a turbid solution. This mixture was divided into falcon tubes and spun in a centrifuge at 10,000 rpm for 10 mins. The solution was decanted off and the procedure repeated three times, before the remaining solid was solubilized in MeCN and dried to yield the final product as a yellowish brown solid (60 mg, 0.012 mmol, 60%).

**^1^H NMR** (400 MHz, CD_3_CN) δ 8.80 (s, 3H), 8.52 – 8.45 (m, 3H), 8.34 (d, *J* = 7.7 Hz, 3H), 7.93 – 7.87 (m, 6H), 7.55 (s, 3H), 7.33 (d, *J* = 8.2 Hz, 6H), 6.50 (d, *J* = 8.2 Hz, 6H); **^13^C NMR** (101 MHz, CD_3_CN) δ 165.57, 150.48, 147.83, 147.33, 143.70, 142.99, 141.50, 136.83, 136.74, 132.19, 131.77, 129.40, 126.53, 123.44;**^19^F NMR** (377 MHz, CD_3_CN) δ -151.18, -151.24; **ESI-MS:** *m/z*: 497.153 [**Zn_2_(A1)_3_**]^4+^, 475.8974 [**Zn_4_(A1)_3_(BF_4_)_3_**]^5+^ fragment ions.

**Table S5:** The ratios of the imine N=C**H** signal integrations for the **M_4_L_4_** tetrahedron and the **M_2_X_3_** species in each mixture for the first screen, second repeat screen, and scale-up (**cage 2**) for the **A1/BF_4_^-^** precursor combination.

|  | **M_4_L_4_ : M_2_X_3_** |
| --- | --- |
| **Screen 1** | 1 : 0.26 |
| **Screen 2** | 1 : 0.14 |
| **Scale-up** | 1 : 0.16 |

**Figure S58:** ^1^H NMR (CD_3_CN) spectrum of the scale-up of **cage 2.**

**Figure S59:** ^13^C NMR (CD_3_CN) spectrum of the scale-up of **cage 2.**

**Figure S60:** ^19^F NMR (CD_3_CN) spectrum of the scale-up of **cage 2.**

**Figure S61:** ^13^C^1^H HSQC NMR (CD_3_CN) spectrum of the scale-up of **cage 2.**

**Figure S62:** ^1^H^1^H COSY NMR (CD_3_CN) spectrum of the scale-up of **cage 2.**

**
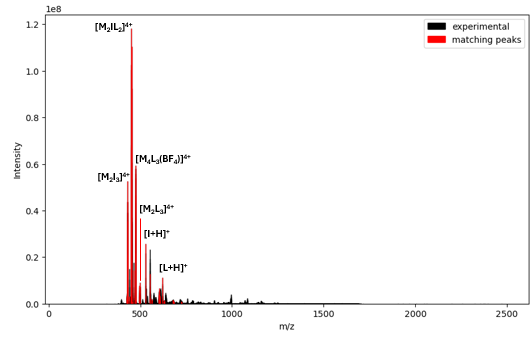
**

**Figure S63:** HRMS spectrum of the scale-up of **cage 2.**

### **S3.3 Scale-up of Zn_4_(A3)_4_.(BF_4_)_8_ cage 3**

To a round bottom flask equipped with stirrer bar and condenser, acetonitrile (64 mL) was added. To this solution, the triamine (**A**), aldehyde (**3**) and metal salt were added in a 4 : 12 : 4 ratio – **A** 5'-(4-aminophenyl)-[1,1':3',1''-terphenyl]-4,4''-diamine (38 mg, 0.12 mmol), **3** 5-methylpicolinaldehyde (44 mg, 0.36 mmol), and **metal salt** Zn(BF_4_)_2_ hydrate (29 mg, 0.12 mmol). The mixture was heated at 80 °C for 48 hours. After this time had elapsed diethyl ether (128 mL) was added to the solution to produce a turbid solution. This mixture was divided into falcon tubes and spun in a centrifuge at 10,000 rpm for 10 mins. The solution was decanted off and the procedure repeated three times and then the remaining solid solubilized in MeCN and dried to yield the final product as a yellowish brown solid (50 mg, 0.014 mmol, 16%).

**^1^H NMR** (400 MHz, CD_3_CN) δ 8.72 (s, 3H), 8.24 (dd, *J* = 26.2, 8.0 Hz, 6H), 7.77 (s, 3H), 7.51 (s, 3H), 7.30 (d, *J* = 8.2 Hz, 6H), 6.42 (d, *J* = 8.2 Hz, 6H), 2.45 (s, 9H); **^13^C NMR** (101 MHz, CD_3_CN) δ 190.35, 165.23, 151.18, 148.02, 144.97, 143.50, 143.32, 143.09, 141.37, 131.49, 129.39, 126.53, 123.36, 19.21; **^19^F NMR** (377 MHz, CD_3_CN) δ -151.15, -151.20; **ESI-MS:** *m/z*: 709.9429 [**Zn_4_(I)_4_(BF_4_)_4_**]^4+^, 507.1682 [**Zn_4_(I)(A3)_4_(BF_4_)**]^7+^, 477.012 [**Zn_2_(A3)_3_**]^4+^, fragment ions.

**Table S6:** The ratios of the imine N=C**H** signal integrations for the **M_4_L_4_** tetrahedron and the **M_2_X_3_** species in each mixture for the first screen, second repeat screen, and scale-up (**cage 3**) for the **A3/BF_4_^-^** precursor combination.

|  | **M_4_L_4_ : M_2_X_3_** |
| --- | --- |
| **Screen 1** | 1 : 0.16 |
| **Screen 2** | 1 : 0.08 |
| **Scale-up** | 1 : 0.19 |

**Figure S64:** ^1^H NMR (CD_3_CN) spectrum of the scale-up of **cage 3.**

**Figure S65:** ^13^C NMR (CD_3_CN) spectrum of the scale-up of **cage 3.**

**Figure S66:** ^19^F NMR (CD_3_CN) spectrum of the scale-up of **cage 3.**

**Figure S67:** ^13^C^1^H HSQC NMR (CD_3_CN) spectrum of the scale-up of **cage 3.**

**Figure S68:** ^1^H^1^H COSY NMR (CD_3_CN) spectrum of the scale-up of **cage 3.**


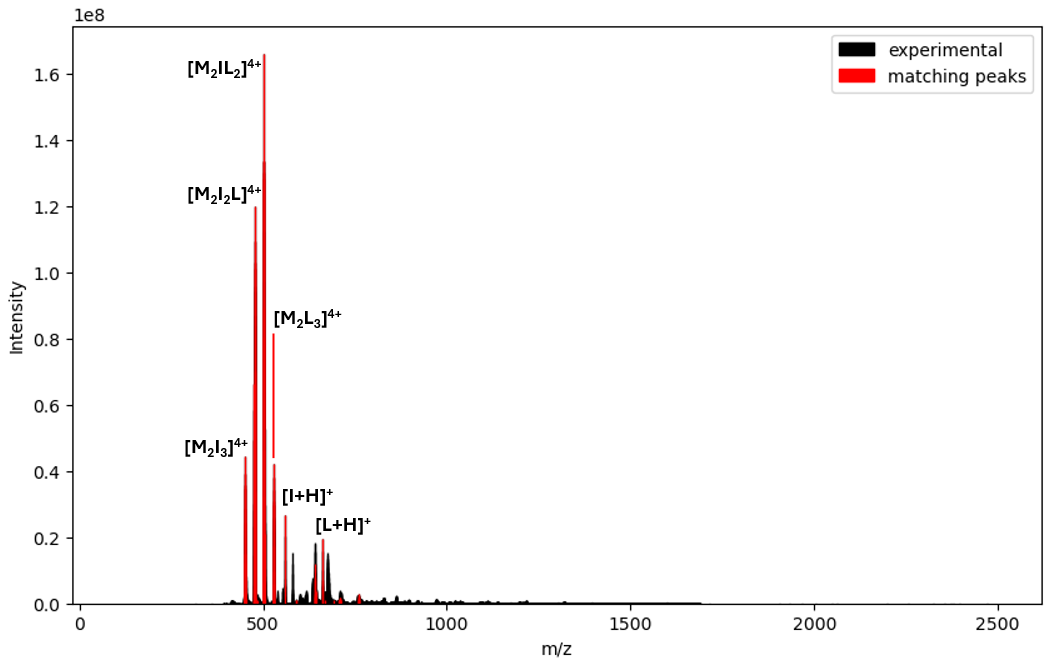


**Figure S69:** HRMS spectrum of the scale-up of **cage 3.**

### **S3.4. Diffusion NMR**

All measurements were carried out using a 5 mm indirect detection probe equipped with z-gradient coil producing a nominal maximum gradient of 45 G/cm. Diffusion data was collected using the Bruker longitudinal eddy current delay (LED) bipolar gradient pulse sequence (ledbpgp2s). A diffusion encoding pulse (δ) of length 3.2 or 4 ms and a diffusion delay (Δ) 0.02 s (298 K) was used. Gradient amplitudes were equally spaced between 0.869 G/cm and 41.271 G/cm. Each FID was acquired using 32k data points. All experiments were carried out at a nominal probe temperature of 298 K or 353 K, with and air flow of 600 m³ min⁻¹.

Diffusion coefficients were calculated from signal intensities using the Skejskal-Tanner equation using Bruker Dynamics Center:

$$I=I_{0}e^{-\gamma^{2}G^{2}\delta^{2}\left( \Delta-\frac{\delta}{3} \right)D}$$

Where I is the signal intensity, I_0_ is the signal intensity at a gradient strength of zero, G is the gradient strength, and D is the diffusion coefficient, ɣ is the gyromagnetic ratio, δ is the gradient pulse length and Δ is the diffusion time.

**S3.4.1 Diffusion NMR of cage 1**


**Figure S70**: Pseudo 2D DOSY spectrum of **cage 1** at 298 K

**Table S7**: Chemical shifts and diffusion coefficients of peaks for the species in **cage 1** at 298 K

| **Species** | **Chemical Shift (ppm)** | **Diffusion Coefficient (m^2^/s)** | **Average Diffusion Coefficient (m^2^/s)** |
| --- | --- | --- | --- |
| A | 8.74 | 1.05 × 10⁻⁹ | 1.05 × 10⁻⁹ ± 7.6 × 10⁻¹² ^a^ |
|  | 8.49 | 1.06 × 10⁻⁹ |  |
|  | 8.49 | 1.05 × 10⁻⁹ |  |
|  | 8.48 | 1.05 × 10⁻⁹ |  |
|  | 8.47 | 1.05 × 10⁻⁹ |  |
|  | 8.47 | 1.05 × 10⁻⁹ |  |
|  | 8.46 | 1.05 × 10⁻⁹ |  |
|  | 8.46 | 1.05 × 10⁻⁹ |  |
|  | 7.90 | 1.05 × 10⁻⁹ |  |
|  | 7.89 | 1.05 × 10⁻⁹ |  |
|  | 7.89 | 1.06 × 10⁻⁹ |  |
|  | 7.52 | 1.07 × 10⁻⁹ |  |
|  | 7.31 | 1.06 × 10⁻⁹ |  |
|  | 7.29 | 1.07 × 10⁻⁹ |  |
|  | 6.46 | 1.07 × 10⁻⁹ |  |
|  | 6.45 | 1.07 × 10⁻⁹ |  |

^a^Error shown is standard deviation of diffusion coefficients for species.


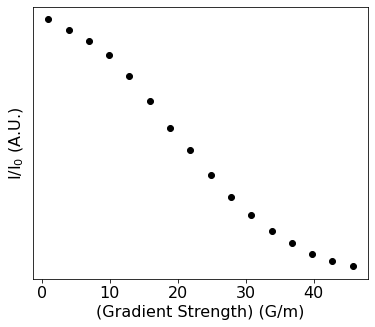

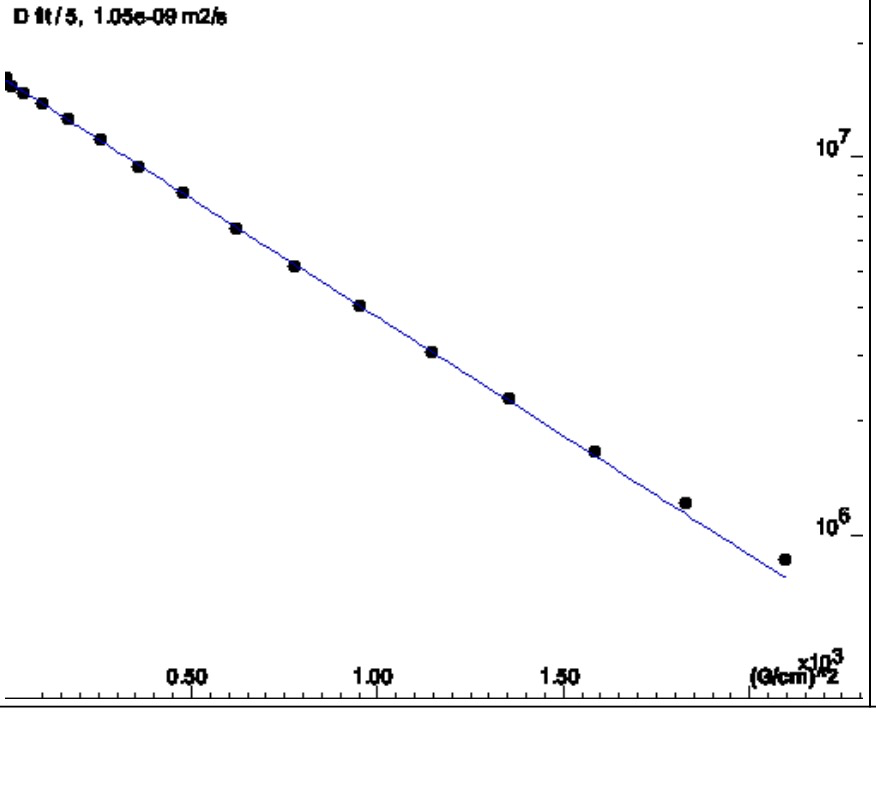


**Figure S71**: Representative diffusion decay curve (left) and fit (right) for species A in **cage 1** at 298 K

**S3.4.2 Diffusion NMR of cage 2**

**Figure S72**: Pseudo 2D DOSY spectrum of **cage 2** at 298 K.

**Table S8**: Chemical shifts and diffusion coefficients of peaks for each species in **cage 2** at 298 K.

| **Species** | **Chemical Shift (ppm)** | **Diffusion Coefficient (m^2^/s)** | **Average Diffusion Coefficient (m^2^/s)** |
| --- | --- | --- | --- |
| A | 8.79 | 7.00 × 10⁻¹⁰ | 7.05 × 10⁻¹⁰ ± 4.3 × 10⁻¹² ^a^ |
|  | 8.47 | 7.16 × 10⁻¹⁰ |  |
|  | 8.34 | 7.04 × 10⁻¹⁰ |  |
|  | 8.32 | 7.06 × 10⁻¹⁰ |  |
|  | 7.90 | 7.02 × 10⁻¹⁰ |  |
|  | 7.89 | 7.03 × 10⁻¹⁰ |  |
|  | 7.54 | 7.02 × 10⁻¹⁰ |  |
|  | 7.33 | 7.02 × 10⁻¹⁰ |  |
|  | 7.32 | 7.06 × 10⁻¹⁰ |  |
|  | 6.50 | 7.07 × 10⁻¹⁰ |  |
|  | 6.48 | 7.09 × 10⁻¹⁰ |  |
| B | 8.58 | 8.29 × 10⁻¹⁰ | 8.06 × 10⁻¹⁰ ± 2.0 × 10⁻¹¹ ^a^ |
|  | 8.27 | 8.14 × 10⁻¹⁰ |  |
|  | 8.26 | 8.06 × 10⁻¹⁰ |  |
|  | 8.16 | 8.10 × 10⁻¹⁰ |  |
|  | 8.15 | 7.94 × 10⁻¹⁰ |  |
|  | 7.83 | 8.09 × 10⁻¹⁰ |  |
|  | 7.73 | 7.96 × 10⁻¹⁰ |  |
|  | 7.72 | 8.01 × 10⁻¹⁰ |  |
|  | 7.51 | 7.71 × 10⁻¹⁰ |  |
|  | 7.49 | 7.75 × 10⁻¹⁰ |  |
|  | 6.71 | 8.41 × 10⁻¹⁰ |  |
|  | 6.70 | 8.24 × 10⁻¹⁰ |  |

^a^Error shown is standard deviation of diffusion coefficients for species.


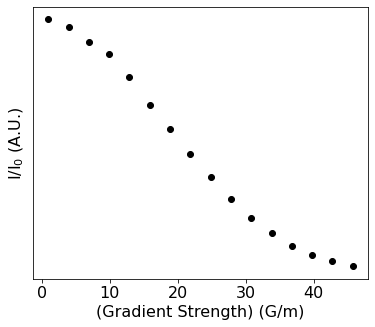

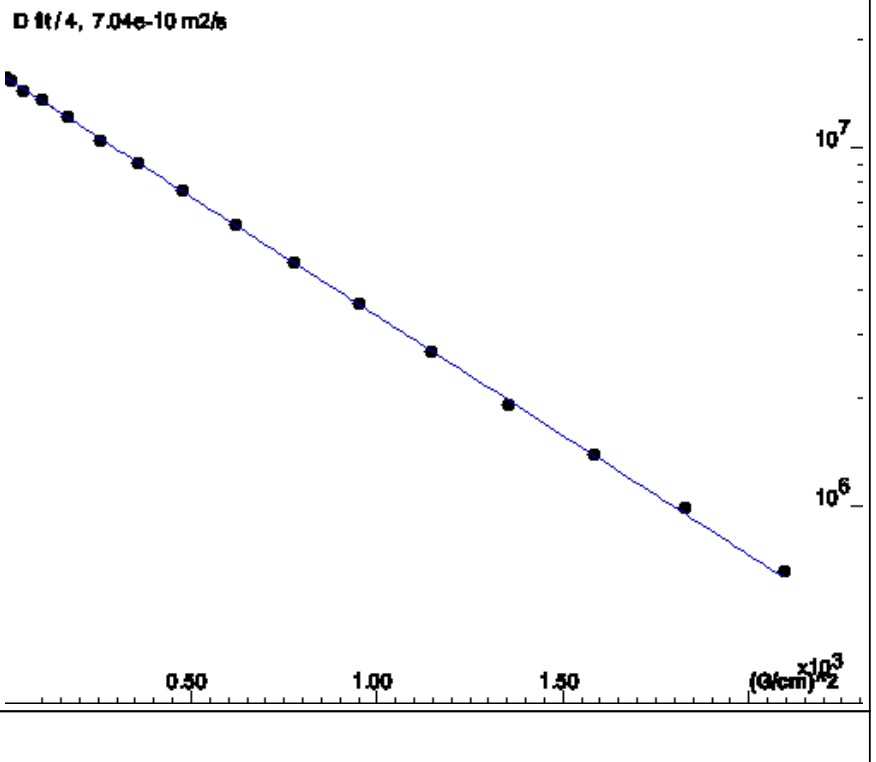


**Figure S73**: Representative diffusion decay curve (left) and fit (right) for species A in **cage 2** at 298 K.


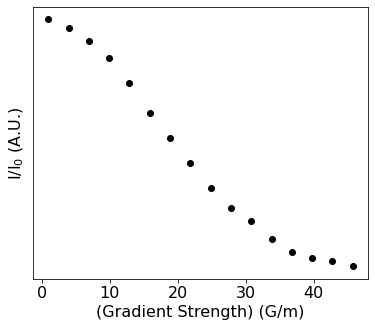

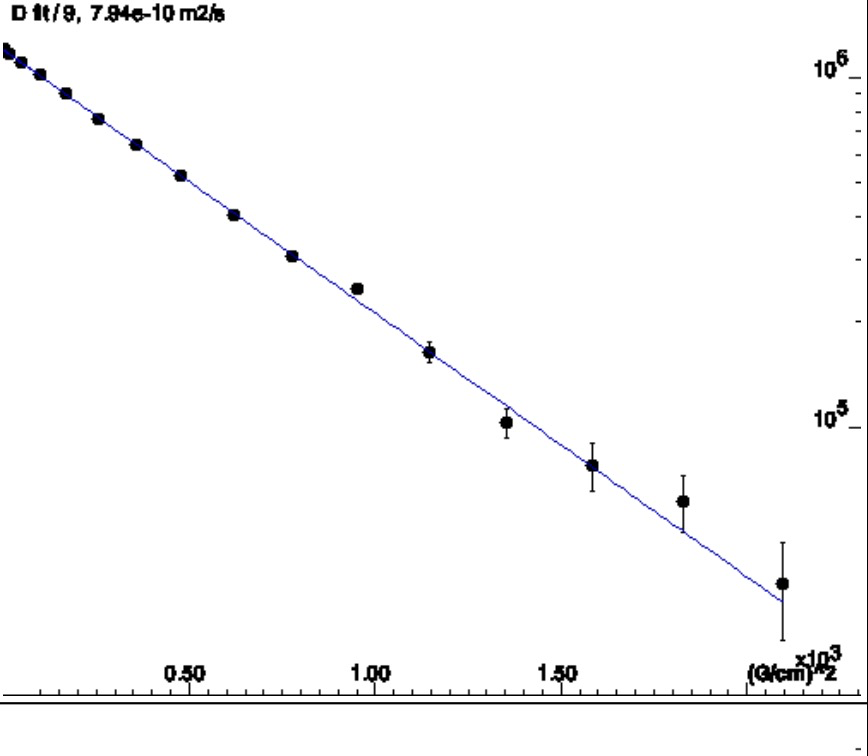


**Figure S74**: Representative diffusion decay curve (left) and fit (right) for species B in **cage 2** at 298 K.

**S3.4.3 Diffusion NMR of cage 3**


**Figure S75**: Pseudo 2D DOSY spectrum of **cage 3** at 298 K.

**Table S9**: Chemical shifts and diffusion coefficients of peaks for each species in **cage 3** at 298 K.

| **Species** | **Chemical Shift (ppm)** | **Diffusion Coefficient (m^2^/s)** | **Average Diffusion Coefficient (m^2^/s)** |
| --- | --- | --- | --- |
| A | 8.72 | 7.65 × 10⁻¹⁰ | 7.60 × 10⁻¹⁰ ± 9.6 × 10⁻¹² ^a^ |
|  | 8.27 | 7.47 × 10⁻¹⁰ |  |
|  | 8.25 | 7.52 × 10⁻¹⁰ |  |
|  | 8.21 | 7.58 × 10⁻¹⁰ |  |
|  | 8.19 | 7.63 × 10⁻¹⁰ |  |
|  | 7.76 | 7.53 × 10⁻¹⁰ |  |
|  | 7.50 | 7.87 × 10⁻¹⁰ |  |
|  | 7.30 | 7.58 × 10⁻¹⁰ |  |
|  | 7.28 | 7.57 × 10⁻¹⁰ |  |
|  | 6.42 | 7.64 × 10⁻¹⁰ |  |
|  | 6.40 | 7.56 × 10⁻¹⁰ |  |
|  | 2.45 | 7.63 × 10⁻¹⁰ |  |
| B | 8.51 | 8.89 × 10⁻¹⁰ | 8.85 × 10⁻¹⁰ ± 1.2 × 10⁻¹¹ ^a^ |
|  | 8.14 | 8.96 × 10⁻¹⁰ |  |
|  | 8.01 | 8.66 × 10⁻¹⁰ |  |
|  | 7.82 | 8.67 × 10⁻¹⁰ |  |
|  | 7.72 | 8.87 × 10⁻¹⁰ |  |
|  | 7.70 | 8.99 × 10⁻¹⁰ |  |
|  | 5.80 | 8.98 × 10⁻¹⁰ |  |
|  | 2.51 | 8.81 × 10⁻¹⁰ |  |

^a^Error shown is standard deviation of diffusion coefficients for species.


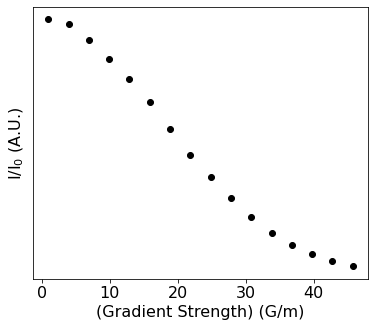

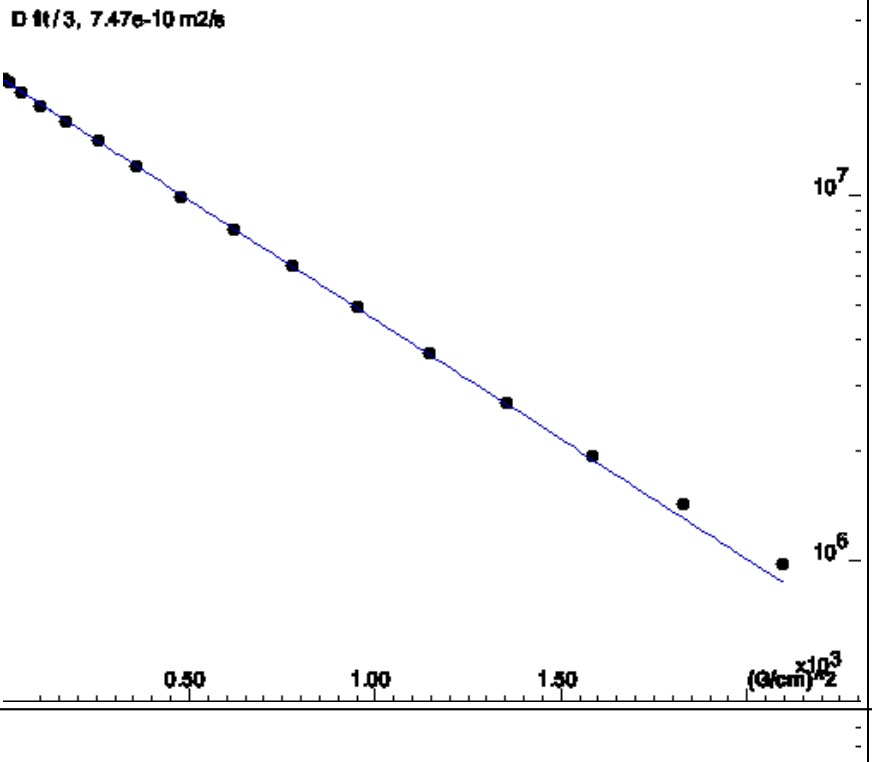


**Figure S76**: Representative diffusion decay curve (left) and fit (right) for species A in **cage 3** at 298 K.


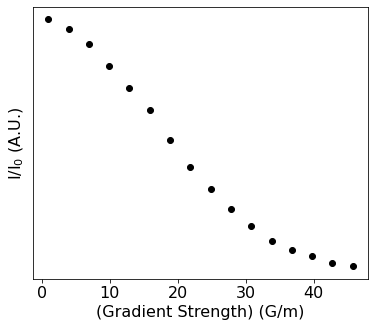

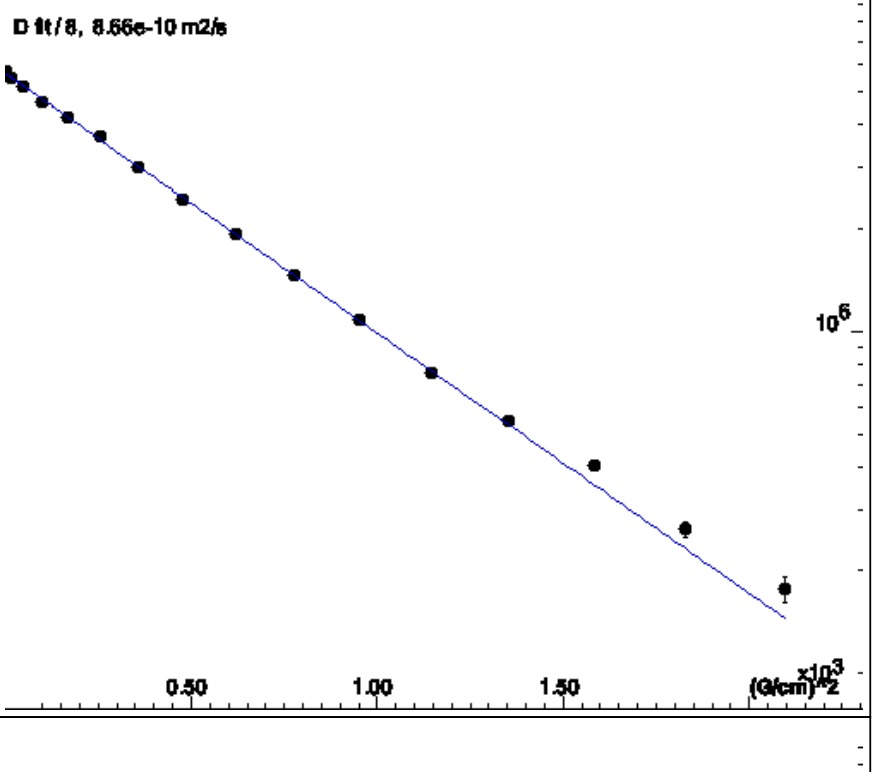


**Figure S77**: Representative diffusion decay curve (left) and fit (right) for species B in **cage 3** at 298 K.

# **S4 Automated Screen for Effect of Precursor Stoichiometry and Concentration Study**

### **S4.1 Automated Synthesis**

**
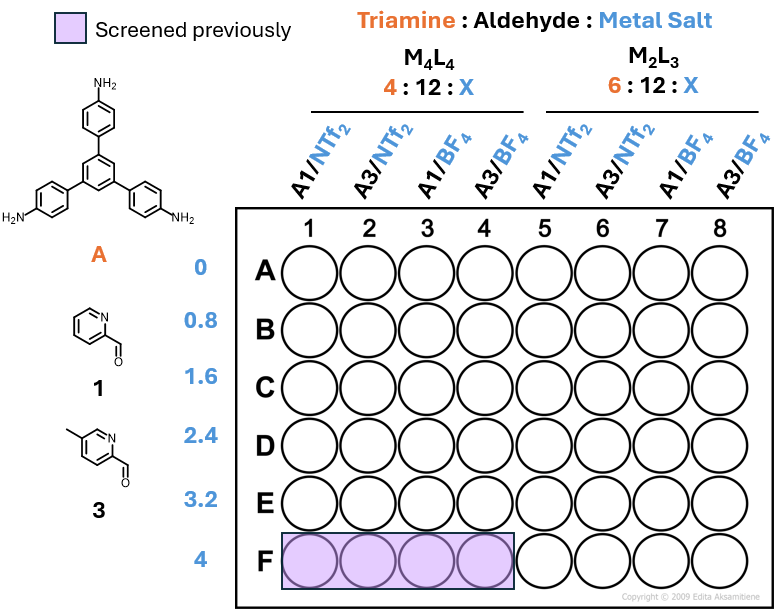
**

**Figure S78:** Plate format used for the automated screening of precursor ratio to target the **M_4_L_4_** tetrahedron (4:12:n) and **M_2_L_3_** helicate (6:12:n), changing the metal salt concentration (n) between 0-100% (n= 0, 0.8, 1.6, 2.4, 3.2, 4). Precursor combinations included **A1/NTf_2_^-^**, **A3/NTf_2_**, **A1/BF_4_^-^** **and A3/BF_4_ ^-^**.

**Automated method:** Performed on the Opentrons OT-2 (robot v7.2.1 and 7.2.1 app version) with the same settings as the automated synthesis screen. Precursor ratios of triamine:aldehyde:metal salt were selected to target the **M_4_L_4_** tetrahedron (4:12:n) and helicate (6:12:n), changing the metal salt concentration (n) between 0-100% (n= 0, 0.8, 1.6, 2.4, 3.2, 4). Stock solutions of precursors **A**, **1**, **3**, **Zn(NTf_2_)_2_** and **Zn(BF_4_)_2_** were made up at 5 mg/mL in acetonitrile and placed in 24-well 8 mL plates and acetonitrile places in a 6-well 25 mL plate. Following this the solvent was removed using the EquaVAP and dissolved in 500 μL of acetonitrile-*d_3_*, transferred to NMR tubes by the OT-2 in a 3D printed 96-well NMR tube holder plate and ^1^H NMR analysis was undertaken. The code required to replicate the protocol is available at https://github.com/GreenawayLab/development-automated-workflow-mocs.git. All the ^1^H NMR raw data can be found on Zenodo at <https://doi.org/10.5281/zenodo.14183035>.

**Table S10:** Stock solution and solvent volumes used in each reaction on the Opentrons OT-2 platform for the automated screen exploring the two ratios of triamine:aldehyde:metal salt and varying metal salt concentration, totalling reaction volumes of 1 mL.

| **Vial** | **Triamine** | **Amount of triamine (mmol)** | **Volume triamine stock solution (mL)** | **Aldehyde** | **Amount of aldehyde (mmol)** | **Volume of aldehyde (mL)** | **Counter ion** | **Amount of counter ion (mmol)** | **Volume of counter ion (mL)** | **Volume of CH_3_CN**  **top-up (total = 1 mL)** |
| --- | --- | --- | --- | --- | --- | --- | --- | --- | --- | --- |
| A1 | **A** | 0.0019 | 0.133 | **1** | 0.0057 | 0.122 | **Zn(NTf_2_)_2_** | 0 | 0.238 | 0.745 |
| A2 | **A** | 0.0019 | 0.133 | **3** | 0.0057 | 0.138 | **Zn(BF_4_)_2_** | 0 | 0.238 | 0.729 |
| A3 | **A** | 0.0019 | 0.133 | **1** | 0.0057 | 0.122 | **Zn(NTf_2_)_2_** | 0 | 0.238 | 0.745 |
| A4 | **A** | 0.0019 | 0.133 | **3** | 0.0057 | 0.138 | **Zn(BF_4_)_2_** | 0 | 0.238 | 0.729 |
| A5 | **A** | 0.0029 | 0.200 | **1** | 0.0057 | 0.122 | **Zn(NTf_2_)_2_** | 0 | 0.238 | 0.678 |
| A6 | **A** | 0.0029 | 0.200 | **3** | 0.0057 | 0.138 | **Zn(BF_4_)_2_** | 0 | 0.238 | 0.662 |
| A7 | **A** | 0.0029 | 0.200 | **1** | 0.0057 | 0.122 | **Zn(NTf_2_)_2_** | 0 | 0.238 | 0.678 |
| A8 | **A** | 0.0029 | 0.200 | **3** | 0.0057 | 0.138 | **Zn(BF_4_)_2_** | 0 | 0.238 | 0.662 |
| B1 | **A** | 0.0019 | 0.133 | **1** | 0.0057 | 0.122 | **Zn(NTf_2_)_2_** | 0.0004 | 0.047 | 0.698 |
| B2 | **A** | 0.0019 | 0.133 | **3** | 0.0057 | 0.138 | **Zn(BF_4_)_2_** | 0.0004 | 0.047 | 0.682 |
| B3 | **A** | 0.0019 | 0.133 | **1** | 0.0057 | 0.122 | **Zn(NTf_2_)_2_** | 0.0004 | 0.026 | 0.719 |
| B4 | **A** | 0.0019 | 0.133 | **3** | 0.0057 | 0.138 | **Zn(BF_4_)_2_** | 0.0004 | 0.026 | 0.703 |
| B5 | **A** | 0.0029 | 0.200 | **1** | 0.0057 | 0.122 | **Zn(NTf_2_)_2_** | 0.0004 | 0.047 | 0.631 |
| B6 | **A** | 0.0029 | 0.200 | **3** | 0.0057 | 0.138 | **Zn(BF_4_)_2_** | 0.0004 | 0.047 | 0.615 |
| B7 | **A** | 0.0029 | 0.200 | **1** | 0.0057 | 0.122 | **Zn(NTf_2_)_2_** | 0.0004 | 0.026 | 0.652 |
| B8 | **A** | 0.0029 | 0.200 | **3** | 0.0057 | 0.138 | **Zn(BF_4_)_2_** | 0.0004 | 0.026 | 0.636 |
| C1 | **A** | 0.0019 | 0.133 | **1** | 0.0057 | 0.122 | **Zn(NTf_2_)_2_** | 0.0008 | 0.094 | 0.651 |
| C2 | **A** | 0.0019 | 0.133 | **3** | 0.0057 | 0.138 | **Zn(BF_4_)_2_** | 0.0008 | 0.094 | 0.635 |
| C3 | **A** | 0.0019 | 0.133 | **1** | 0.0057 | 0.122 | **Zn(NTf_2_)_2_** | 0.0008 | 0.052 | 0.693 |
| C4 | **A** | 0.0019 | 0.133 | **3** | 0.0057 | 0.138 | **Zn(BF_4_)_2_** | 0.0008 | 0.052 | 0.677 |
| C5 | **A** | 0.0029 | 0.200 | **1** | 0.0057 | 0.122 | **Zn(NTf_2_)_2_** | 0.0008 | 0.094 | 0.584 |
| C6 | **A** | 0.0029 | 0.200 | **3** | 0.0057 | 0.138 | **Zn(BF_4_)_2_** | 0.0008 | 0.094 | 0.568 |
| C7 | **A** | 0.0029 | 0.200 | **1** | 0.0057 | 0.122 | **Zn(NTf_2_)_2_** | 0.0008 | 0.052 | 0.626 |
| C8 | **A** | 0.0029 | 0.200 | **3** | 0.0057 | 0.138 | **Zn(BF_4_)_2_** | 0.0008 | 0.052 | 0.610 |
| D1 | **A** | 0.0019 | 0.133 | **1** | 0.0057 | 0.122 | **Zn(NTf_2_)_2_** | 0.0011 | 0.142 | 0.603 |
| D2 | **A** | 0.0019 | 0.133 | **3** | 0.0057 | 0.138 | **Zn(BF_4_)_2_** | 0.0011 | 0.142 | 0.587 |
| D3 | **A** | 0.0019 | 0.133 | **1** | 0.0057 | 0.122 | **Zn(NTf_2_)_2_** | 0.0011 | 0.078 | 0.667 |
| D4 | **A** | 0.0019 | 0.133 | **3** | 0.0057 | 0.138 | **Zn(BF_4_)_2_** | 0.0011 | 0.078 | 0.651 |
| D5 | **A** | 0.0029 | 0.200 | **1** | 0.0057 | 0.122 | **Zn(NTf_2_)_2_** | 0.0011 | 0.142 | 0.536 |
| D6 | **A** | 0.0029 | 0.200 | **3** | 0.0057 | 0.138 | **Zn(BF_4_)_2_** | 0.0011 | 0.142 | 0.520 |
| D7 | **A** | 0.0029 | 0.200 | **1** | 0.0057 | 0.122 | **Zn(NTf_2_)_2_** | 0.0011 | 0.078 | 0.600 |
| D8 | **A** | 0.0029 | 0.200 | **3** | 0.0057 | 0.138 | **Zn(BF_4_)_2_** | 0.0011 | 0.078 | 0.584 |
| E1 | **A** | 0.0019 | 0.133 | **1** | 0.0057 | 0.122 | **Zn(NTf_2_)_2_** | 0.0015 | 0.189 | 0.556 |
| E2 | **A** | 0.0019 | 0.133 | **3** | 0.0057 | 0.138 | **Zn(BF_4_)_2_** | 0.0015 | 0.189 | 0.540 |
| E3 | **A** | 0.0019 | 0.133 | **1** | 0.0057 | 0.122 | **Zn(NTf_2_)_2_** | 0.0015 | 0.105 | 0.640 |
| E4 | **A** | 0.0019 | 0.133 | **3** | 0.0057 | 0.138 | **Zn(BF_4_)_2_** | 0.0015 | 0.105 | 0.624 |
| E5 | **A** | 0.0029 | 0.200 | **1** | 0.0057 | 0.122 | **Zn(NTf_2_)_2_** | 0.0015 | 0.189 | 0.489 |
| E6 | **A** | 0.0029 | 0.200 | **3** | 0.0057 | 0.138 | **Zn(BF_4_)_2_** | 0.0015 | 0.189 | 0.473 |
| E7 | **A** | 0.0029 | 0.200 | **1** | 0.0057 | 0.122 | **Zn(NTf_2_)_2_** | 0.0015 | 0.105 | 0.573 |
| E8 | **A** | 0.0029 | 0.200 | **3** | 0.0057 | 0.138 | **Zn(BF_4_)_2_** | 0.0015 | 0.105 | 0.557 |
| F1 | **A** | 0.0019 | 0.133 | **1** | 0.0057 | 0.122 | **Zn(NTf_2_)_2_** | 0.0019 | 0.237 | 0.508 |
| F2 | **A** | 0.0019 | 0.133 | **3** | 0.0057 | 0.138 | **Zn(BF_4_)_2_** | 0.0019 | 0.237 | 0.492 |
| F3 | **A** | 0.0019 | 0.133 | **1** | 0.0057 | 0.122 | **Zn(NTf_2_)_2_** | 0.0019 | 0.131 | 0.614 |
| F4 | **A** | 0.0019 | 0.133 | **3** | 0.0057 | 0.138 | **Zn(BF_4_)_2_** | 0.0019 | 0.131 | 0.598 |
| F5 | **A** | 0.0029 | 0.200 | **1** | 0.0057 | 0.122 | **Zn(NTf_2_)_2_** | 0.0019 | 0.237 | 0.441 |
| F6 | **A** | 0.0029 | 0.200 | **3** | 0.0057 | 0.138 | **Zn(BF_4_)_2_** | 0.0019 | 0.237 | 0.425 |
| F6 | **A** | 0.0029 | 0.200 | **1** | 0.0057 | 0.122 | **Zn(NTf_2_)_2_** | 0.0019 | 0.131 | 0.547 |
| F8 | **A** | 0.0029 | 0.200 | **3** | 0.0057 | 0.138 | **Zn(BF_4_)_2_** | 0.0019 | 0.131 | 0.531 |

**S4.2 Automated Characterisation**

### **S4.2.1 Automated Data Analysis**

This study was characterised using ^1^H NMR with the automated analysis of the raw data carried out using the Python script moc_nmr_analyser_refined.py, adapted from moc_nmr_analyser.py. The script was only adapted to alter the imine peak chemical shift region to 8.4-8.8 ppm at the same 10000 a.u. threshold and to change the categorization of the aldehyde conversion. The percentage intensity relative to the largest imine peak was calculated and categorized into three outcomes – (i) aldehyde is below 5% and categorized as minor, (ii) aldehyde between 5-20% and categorized as residual, and (iii) aldehyde is above 20% and categorized as significant. All ^1^H NMR automated analysis scripts can be found on the GitHub page (https://github.com/GreenawayLab/development-automated-workflow mocs/tree/master/nmr).

**S4.3 Characterisation Data**

Following the automated analysis of the raw ^1^H NMR data, manual inspection was undertaken. For each of the 48 precursor combinations, a spectrum has been included, grouped by ligand (**A1** or **A3**) and metal salt ratio. All raw data can be found on Zenodo at https://doi.org/10.5281/zenodo.14183035.

**
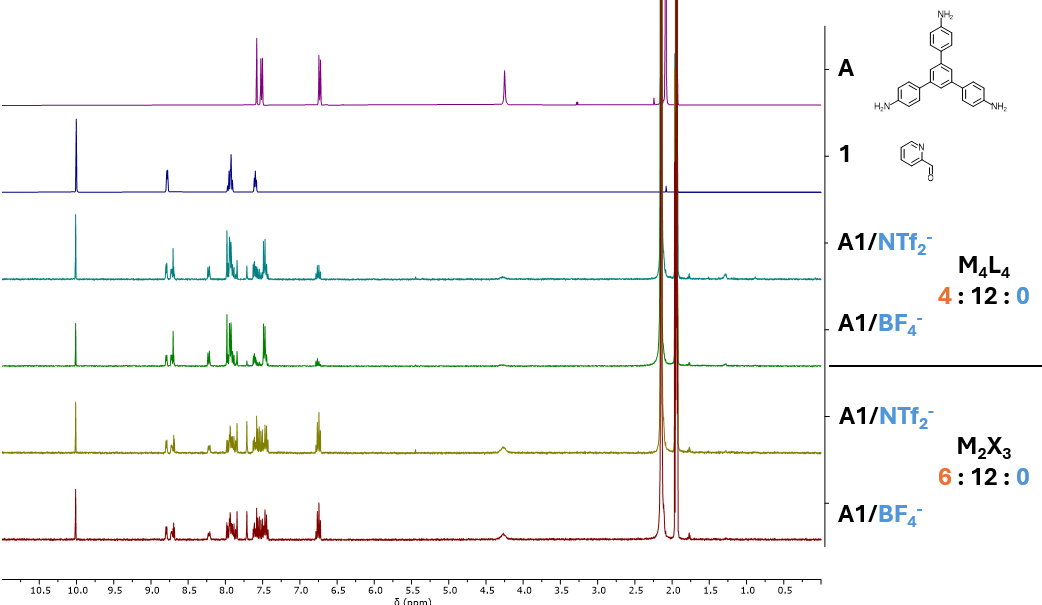
**

**Figure S79:** ^1^H NMR (CD_3_CN) spectra from the top down: **A**, **1**, **A1/NTf_2_^-^** 4:12:0, **A1/BF_4_^-^** 4:12:0, **A1/NTf_2_^-^** 6:12:0, and **A1/BF_4_^-^** 6:12:0, where ratios are of triamine:aldehyde:metal salt.

**
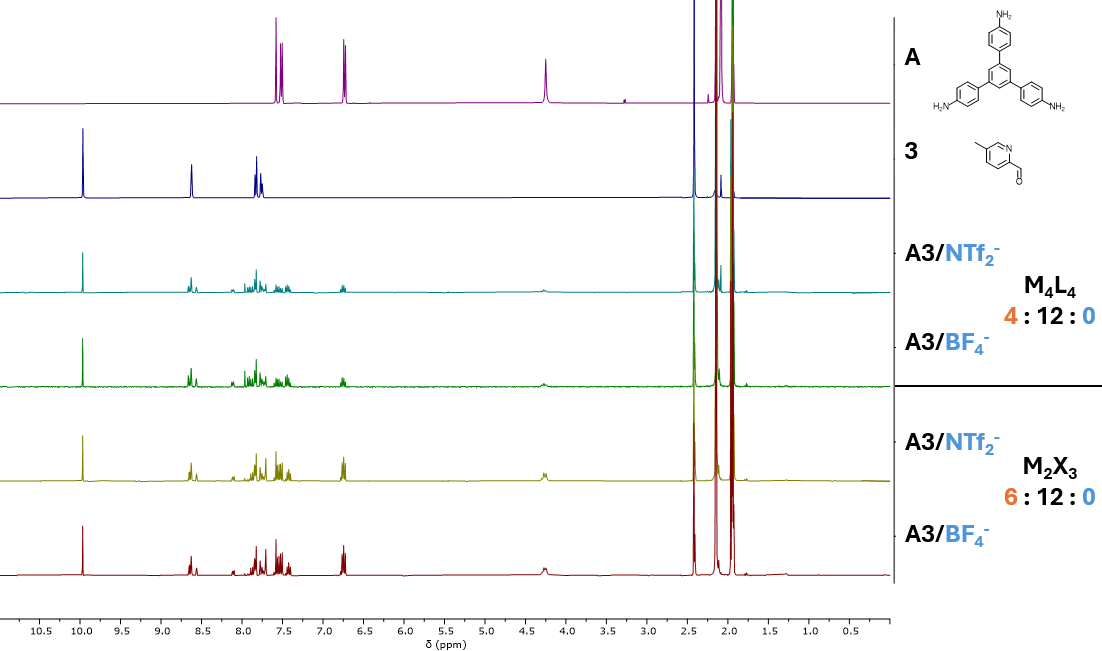
**

**Figure S80:** ^1^H NMR (CD_3_CN) spectra from the top down: **A**, **3**, **A3/NTf_2_^-^** 4:12:0, **A3/BF_4_^-^** 4:12:0, **A3/NTf_2_^-^** 6:12:0, and **A3/BF_4_^-^** 6:12:0, where ratios are of triamine:aldehyde:metal salt.


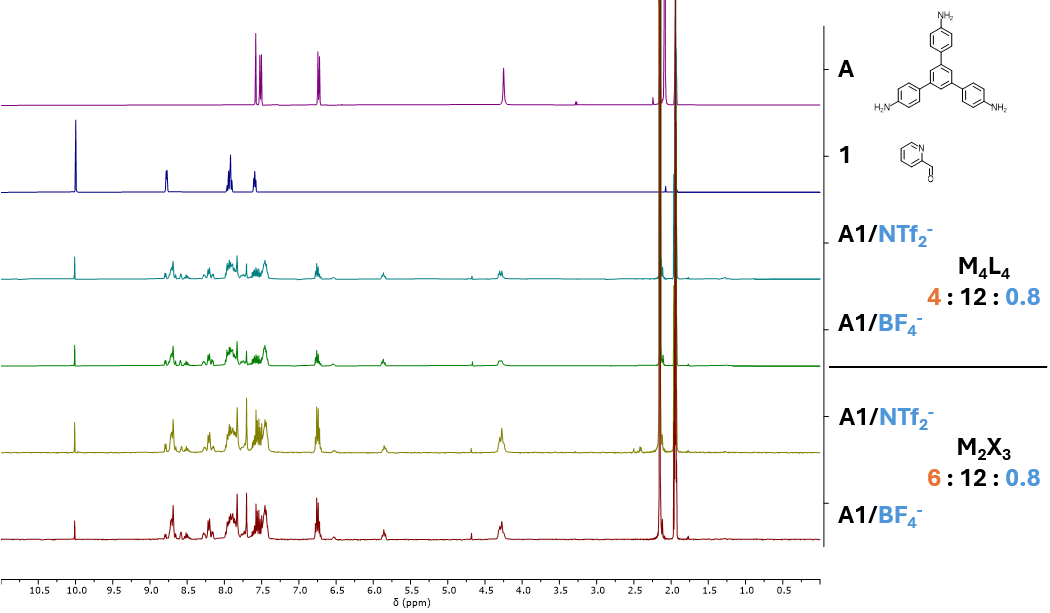


**Figure S81:** ^1^H NMR (CD_3_CN) spectra from the top down: **A**, **1**, **A1/NTf_2_^-^** 4:12:0.8, **A1/BF_4_^-^** 4:12:0.8, **A1/NTf_2_^-^** 6:12:0.8, and **A1/BF_4_^-^** 6:12:0.8, where ratios are of triamine:aldehyde:metal salt.

**
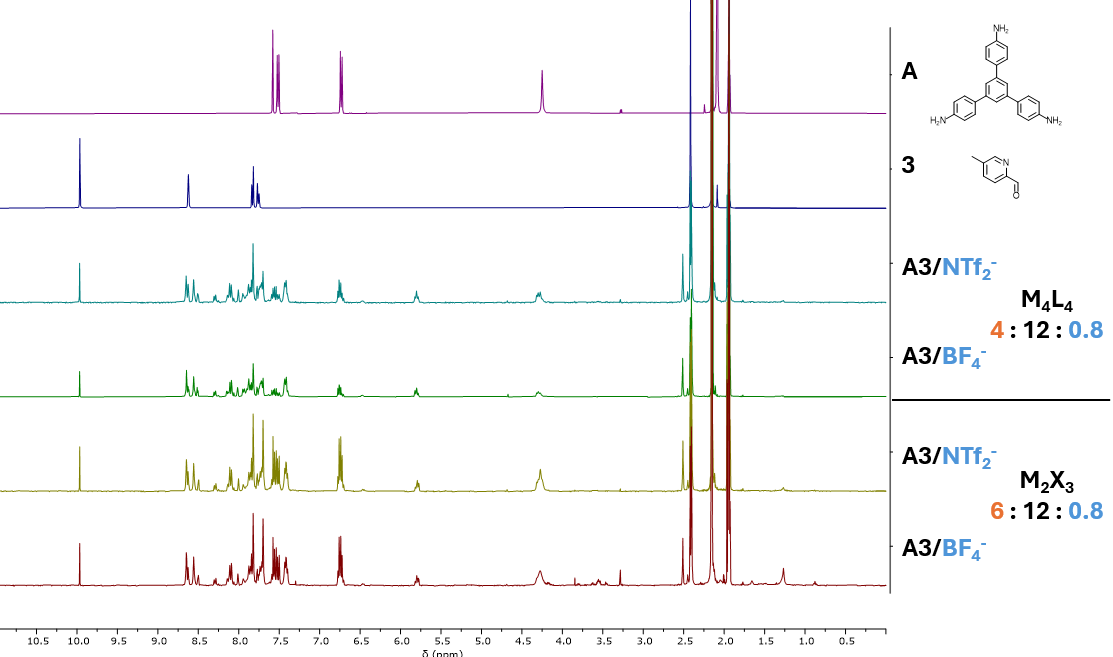
**

**Figure S82:** ^1^H NMR (CD_3_CN) spectra from the top down: **A**, **3**, **A3/NTf_2_^-^** 4:12:0.8, **A3/BF_4_^-^** 4:12:0.8, **A3/NTf_2_^-^** 6:12:0.8, and **A3/BF_4_^-^** 6:12:0.8, where ratios are of triamine:aldehyde:metal salt.

**
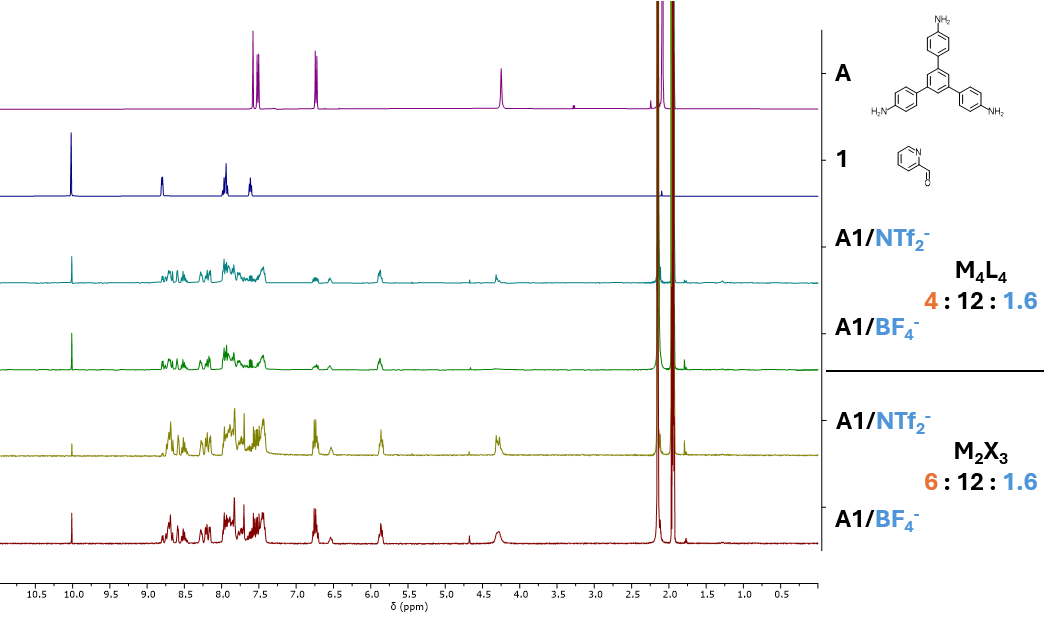
**

**Figure S83:** ^1^H NMR (CD_3_CN) spectra from the top down: **A**, **1**, **A1/NTf_2_^-^** 4:12:1.6, **A1/BF_4_^-^** 4:12:1.6, **A1/NTf_2_^-^** 6:12:1.6, and **A1/BF_4_^-^** 6:12:1.6, where ratios are of triamine:aldehyde:metal salt.

**
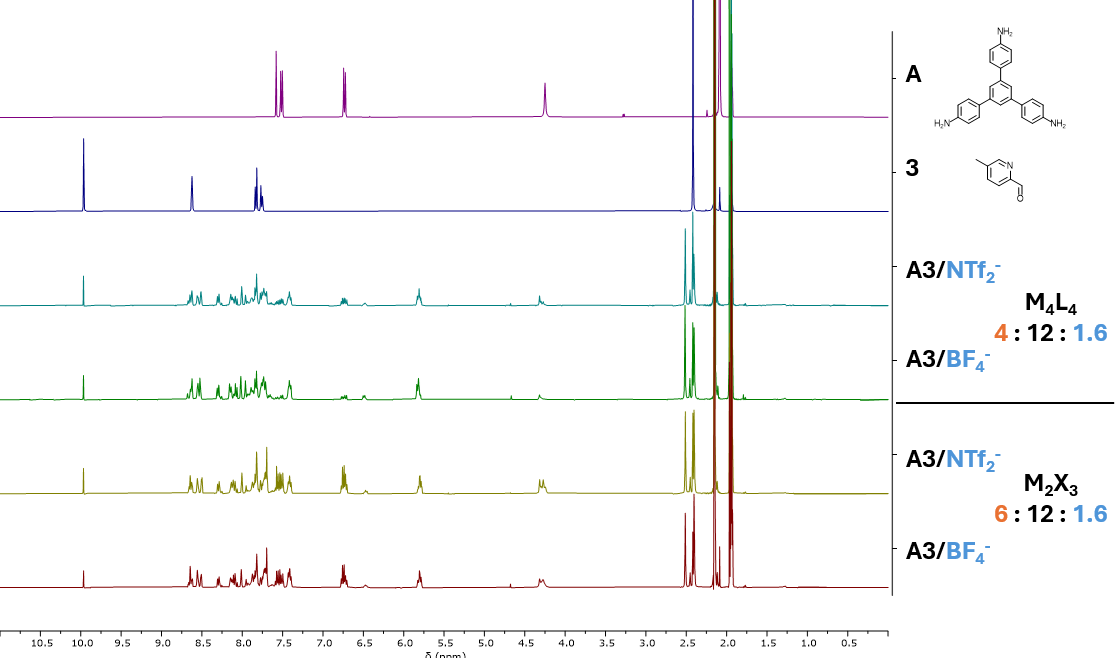
**

**Figure S84:** ^1^H NMR (CD_3_CN) spectra from the top down: **A**, **3**, **A3/NTf_2_^-^** 4:12:1.6, **A3/BF_4_^-^** 4:12:1.6,  **A3/NTf_2_^-^** 6:12:1.6, and **A3/BF_4_^-^** 6:12:1.6, where ratios are of triamine:aldehyde:metal salt.

**
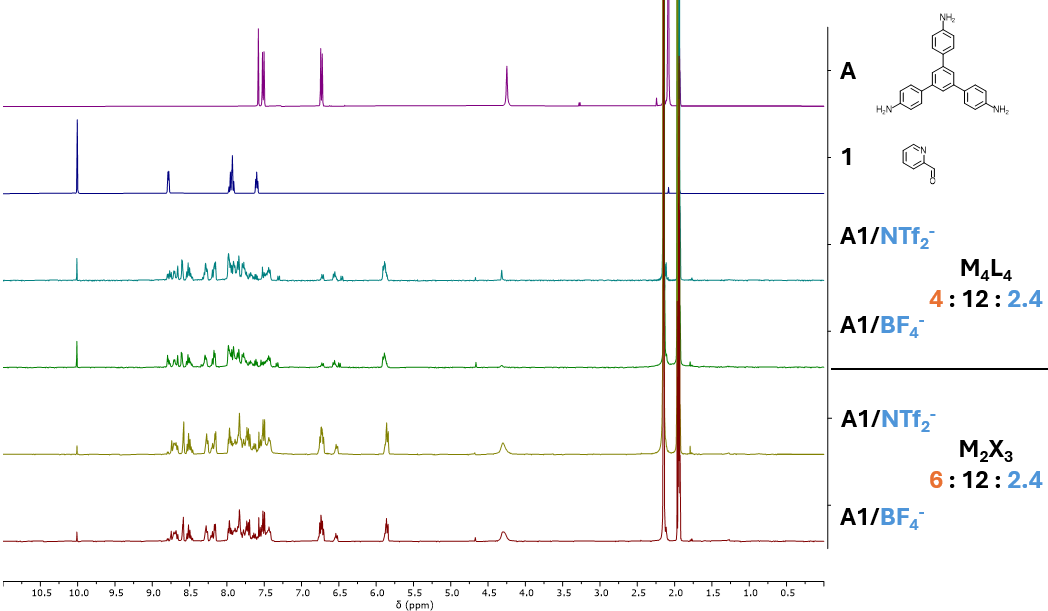
**

**Figure S85:** ^1^H NMR (CD_3_CN) spectra from the top down: **A**, **1**, **A1/NTf_2_^-^** 4:12:2.4, **A1/BF_4_^-^** 4:12:2.4, **A1/NTf_2_^-^** 6:12:2.4, and **A1/BF_4_^-^** 6:12:2.4, where ratios are of triamine:aldehyde:metal salt.

**
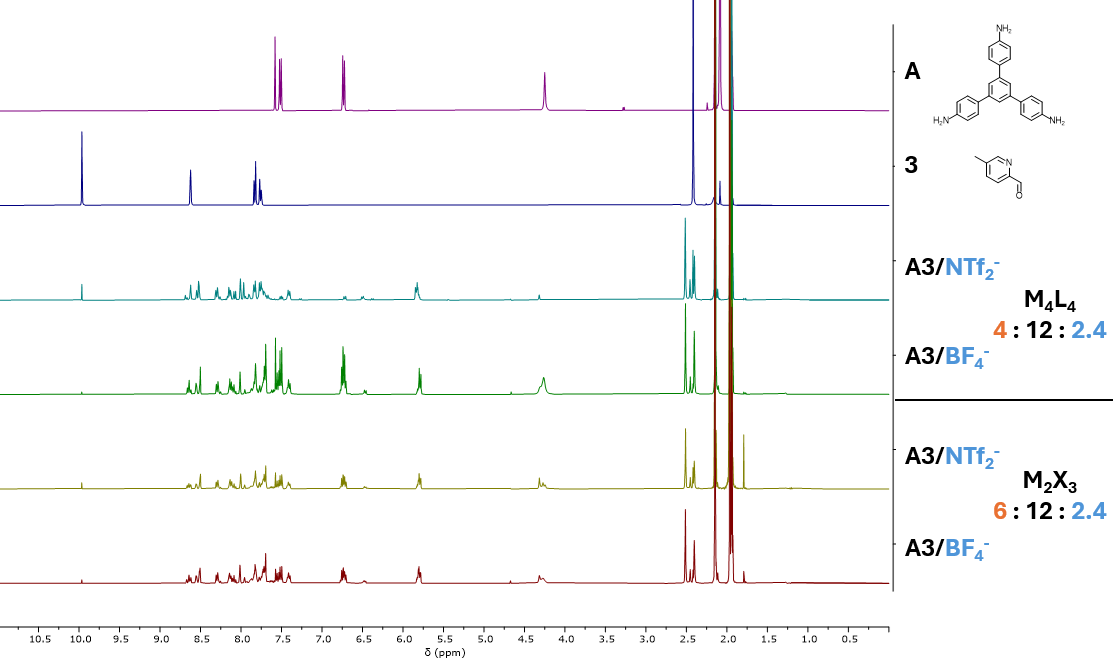
**

**Figure S86:** ^1^H NMR (CD_3_CN) spectra from the top down: **A**, **3**, **A3/NTf_2_^-^** 4:12:2.4, **A3/BF_4_^-^** 4:12:2.4, **A3/NTf_2_^-^** 6:12:2.4, and **A3/BF_4_^-^** 6:12:2.4, where ratios are of triamine:aldehyde:metal salt.


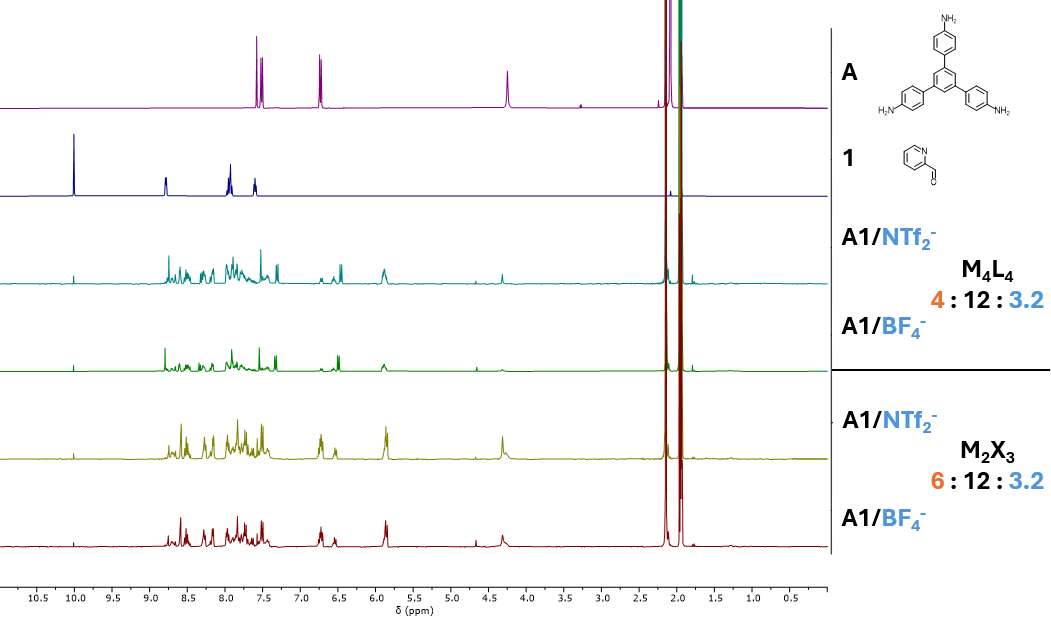


**Figure S87:** ^1^H NMR (CD_3_CN) spectra from the top down: **A**, **1**, **A1/NTf_2_^-^** 4:12:3.2, **A1/BF_4_^-^** 4:12:3.2, **A1/NTf_2_^-^** 6:12:3.2, and **A1/BF_4_^-^** 6:12:3.2, where ratios are of triamine:aldehyde:metal salt.

**
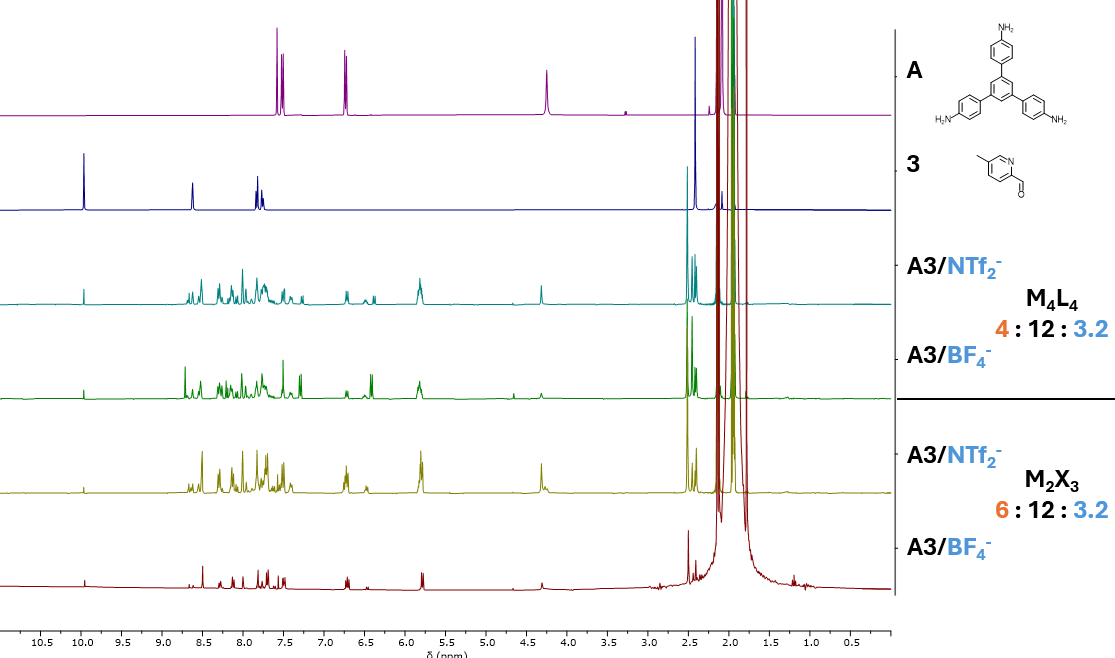
**

**Figure S88:** ^1^H NMR (CD_3_CN) spectra from the top down: **A**, **3**, **A3/NTf_2_^-^** 4:12:3.2, **A3/BF_4_^-^** 4:12:3.2, **A3/NTf_2_^-^** 6:12:3.2, and **A3/BF_4_^-^** 6:12:3.2, where ratios are of triamine:aldehyde:metal salt.

**
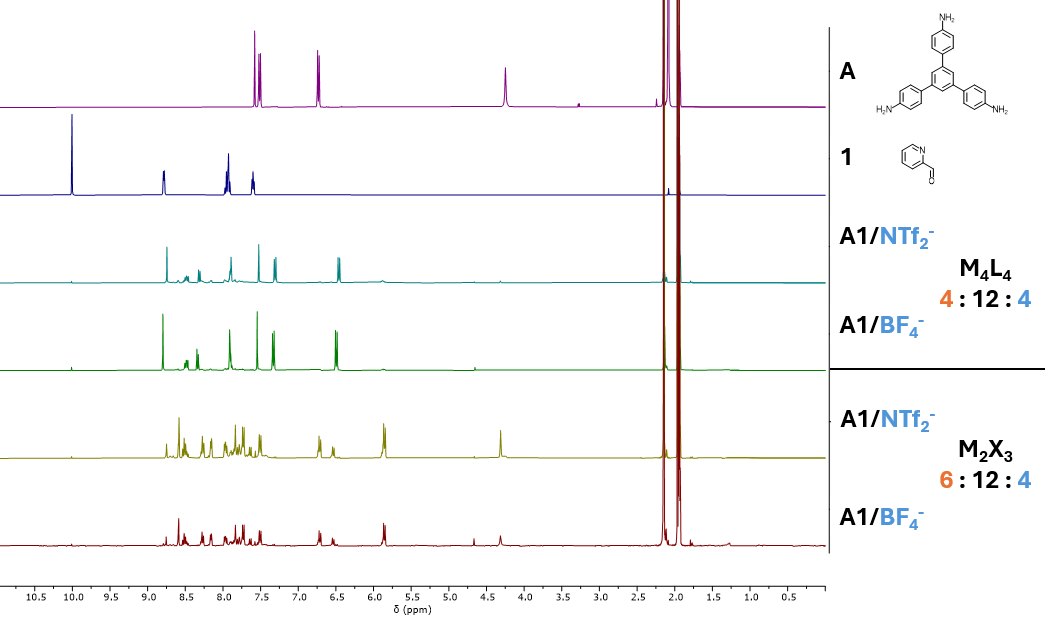
**

**Figure S89:** ^1^H NMR (CD_3_CN) spectra from the top down: **A**, **1**, **A1/NTf_2_^-^** 4:12:4, **A1/BF_4_^-^** 4:12:4, **A1/NTf_2_^-^** 6:12:4, and **A1/BF_4_^-^** 6:12:4, where ratios are of triamine:aldehyde:metal salt.

**
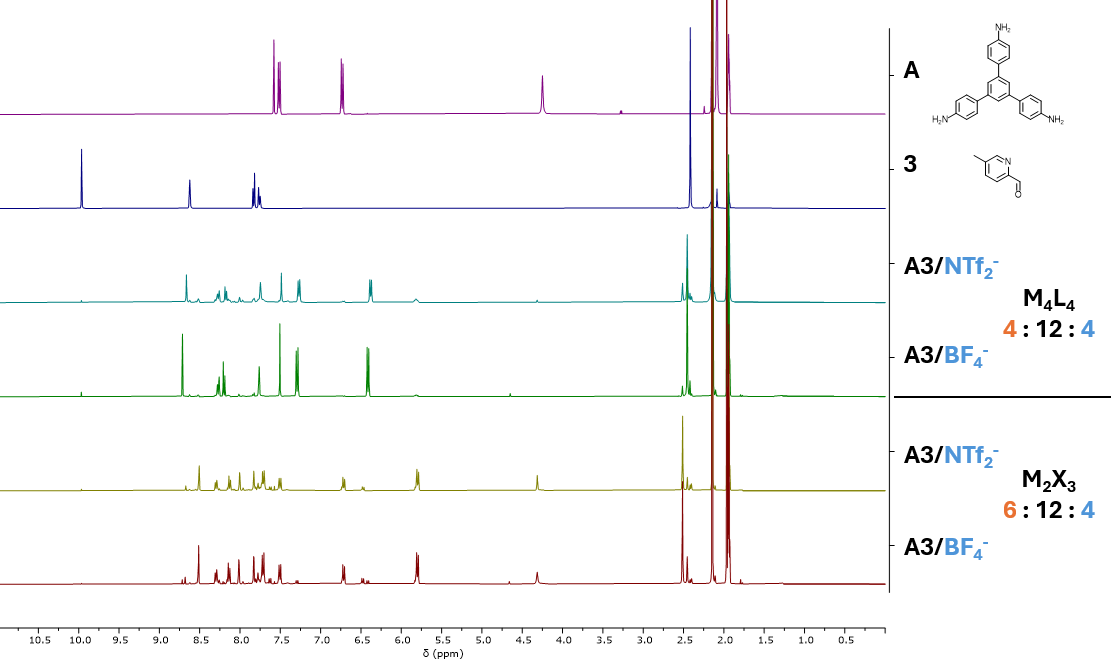
**

**Figure S90:** ^1^H NMR (CD_3_CN) spectra from the top down: **A**, **3**, **A3/NTf_2_^-^** 4:12:4, **A3/BF_4_^-^** 4:12:4, **A3/NTf_2_^-^** 6:12:4, and **A3/BF_4_^-^** 6:12:4, where ratios are of triamine:aldehyde:metal salt.


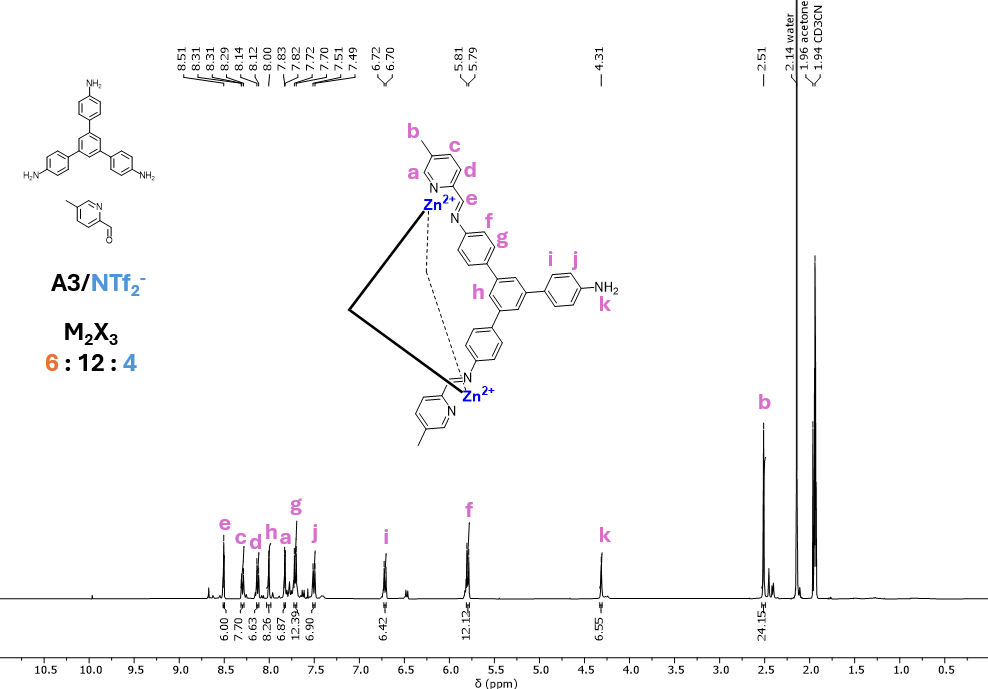


**Figure S91:** ^1^H NMR (CD_3_CN) spectra of precursor combination **A3/NTf_2_^-^** 6:12:4 where ratios are of triamine:aldehyde:metal salt.


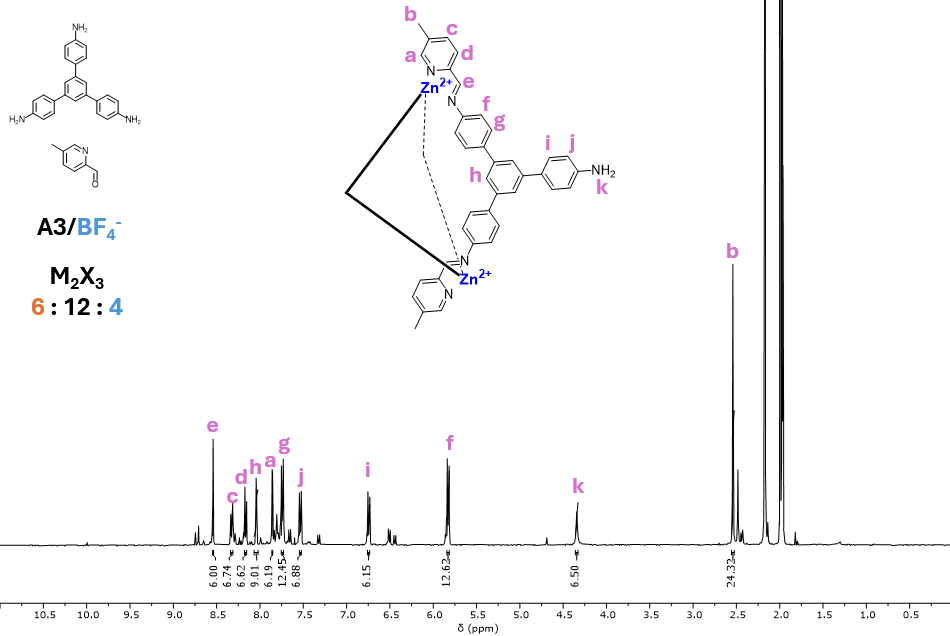


**Figure S92:** ^1^H NMR (CD_3_CN) spectra of precursor combination **A3/BF_4_^-^** 6:12:4 where ratios are of triamine:aldehyde:metal salt.


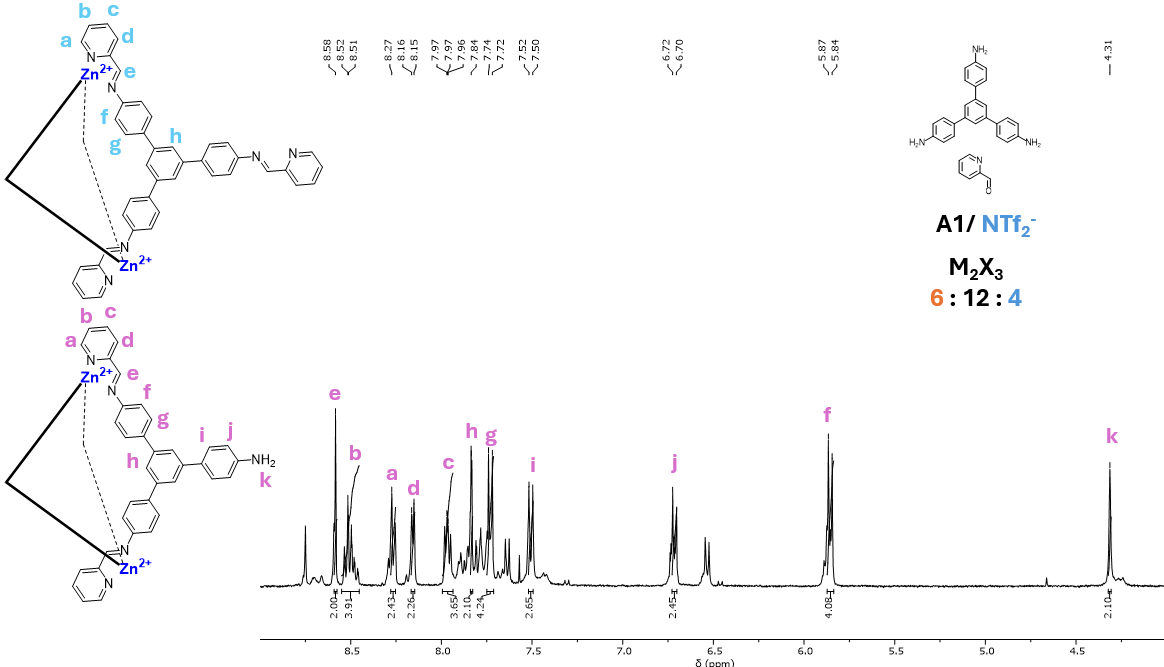


**Figure S93:** ^1^H NMR (CD_3_CN) spectra of precursor combination **A3/NTf_2_^-^** 6:12:4 where ratios are of triamine:aldehyde:metal salt. A complex mixture of **M_2_X_3_** structures with the presence of the ditopic intermediate (I) into the structures. Spectral complexity may be attributed to a complex mixture of helicate structures which may form: **M_2_I_3_**,  **M_2_I_2_L**, **M_2_IL_2_** and **M_2_L_3_**.


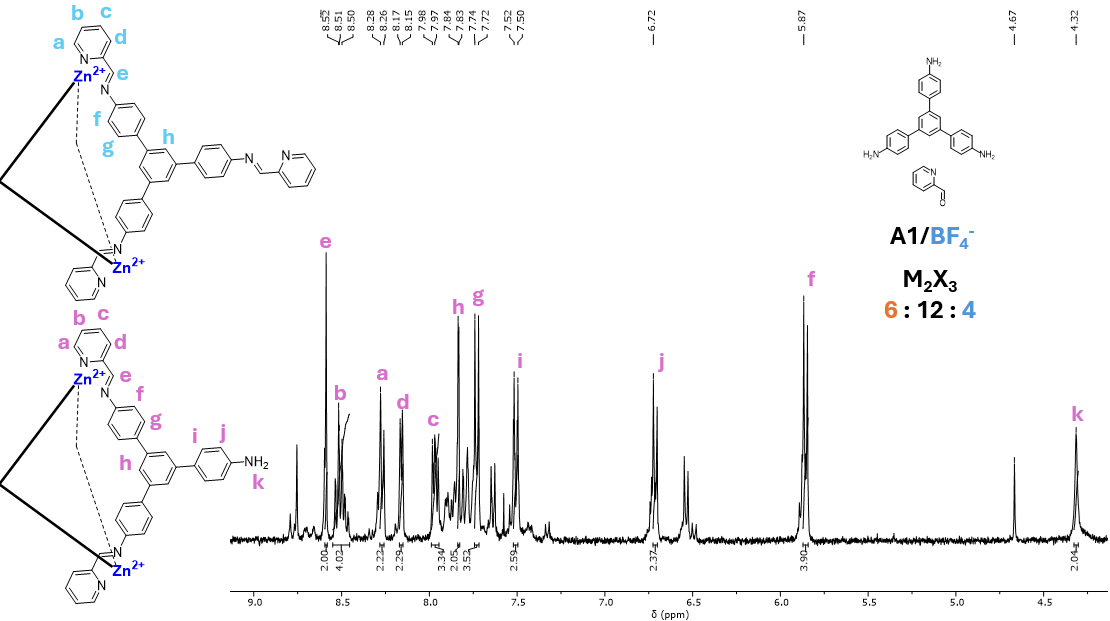


**Figure S94:** ^1^H NMR (CD_3_CN) spectra of precursor combination **A3/BF_4_^-^** 6:12:4 where ratios are of triamine:aldehyde:metal salt. A complex mixture of **M_2_X_3_** structures with the presence of the ditopic intermediate (I) into the structures. Spectral complexity may be attributed to a complex mixture of helicate structures which may form: **M_2_I_3_**,  **M_2_I_2_L**, **M_2_IL_2_** and **M_2_L_3_**.

# **S5. Computational Modelling**

In the manuscript, we described the automated screening of the self-assembly of two triamines with four aldehydes, yielding eight different triamine ligands, with three different Zn(II) counter ions (NTf_2_^-^), (OTf^-^) and (BF_4_^-^) into possible metal-organic cage topologies with the formulae **M_N_L_N_** or **M_2_X_3_** where X may be the tri-topic ligand (L) or the di-topic intermediate (I) formed from a di-substituted imine condensation product between the triamine and aldehyde precursors. The modelling of the possible topologies was conducted on helicates (**M_2_X_3_**), tetrahedrons (**M_4_L_4_**) and icosahedrons (**M_12_L_12_**). Here, the Supramolecular Tool Kit (*stk*) and high-performance computing (HPC) are used to automate the *in-silico* construction of these structures.^9^ Semi-empirical quantum mechanical calculations were carried out using the *stko*^10^ and OPTIM^11,12^ interfaces to the *xtb* program.^13^ Additionally, single-point energy calculations at the r_2_SCAN-3c^17^ level using ORCA.^14^ Molecular Modelling of geometry optimization steps that could not be automated are performed via MM3^15^ modelling through the Scigress^16^ program. No general approach leading to converged structures could be created for the **M_2_X_3_** structures and these are therefore left out of the discussion. Cavity analysis was performed on the tetrahedrons using the Python library *pyWindow*

All computational modelling code and models are available at https://github.com/GreenawayLab/development-automated-workflow-mocs/tree/master/MOC_modelling.

### **S5.1 Tetrahedrons, M_4_L_4_**

The *in-silico* construction of eight different tetrahedrons was automated using *stk* and *stko.* Two different core linkers were used and four different imine-moieties, giving eight different ligands (**A1-4** and **B1-4**). Only one diastereomer was constructed for each choice of linker and imine-moiety. First, the facial-Λ octahedral complex was built with the correct imine-moiety and optimized using the MCHammer algorithm as implemented in *stk*. Next a linker-building block was created from the corresponding SMILES strings. The tetrahedrons were constructed from the complex and linker building-blocks using the *stk.cage.M4L4Tetrahedron* function. The optimization sequence that followed was analogous to that previously published:^17^

1. After *stk* construction, a geometry optimization was performed using the *stk.Collapser* algorithm. A step size of 0.1 Å was used and the algorithm was stopped when the distance between the building blocks reached below the threshold of 1.5 Å.
2. Geometry optimizations were performed with UFF4MOF in the General Utility Lattice Program (GULP) through the interface in the *stko* program. This optimization was performed in two steps:
   1. With the conjugate gradient algorithm (“conj unit”)
   2. With the second-order Newton Raphson algorithm and BFGS hessian update.
3. A conformer search was performed from the resulting structure using high-temperature molecular dynamics (MD) in the NVT ensemble. One MD run was performed using UFF4MOF and GULP at 400 K, using the leapfrog Verlet integrator. A short equilibration was run for 0.1 ps and the production run is performed for 2.0 ps with a time step of 0.5 fs. From the production run, 10 conformers were extracted at 0.2 ps intervals.
4. The lowest energy cage conformer obtained in the former step was optimized using GFN2-xTB^18,19^ with the ‘*normal*’ convergence criterion and no implicit solvent.
5. A single-point energy evaluation at the r^2^SCAN-3c^20^ level is performed on the GFN2-xTB optimized structure using ORCA.^14^ A tolerance setting of *TightSCF* was applied and the RI-J approximation was included in the calculation using the *def2/J* keyword.

The Python code used for this can be found in *ConstructionTet.py*. All eight tetrahedrons were successfully optimized and their geometry optimized structures can be found in the manuscript Figure 6 and the corresponding GFN2-xTB total energies can be found in Table 2. The tetrahedrons created using the methyl-substituted imine-motifs are isomers of each other and therefore their total energies can be compared with each other.

For scaled-up **cage 1, cage 2** and **cage 3**, their corresponding tetrahedron models were taken for cavity analysis using the Python library *pyWindow*, which has previously been reported to accurately predict and reproduce supramolecular assemblies pore sizes.^21^ pyWindow encapsulates the modelled cage in a sphere of points with vectors pointing into the centre of mass of the cage. The pore cavity and windows are determined as regions of space where the vectors were not intercepted by atoms before reaching the centre of mass. Pore volume as an extension was calculated as the maximum sphere size on those vector points that did not have any overlap with the van der Waals radii of the atoms of the cage. The number of windows and the average window diameter was computationally predicted to indicate how a guest molecule may diffuse into the cage and to indicate its structural stability. As **cage 1** and **cage 2** differ only by their corresponding counterions which were not modelled computationally, the same tetrahedron and calculated diameter and volume were used. The script for pore analysis can be found on GitHub (https://github.com/GreenawayLab/development-automated-workflow-mocs/tree/master/MOC_modelling).

**Table S11:** Pore analysis of the scaled-up **cages 1-3** using *pyWindow*. The same modelled tetrahedron was used for **cage 1** and **cage 2**.

| Cage | Diameter (Å) | Volume (Å^3^) |
| --- | --- | --- |
| **Cage 1** | 5.11 | 69.69 |
| **Cage 2** | 5.11 | 69.69 |
| **Cage 3** | 5.07 | 68.32 |

### **S5.2 Icosahedrons, M_12_L_12_**

The *in-silico* construction of the MOCs with icosahedral architectectures was not automated. These structures are composed of several non-symmetric octahedral complexes with meridional stereochemistry, which makes their construction using *stk* increasingly challenging. The structures were modelled using the MM3^15^ force filed in Scigress.^16^ The four structures constructed from the larger ligands (**A1-4**) were used as input structures for consecutive geometry optimizations at the GFN-FF^22^ and GFN2-xTB level using the OPTIM interface to the *xtb* program.^11,12^ The OPTIM program was selected as no convergence could be reached through the ORCA program. The GFN-FF optimized geometries can be found in Figure S78. Geometry optimizations at the GFN2-xTB level again through OPTIM, using the GFN-FF result as input, did not lead to any converged structures.

An icosahedral MOC structure was constructed from the smaller ligands for **B1-4** using MM3 modelling in Scigress. The MM3 result shows a distorted geometry, from which it can already be concluded that the icosahedron is not a suitable topology for the **B1-4** linkers. A geometry optimization at the GFN-FF level on the MM3 result of the **B1** linker, followed by a geometry optimization at the GFN2-xTB level, showed that a converged structure could only be obtained by allowing detachment of several imine-nitrogens from the Zn(II) metal. Isomers of this icosahedron with the three other imine moieties were not modelled as a similar result can reasonably be expected. Based on these results, the smaller linker is not expected to self-assemble into an icosahedral topology with any of the imine moieties studied in this work.


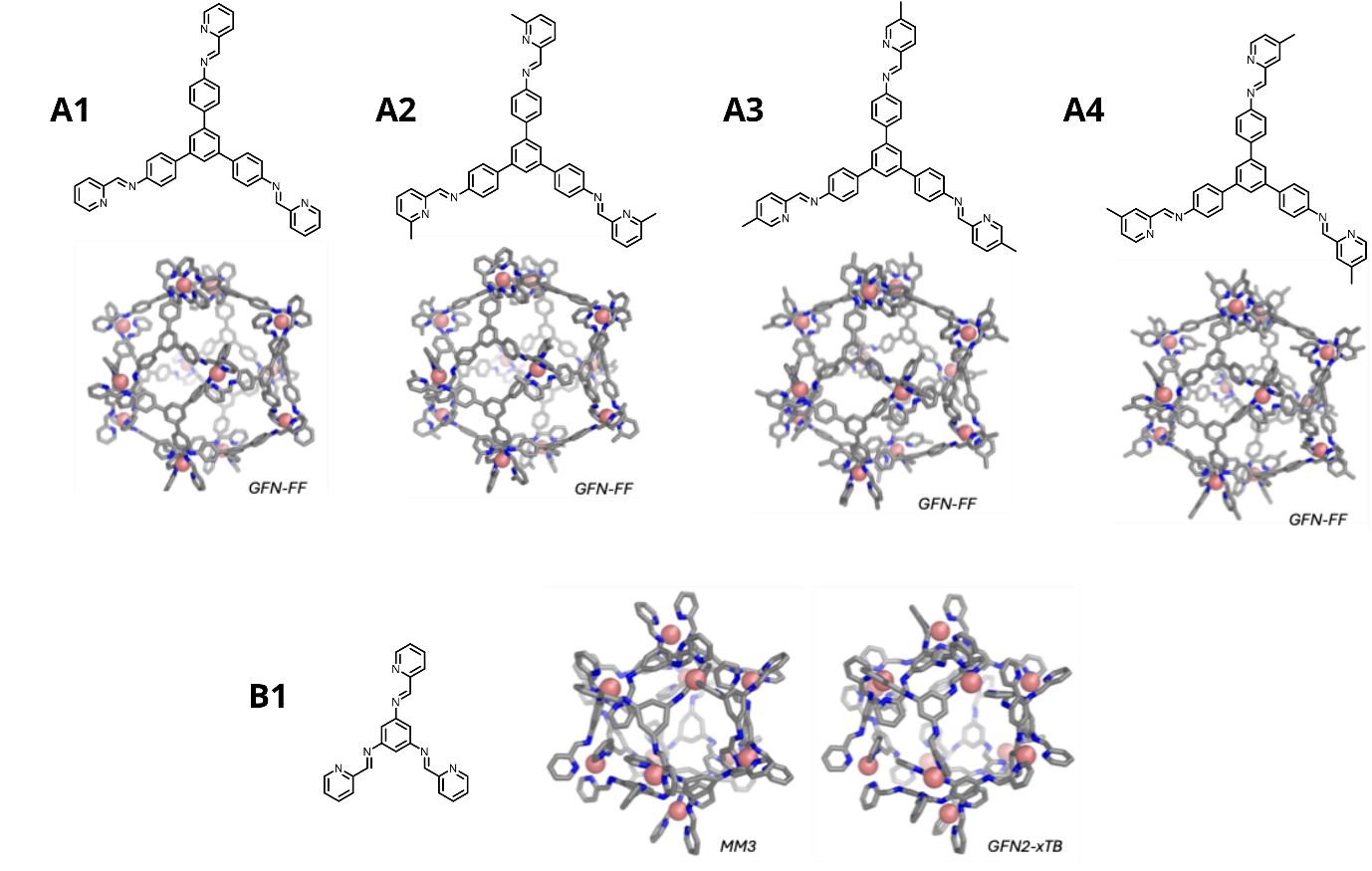


**Figure S95:** (Top) Geometries of four icosahedral MOCs (**A1-4**), constructed using MM3 modelling in Scigress and optimized at the GFN-FF level using the *xtb* interface of the OPTIM program. (Bottom) MM3 and GFN2-xTB optimized geometries of the icosahedral MOC **B1**. Hydrogen atoms are omitted for clarity. Meridional Zn atoms are shown in salmon/pink, nitrogen atoms in blue and carbon atoms in grey.

**Software versions:**

- Python: 3.11.9
- GULP^23,24^: 5.1
- xTB^13^: 6.6.1
- stk^25^: 2024.3.28.0
- stko^10^: 2023.11.13.0
- rdkit^26^: 2024.3.3
- OPTIM^11,12^:

# **S6. Automated Guest Assay**

**Automated host-guest binding general method:** A list of neutral guests that have previously been used in MOC host-guest binding studies was collected, totalling 32 guests. Host-guest binding studies were performed on the Opentrons OT-2 (robot v7.2.1 and 7.2.1 app version) with the same settings as the automated synthesis screen. Stock solutions of MOC **cage 1** were made up at 0.0008 mmol/mL in acetonitrile-*d_3_* and each of the guests 0.0214 mmol/mL in acetonitrile-*d_3_*. Stock solutions of host MOCs and guests were placed in 24-well 8 mL plates and 6-well 25 mL plates. For each host-guest binding combination 0.5 mL (0.0004 mmol) of MOC **cage 1** and 0.075 mL (0.0016 mmol) of guest were combined totalling 0.575 mL and a host-guest ratio of 1:4 equivalence. Host-guest binding combinations were conducted in a VWR 96 well 2 mL deep well collection plate and, once stock solutions were combined, a square capmat was placed on top before leaving the plate to stir overnight on a Heidolph Titramax 1000 package plate shaker.^27,28^ Following this the solutions were transferred to NMR tubes by the OT-2 in a 3D printed 96-well NMR tube holder plate and ^1^H NMR analysis was undertaken. A subset of guests was selected (toluene, n-octane, carbon tetrachloride, o-xylene, t-butanol) to compare binding between **cage** **1**, **cage 2** and **cage 3**. The code required to replicate the protocol is available at https://github.com/GreenawayLab/development-automated-workflow-mocs.git. In cases where encapsulation was inferred to take place, a new set of resonances (host-guest) were observed at a different chemical shift to the original host cage shifts, along with the set of signals corresponding to free cage (host). For cases where a set of host and host-guest chemical resonances were observed, slow exchange interactions were inferred. It was assumed that only one guest can bind. Therefore, the relative integrations of the resonances corresponding to the free and bound hosts, along with the known initial concentrations of host and guest, allowed an estimate of the association constant (*K*_a_) for each host-guest combination. All the ^1^H NMR raw data after 1 and 7 days can be found on Zenodo at https://doi.org/10.5281/zenodo.14183035.

**Figure S96:** The 32 guests used in the automated guest assay to screen for host-guest binding.

**Table S12:** Stock solutions of MOC **cage 1**, **cage 2**, **cage 3** and guests 1-32.

| **Reagent** | **Molecular Weight /**  **g mol^-1^** | **Stock Solution concentration / mg mL^-1^** | **Stock Solution concentration / mmol mL^-1^** | **Mass for stock solution (mg)** | **Precursor Density (g/mL)** | **Volume for stock if liquid (μL)** |
| --- | --- | --- | --- | --- | --- | --- |
| **cage 1** | 4968.07 | 4.00 | 0.0008 | 80 | - | - |
| **cage 2** | 3424.75 | 2.77 | 0.0008 | 14 | - | - |
| **cage 3** | 5136.26 | 4.15 | 0.0008 | 21 | - | - |
| **cyclohexane** | 86.11 | 1.80 | 0.0214 | 14.4 | 0.779 | 18.50 |
| **cyclohexene** | 82.08 | 1.76 | 0.0214 | 14.1 | 0.811 | 17.34 |
| **cyclopentane** | 70.08 | 1.50 | 0.0214 | 12.0 | 0.751 | 15.98 |
| **cyclopentene** | 68.06 | 1.46 | 0.0214 | 11.7 | 0.771 | 15.12 |
| **cyclohexanone** | 98.07 | 2.10 | 0.0214 | 16.8 | 0.948 | 17.72 |
| **cyclohexanol** | 100.09 | 2.14 | 0.0214 | 17.1 | 0.962 | 17.82 |
| **cyclooctane** | 112.13 | 2.40 | 0.0214 | 19.2 | 0.8334 | 23.05 |
| **cyclopentanol** | 86.07 | 1.84 | 0.0214 | 14.7 | 0.949 | 15.54 |
| **benzene** | 78.05 | 1.67 | 0.0214 | 13.4 | 0.876 | 15.27 |
| **pyridine** | 79.04 | 1.69 | 0.0214 | 13.5 | 0.982 | 13.79 |
| **isoxazole** | 69.02 | 1.48 | 0.0214 | 11.8 | 1.08 | 10.95 |
| **toluene** | 92.06 | 1.97 | 0.0214 | 15.8 | 0.867 | 18.19 |
| **n-pentane** | 72.09 | 1.54 | 0.0214 | 12.4 | 0.626 | 19.73 |
| **n-hexane** | 86.11 | 1.84 | 0.0214 | 14.8 | 0.661 | 22.32 |
| **n-octane** | 114.14 | 2.44 | 0.0214 | 19.6 | 0.703 | 27.82 |
| **carbon tetrachloride** | 151.88 | 3.29 | 0.0214 | 26.3 | 1.59 | 16.56 |
| **chloroform** | 49.99 | 2.55 | 0.0214 | 20.4 | 1.49 | 13.72 |
| **dichloromethane** | 83.95 | 1.82 | 0.0214 | 14.5 | 1.33 | 10.93 |
| **1,3,5-trifluorobenzene** | 132.02 | 2.83 | 0.0214 | 22.6 | 1.277 | 17.71 |
| **o-xylene** | 106.08 | 2.27 | 0.0214 | 18.2 | 0.86 | 21.13 |
| **m-xylene** | 106.08 | 2.27 | 0.0214 | 18.2 | 0.86 | 21.13 |
| **p-xylene** | 106.08 | 2.27 | 0.0214 | 18.2 | 0.86 | 21.13 |
| **adamantane** | 136.13 | 2.92 | 0.0214 | 23.3 | - | - |
| **1-bromoadamantane** | 214.04 | 4.60 | 0.0214 | 36.8 | - | - |
| **mesitylene** | 120.09 | 2.57 | 0.0214 | 20.6 | 0.864 | 23.82 |
| **t-butanol** | 74.07 | 1.59 | 0.0214 | 12.7 | 0.781 | 16.25 |
| **s-butanol** | 74.07 | 1.59 | 0.0214 | 12.7 | 0.806 | 15.74 |
| **n-butanol** | 74.07 | 1.59 | 0.0214 | 12.7 | 0.81 | 15.67 |
| **1,3,5-trimethoxybenzene** | 168.08 | 3.60 | 0.0214 | 28.8 | - | - |
| **THF** | 72.06 | 1.54 | 0.0214 | 12.3 | 0.888 | 13.90 |
| **1,4-dioxane** | 88.05 | 1.89 | 0.0214 | 15.1 | 1.03 | 14.65 |
| **1,3-dioxane** | 88.05 | 1.89 | 0.0214 | 15.1 | 1.03 | 14.65 |

Calibration 1 resulted in dispense accuracies within a 10% threshold and high enough to complete the automated MOC synthesis screen of precursors. However, for the automated host-guest binding screen a higher accuracy was required. Therefore, calibration 2 was conducted with targeted volumes of 75 μL (for guest addition) and 250 μL (2 x 250 μL = 500 μL MOC addition). Calibration 1 highlighted a small under dispense of ~10 μL, so volumes of 85 μL and 260 μL were also included in calibration 2. A third middle volume of 150 μL (and therefore 160 μL) was also selected to see the accuracy. The lower volumes of 75 and 85 μL both dispensed under the target volume with a difference of 4 μL, whereas the 150 μL dispensed under by 10 μL. The higher volumes matched the results from the lower volumes of -5 μL. As an overall result, volumes of 85 and 260 μL were written in the script to ensure dispenses of 75 and 250 μL were definitely achieved, with the extra volume within a 10% error for acetonitrile using the OT-2. The accuracies of dispenses to achieve the 1:4 host:guest equivalence were further exemplified in the ^1^H NMR spectra below.

**Table S13:** Calibration 2 of Opentrons OT-2 with acetonitrile across volumes 75, 85, 150, 160, 250 and 260 μL for accurate dispenses for the guest assay. Dispenses were carried out three times per targeted volume and averaged. Gantry speeds were set to 250 mm s^-1^ for the X, Y and Z axes. The 300 μL pipette aspiration and dispense flowrate was set to 70 μL s^-1^ and transfers included a pre-saturation step (100 μL aspiration, 10 s delay, 100 μL dispense back into the source vial) to prevent dripping and allow accurate transfers. Vials were pre-weighed before the dispense and after, with the actual dispensed volume calculated using the density of acetonitrile and the accuracy against the target volume calculated.

| **Target Dispense Volume / μL** | **Average Dispensed Volume / μL** | **Δvol / μL** | **Standard deviation** |
| --- | --- | --- | --- |
| 75 | 71 | -4 | 1.69 |
| 85 | 81 | -4 | 1.57 |
| 150 | 140 | -10 | 2.79 |
| 160 | 155 | -5 | 1.35 |
| 250 | 245 | -5 | 2.58 |
| 260 | 256 | -4 | 2.48 |


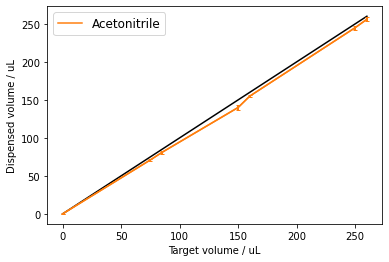


**Figure S97:** Calibration 2 of the Opentrons OT-2 with acetonitrile (orange) compared to the targeted dispense volumes (black). Dispenses were measured and averaged with error bars included showing the standard deviation.

**Table S14:** Stock solution and solvent volumes used in host-guest binding study on the Opentrons OT-2 platform for the automated MOC property screening. The ratio of host:guest was 1:4, 0.0004:0.0016 totalling 0.002 mmol. The volumes have been corrected to 0.520 mL (2 x 260 μL dispenses using the OT-2) of host and 0.085 mL (85 μL) of guest following Calibration 2.

| **Vial** | **Host** | **Amount of host (mmol)** | **Volume of host stock solution (mL)** | **Guest** | **Amount of guest (mmol)** | **Volume of guest stock solution (mL)** |
| --- | --- | --- | --- | --- | --- | --- |
| A1 | **cage 1** | 0.0004 | 0.520 | cyclohexane | 0.0016 | 0.085 |
| A2 | **cage 1** | 0.0004 | 0.520 | cyclohexene | 0.0016 | 0.085 |
| A3 | **cage 1** | 0.0004 | 0.520 | cyclopentane | 0.0016 | 0.085 |
| A4 | **cage 1** | 0.0004 | 0.520 | cyclopentene | 0.0016 | 0.085 |
| B1 | **cage 1** | 0.0004 | 0.520 | cyclohexanone | 0.0016 | 0.085 |
| B2 | **cage 1** | 0.0004 | 0.520 | cyclohexanol | 0.0016 | 0.085 |
| B3 | **cage 1** | 0.0004 | 0.520 | cyclooctane | 0.0016 | 0.085 |
| B4 | **cage 1** | 0.0004 | 0.520 | cyclopentanol | 0.0016 | 0.085 |
| C1 | **cage 1** | 0.0004 | 0.520 | benzene | 0.0016 | 0.085 |
| C2 | **cage 1** | 0.0004 | 0.520 | pyridine | 0.0016 | 0.085 |
| C3 | **cage 1** | 0.0004 | 0.520 | isoxazole | 0.0016 | 0.085 |
| C4 | **cage 1** | 0.0004 | 0.520 | toluene | 0.0016 | 0.085 |
| D1 | **cage 1** | 0.0004 | 0.520 | n-pentane | 0.0016 | 0.085 |
| D2 | **cage 1** | 0.0004 | 0.520 | n-hexane | 0.0016 | 0.085 |
| D3 | **cage 1** | 0.0004 | 0.520 | n-octane | 0.0016 | 0.085 |
| D4 | **cage 1** | 0.0004 | 0.520 | carbon tetrachloride | 0.0016 | 0.085 |
| E1 | **cage 1** | 0.0004 | 0.520 | chloroform | 0.0016 | 0.085 |
| E2 | **cage 1** | 0.0004 | 0.520 | dichloromethane | 0.0016 | 0.085 |
| E3 | **cage 1** | 0.0004 | 0.520 | 1,3,5-trifluorobenzene | 0.0016 | 0.085 |
| E4 | **cage 1** | 0.0004 | 0.520 | o-xylene | 0.0016 | 0.085 |
| F1 | **cage 1** | 0.0004 | 0.520 | m-xylene | 0.0016 | 0.085 |
| F2 | **cage 1** | 0.0004 | 0.520 | p-xylene | 0.0016 | 0.085 |
| F3 | **cage 1** | 0.0004 | 0.520 | adamantane | 0.0016 | 0.085 |
| F4 | **cage 1** | 0.0004 | 0.520 | 1-bromoadamantane | 0.0016 | 0.085 |
| G1 | **cage 1** | 0.0004 | 0.520 | mesitylene | 0.0016 | 0.085 |
| G2 | **cage 1** | 0.0004 | 0.520 | t-butanol | 0.0016 | 0.085 |
| G3 | **cage 1** | 0.0004 | 0.520 | s-butanol | 0.0016 | 0.085 |
| G4 | **cage 1** | 0.0004 | 0.520 | n-butanol | 0.0016 | 0.085 |
| H1 | **cage 1** | 0.0004 | 0.520 | 1,3,5-trimethoxybenzene | 0.0016 | 0.085 |
| H2 | **cage 1** | 0.0004 | 0.520 | THF | 0.0016 | 0.085 |
| H3 | **cage 1** | 0.0004 | 0.520 | 1,4-dioxane | 0.0016 | 0.085 |
| H4 | **cage 1** | 0.0004 | 0.520 | 1,3-dioxane | 0.0016 | 0.085 |


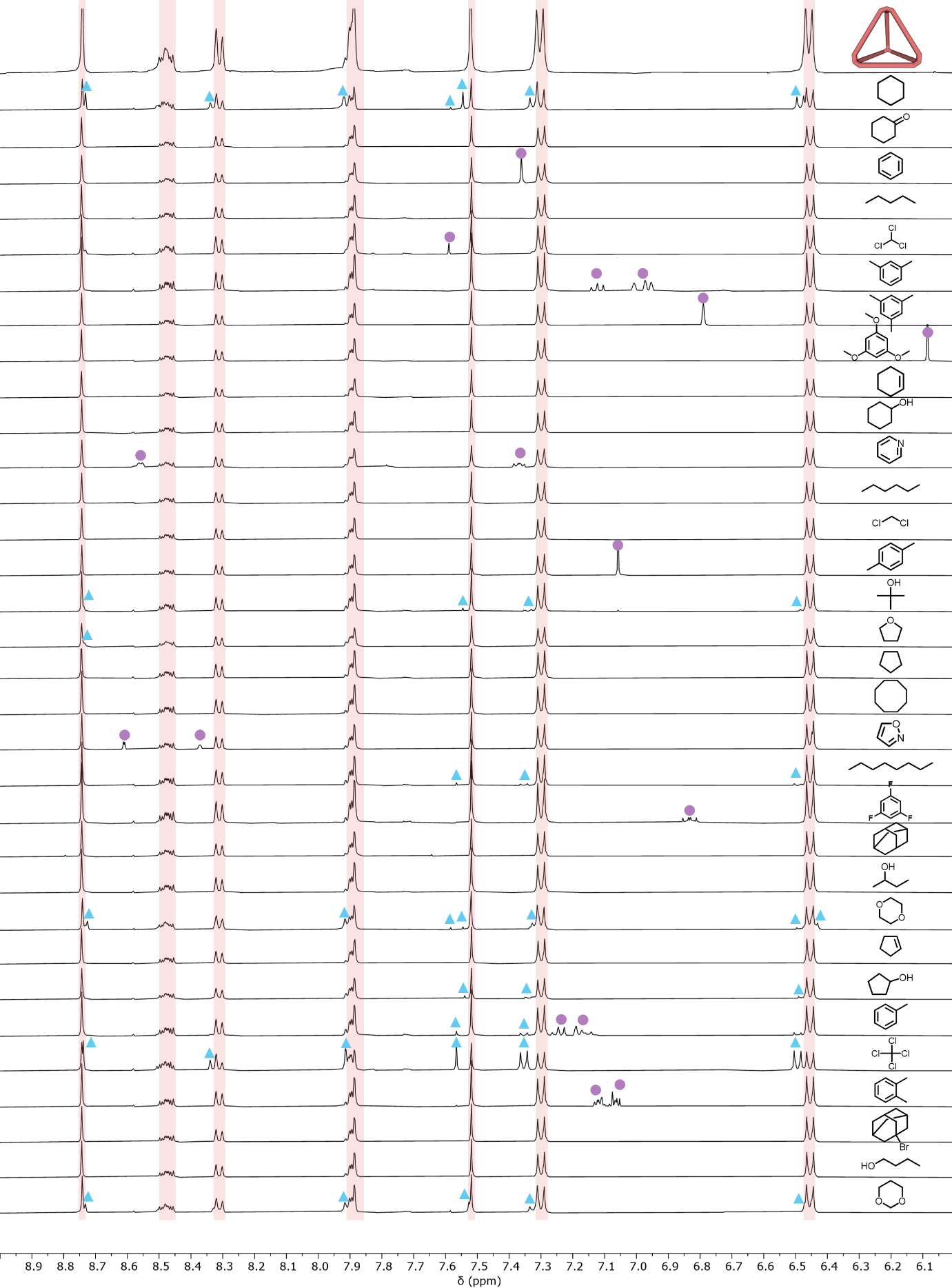


**Figure S98:** Stacked ^1^H NMR spectra of the host MOC **cage 2** (top) and the 32 host+guest binding mixtures at a 1:4 host:guest. Host peaks are highlighted in a red box and guest peaks as purple circles. Host-guest complex peaks are shown on the spectrum as blue triangles. Host-guest binding studies are shown after 1 day.


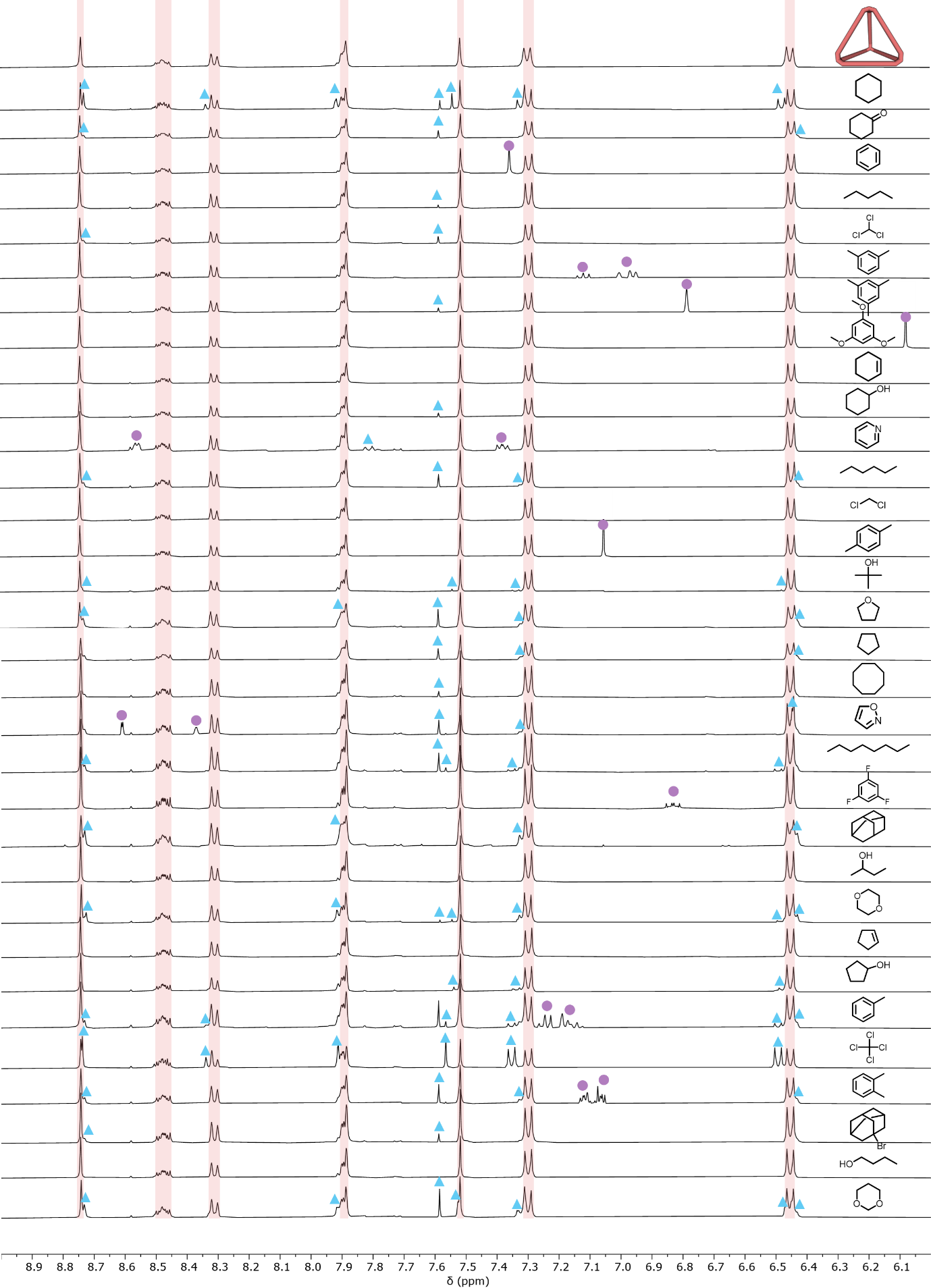
**Figure S99:** Stacked ^1^H NMR spectra of the host MOC **cage 2** (top) and the 32 host+guest binding mixtures at a 1:4 host:guest. Host peaks are highlighted in a red box and guest peaks as purple circles. Host-guest complex peaks are shown on the spectrum as blue triangles. Host-guest binding studies are shown after 7 days.


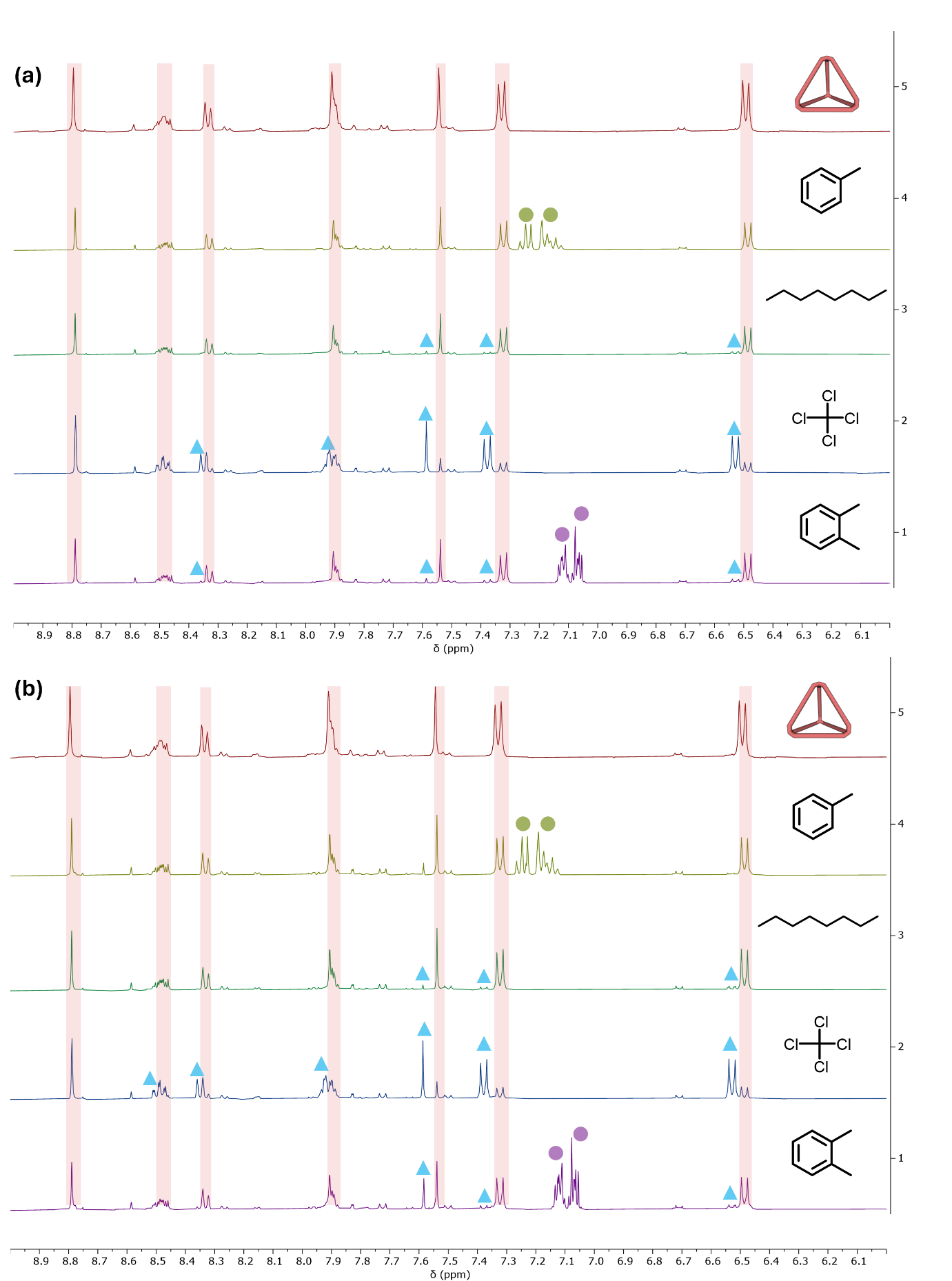
**Figure S100:** Stacked ^1^H NMR spectra of the host MOC **cage 2** (top) and the four host+guest binding mixtures at a 1:4 host:guest ratio with toluene, n-octane, carbon tetrachloride and o-xylene (top-bottom). Host peaks are highlighted in a red box and guest peaks as circles to match the colour of the spectrum. Host-guest complex peaks are shown on the spectrum as blue triangles. Host-guest binding studies are shown after 1 day (a) and after 7 days (b).

**Figure S101:** Stacked ^1^H NMR spectra of the host MOC **cage 3** (top) and the four host+guest binding mixtures at a 1:4 host:guest ratio with toluene, n-octane, carbon tetrachloride and o-xylene (top-bottom). Host peaks are highlighted in a red box and guest peaks as circles to match the colour of the spectrum. Host-guest complex peaks are shown on the spectrum as blue triangles. Host-guest binding studies are shown after 1 day (a) and after 7 days (b)
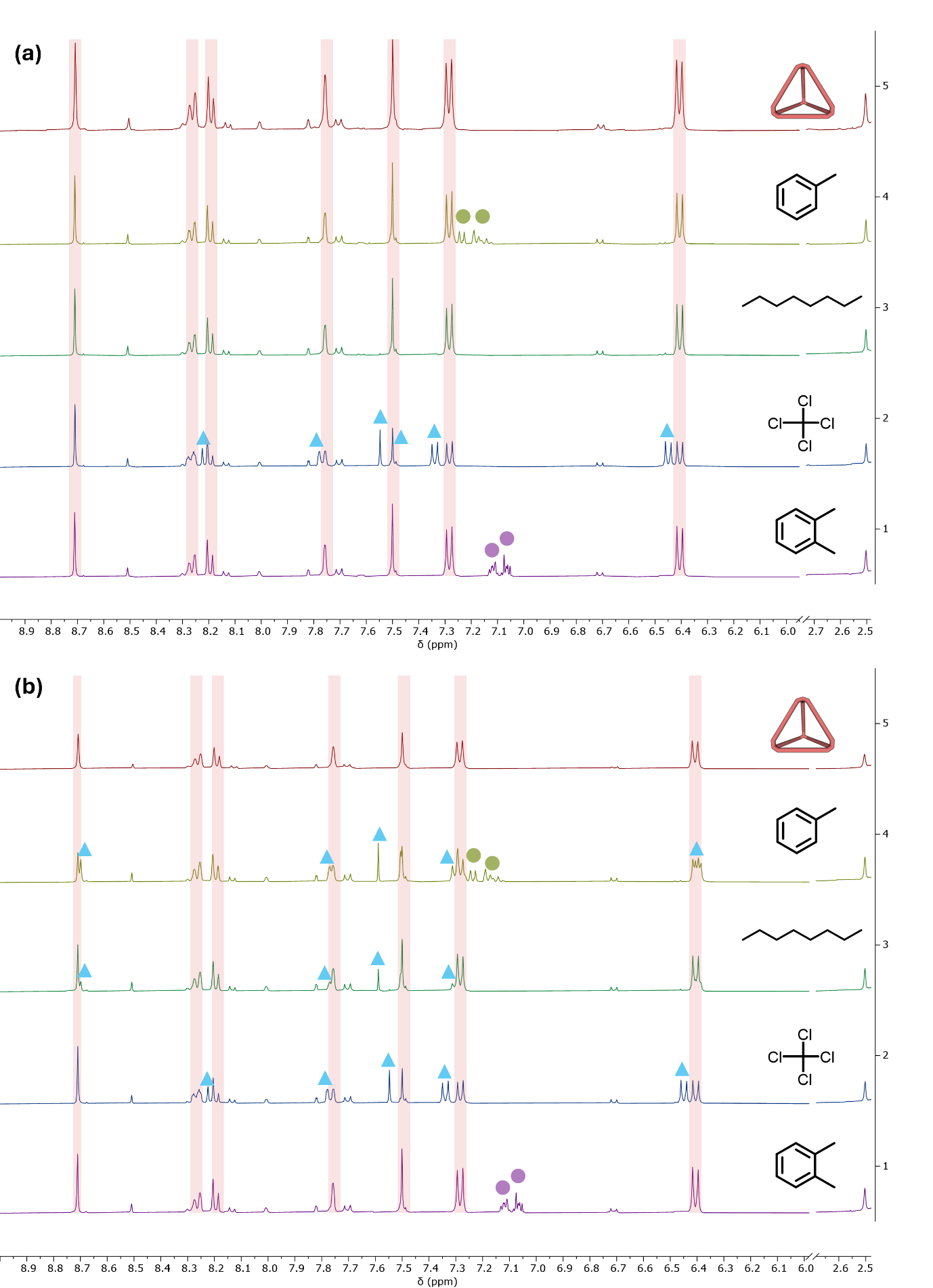
.

**Table S15:** Association constants (Ka) for the host-guest binding study between **cage 1** and the 32 guests. In cases where a previous study in the literature has been conducted, encapsulation is reported, however this was done in competitive binding studies with cyclohexane. In cases where ‘low’ is shown, encapsulation was too low to displace cyclohexane.^4,29^

| **Host** | **Guest** | ***K_a_***  **(This work)** | **Study in the Literature?** | ***K_a_***  **(Literature)** |
| --- | --- | --- | --- | --- |
| **cage 1** | cyclohexane | 2 × 10^2^ | Yes | 4.9 × 10^2^ |
| **cage 1** | cyclohexene | nonbinding | No | - |
| **cage 1** | cyclopentane | 1 × 10^2^ | Yes | 6.7 × 10^2^ |
| **cage 1** | cyclopentene | nonbinding | No | - |
| **cage 1** | cyclohexanone | 9 × 10^1^ | No | - |
| **cage 1** | cyclohexanol | 4 × 10^1^ | No | - |
| **cage 1** | cyclooctane | 3 × 10^1^ | Yes | Low |
| **cage 1** | cyclopentanol | 7 × 10^1^ | Yes | Low |
| **cage 1** | benzene | nonbinding | Yes | Low |
| **cage 1** | pyridine | 8 × 10^1^ | No | - |
| **cage 1** | isoxazole | 8 × 10^1^ | No | - |
| **cage 1** | toluene | 1 × 10^2^ | No | - |
| **cage 1** | n-pentane | 3 × 10^1^ | Yes | Low |
| **cage 1** | n-hexane | 9 × 10^1^ | Yes | nonbinding |
| **cage 1** | n-octane | 9 × 10^1^ | No | - |
| **cage 1** | carbon tetrachloride | 5 × 10^2^ | Yes | 1.2 × 10^3^ |
| **cage 1** | chloroform | 7 × 10^1^ | Yes | 1.5 × 10^2^ |
| **cage 1** | dichloromethane | nonbinding | Yes | Low |
| **cage 1** | 1,3,5-trifluorobenzene | nonbinding | Yes | nonbinding |
| **cage 1** | o-xylene | 1 × 10^2^ | No | - |
| **cage 1** | m-xylene | nonbinding | No | - |
| **cage 1** | p-xylene | nonbinding | No | - |
| **cage 1** | adamantane | 1 × 10^2^ | Yes | 5.9 × 10^1^ |
| **cage 1** | 1-bromoadamantane | 4 × 10^1^ | Yes | nonbinding |
| **cage 1** | mesitylene | 4 × 10^1^ | No | - |
| **cage 1** | t-butanol | 2 × 10^1^ | Yes | Low |
| **cage 1** | s-butanol | nonbinding | No | - |
| **cage 1** | n-butanol | nonbinding | No | - |
| **cage 1** | 1,3,5-trimethoxybenzene | nonbinding | Yes | nonbinding |
| **cage 1** | THF | 1 × 10^2^ | No | - |
| **cage 1** | 1,4-dioxane | 2 × 10^1^ | No | - |
| **cage 1** | 1,3-dioxane | 2 × 10^1^ | No | - |


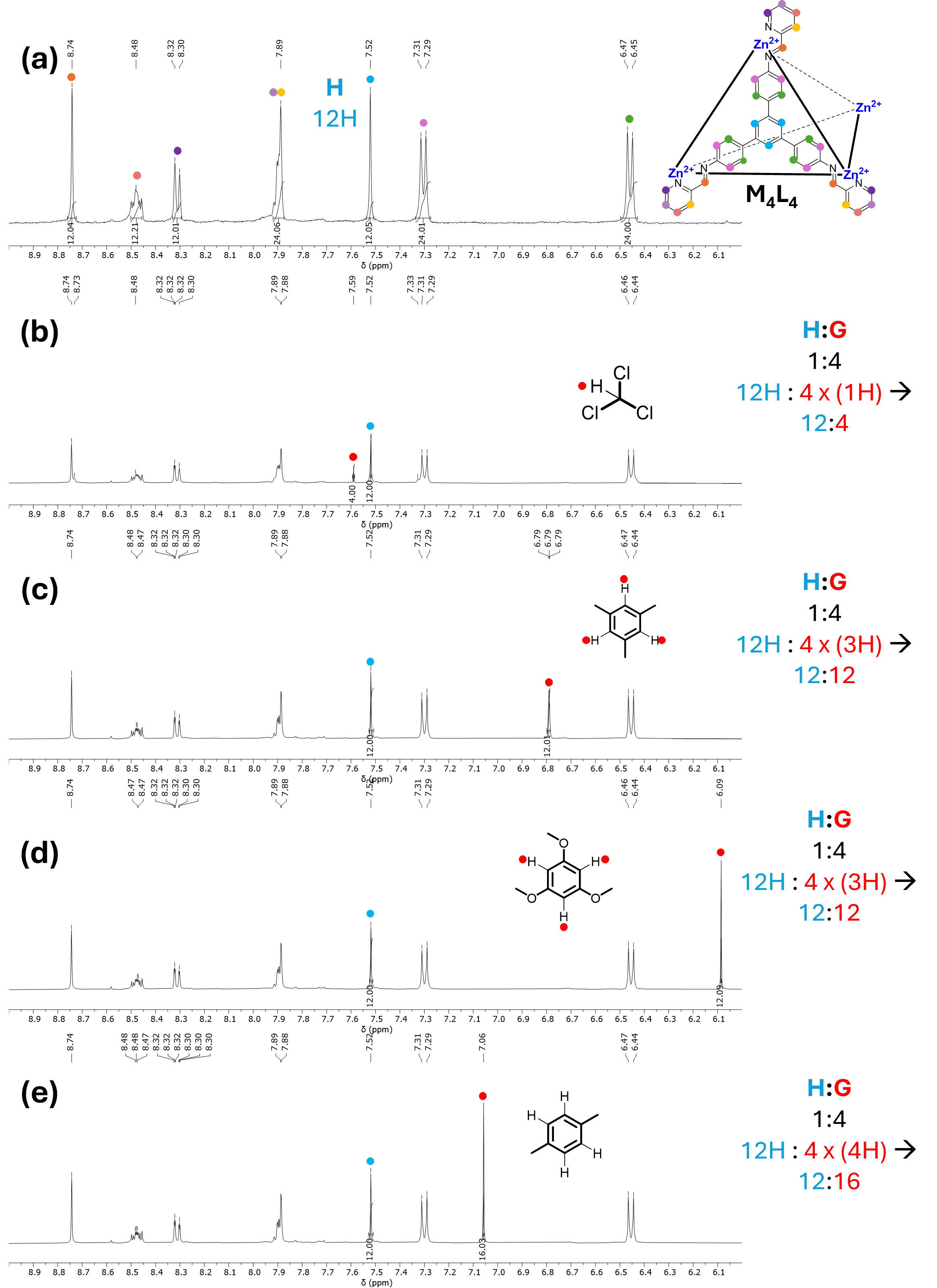


**Figure S102:** Stacked ^1^H NMR spectra of the host MOC **cage 1** (a) and four unbound guests after 1 day: chloroform (b) mesitylene (c) 1,3,5-trimethoxybenzene (d) and o-xylene (d). Confirmation of the 1:4 equivalence of host:guest was made through integrations of the host **cage 1** (blue circles) and of each unbound guest (red circles).

# **S7. References**

1 OT-2 Liquid Handler | Opentrons Lab Automation from $10,000 | Opentrons, https://opentrons.com/products/robots/ot-2/, (accessed 8 April 2024).

2 EquaVAP® 48-Well Evaporator (23048), https://www.analytical-sales.com/product/equavap-48-well-evaporator-23048/, (accessed 8 April 2024).

3 Sample Handling | Lab Automation, https://www.bruker.com/en/products-and-solutions/mr/nmr/nmr-automation/samplejet.html, (accessed 8 April 2024).

4 A. Jiménez, R. A. Bilbeisi, T. K. Ronson, S. Zarra, C. Woodhead and J. R. Nitschke, *Angew. Chem. Int. Ed.*, 2014, **53**, 4556–4560.

5 A. R. Basford, S. K. Bennett, M. Xiao, L. Turcani, J. Allen, K. E. Jelfs and R. L. Greenaway, *Chem. Sci.*, 2024, **15**, 6331–6348.

6 Sartorius&trade;&nbsp;MG 160 Grade White Quartz Microfiber Filters Without Binder -, https://www.fishersci.co.uk/shop/products/mg-160-grade-white-quartz-microfiber-filters-without-binder-7/11776055, (accessed 30 July 2024).

7 GreenawayLab/cagey GreenawayLab 2024.

8 pyOpenMS: A Python‐based interface to the OpenMS mass‐spectrometry algorithm library - Röst - 2014 - PROTEOMICS - Wiley Online Library, https://analyticalsciencejournals.onlinelibrary.wiley.com/doi/full/10.1002/pmic.201300246, (accessed 25 July 2023).

9 L. Turcani, A. Tarzia, F. T. Szczypiński and K. E. Jelfs, *J. Chem. Phys.*, 2021, **154**, 214102.

10 Bennett, S.; Tarzia, A.; Turcani, L. stko, 2022, https://github.com/JelfsMaterialsGroup/stko Computational Supramolecular Materials Discovery 2023.

11 P. A. Wesołowski, D. J. Wales and P. Pracht, *J. Phys. Chem. B*, 2024, **128**, 3145–3156.

12 OPTIM: A Program for Geometry Optimisation and Pathway Calculations http://www-wales.ch.cam.ac.uk/software.html.

13 C. Bannwarth, E. Caldeweyher, S. Ehlert, A. Hansen, P. Pracht, J. Seibert, S. Spicher and S. Grimme, *WIREs Comput. Mol. Sci.*, DOI:10.1002/wcms.1493.

14 F. Neese, F. Wennmohs, U. Becker and C. Riplinger, *J. Chem. Phys.*, 2020, **152**, 224108.

15 N. L. Allinger, Y. H. Yuh and J. H. Lii, *J. Am. Chem. Soc.*, 1989, **111**, 8551–8566.

16 SCIGRESS version FJ 2.6 (EU 3.1.9) build 5996.8255.20141202 (Fujitsu Limited, 2013)., https://www.fujitsu.com/global/solutions/business-technology/tc/sol/scigress/, (accessed 5 September 2023).

17 J. A. Davies, A. Tarzia, T. K. Ronson, F. Auras, K. E. Jelfs and J. R. Nitschke, *Angew. Chem. Int. Ed.*, 2023, **62**, e202217987.

18 C. Bannwarth, S. Ehlert and S. Grimme, *J. Chem. Theory Comput.*, 2019, **15**, 1652–1671.

19 M. Bursch, H. Neugebauer and S. Grimme, *Angew. Chem. Int. Ed.*, 2019, **58**, 11078–11087.

20 S. Grimme, A. Hansen, S. Ehlert and J.-M. Mewes, *J. Chem. Phys.*, 2021, **154**, 064103.

21 M. Miklitz and K. E. Jelfs, *J. Chem. Inf. Model.*, 2018, **58**, 2387–2391.

22 S. Spicher and S. Grimme, *Angew. Chem. Int. Ed.*, 2020, **59**, 15665–15673.

23 J. D. Gale, *J. Chem. Soc. Faraday Trans.*, 1997, **93**, 629–637.

24 J. D. Gale and A. L. Rohl, *Mol. Simul.*, 2003, **29**, 291–341.

25 L. Turcani, A. Tarzia, F. T. Szczypiński and K. E. Jelfs, *J. Chem. Phys.*, 2021, **154**, 214102.

26 RDKit: Open-source cheminformatics. https://www.rdkit.org, (accessed 12 April 2023).

27 Heidolph Titramax 1000 package Shakers - Microplate 544-12209-00, https://www.wolflabs.co.uk/laboratory-products/shakers-microplate/10001619, (accessed 26 September 2024).

28 Deep well collection plates, 96-Well, 2,2 ml, https://uk.vwr.com/store/product/15966666/deep-well-collection-plates-96-well-2-2-ml, (accessed 26 September 2024).

29 A. M. Castilla, T. K. Ronson and J. R. Nitschke, *J. Am. Chem. Soc.*, 2016, **138**, 2342–2351.
